# Disrupting Amyloid Filaments of Tau by Means of Electric Fields

**DOI:** 10.1021/acs.jpcb.5c04393

**Published:** 2025-08-27

**Authors:** Pablo Andrés Vargas-Rosales, Giuseppe Giangreco, Amedeo Caflisch

**Affiliations:** Department of Biochemistry, 27217 University of Zurich, Winterthurerstrasse 190, Zürich, ZH 8057, Switzerland

## Abstract

The tau protein is a major component of neurofibrillary tangles, one of the hallmarks of Alzheimer’s disease, which is the most common neurodegenerative disorder in the elderly. Experimental and computational studies have shed light on the fibrillar morphologies of tau and the kinetics of self-assembly, but little is known about the structural stability of the fibrils in the presence of external electric fields. We investigated the behavior of cross-β filaments of tau under the effect of an oscillating external electric field by means of multiple molecular dynamics simulations. Two models of the aqueous solvent were used: explicit water and implicit solvent based on the continuum dielectric. The simulations started from tau filaments with two different topologies determined by cryogenic electron microscopy of patient samples: the so-called straight filament (SF) and paired helical filament (PHF). Two values of the electric field strength and oscillation frequencies of 0.1, 1, or 10 GHz were employed. In all simulations, tau segment 340-KSEKLDFKDRV-350, which includes seven charged side chains, showed pronounced flexibility, which was exacerbated at high field strength. A larger loss of β-strand content was observed for the SF than for the PHF topology. Moreover, the SF assembly dissociated into two protofilaments in the presence of the external field, which was not observed for the more stable PHF topology. The loss of β-sheet structure was highest at the frequency of 1 GHz and smallest at 10 GHz in the explicit water simulations, while mixed decays of β-sheet content were obtained with the implicit solvent.

## Graphical abstract

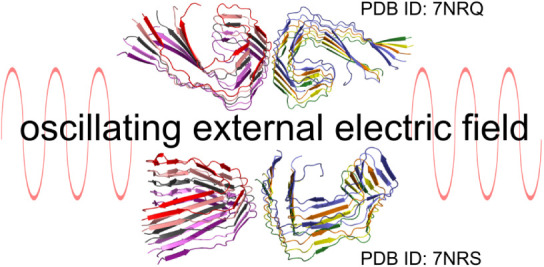

## Introduction

Tau is an intrinsically disordered protein essential to cellular function due to its interaction with microtubules. Its functions include the polymerization of tubulin, intracellular trafficking, and the stabilization of microtubules, among others.^1^ Under abnormal conditions, tau can pathologically aggregate, causing a set of disorders collectively known as tauopathies. One important tauopathy is Alzheimer’s disease (AD), and one of its hallmarks is the appearance of neurofibrillary tangles (NFTs), composed mainly of aggregated tau. AD is a multifactorial neurodegenerative disease causing an estimate of 60–80% of all cases of dementia.^2^ Another hallmark of AD is the accumulation of aberrant amyloid precursor protein in the form of amyloid-β (Aβ) plaques inside neurons. The tau toxicity model is connected with the amyloid theory as the presence of Aβ plaques is followed by an increase in tau phosphorylation. This causes the disruption of microtubules and eventually neuronal death. Other theories of the causes of AD include the acetylcholine hypothesis, the glutamate toxicity hypothesis, blood supply deficiency, and a recent hypothesis on the possible effects of viruses invading the nervous system or causing inflammation in the brain.^3^ Regardless of the different theories on its causes and mechanisms, patients with AD show an accumulation of Aβ plaques and NFTs in the brain.^4^ Therefore, the onset of AD is defined by a series of biomarkers categorized mainly into the deposition of Aβ, the appearance of pathological tau, and external signs of neurodegeneration.^2^ There are two different arrangements of tau filaments in NFTs: straight filament and paired helical filament (SF and PHF, respectively; Figure 1). Their names come from the way they look in negative staining microscopy. PHFs were the first to be described as the main components of NFTs,^5^ but later, it was discovered that NFTs contain both PHFs and SFs, with a higher proportion of PHFs.^6^ The proportion of PHFs to SFs has been reported to be approximately 90%/10%.^7^ The first near-atomic resolution cryoelectron microscopy structures of tau filaments extracted from AD patients confirmed that subunits (protofilaments) have the same fold irrespective of the filament topology.^8^ In-tissue cryogenic electron tomography studies of tau fibrils in the context of AD revealed a specific arrangement of parallel fibrils, with spatially confined characteristics such as the PHF-SF ratio or the twist of the fibrils.^9^ Tau fibrils with the same conformation as those obtained from the brains of patients have also been produced in vitro.^10^

**Figure 1:**
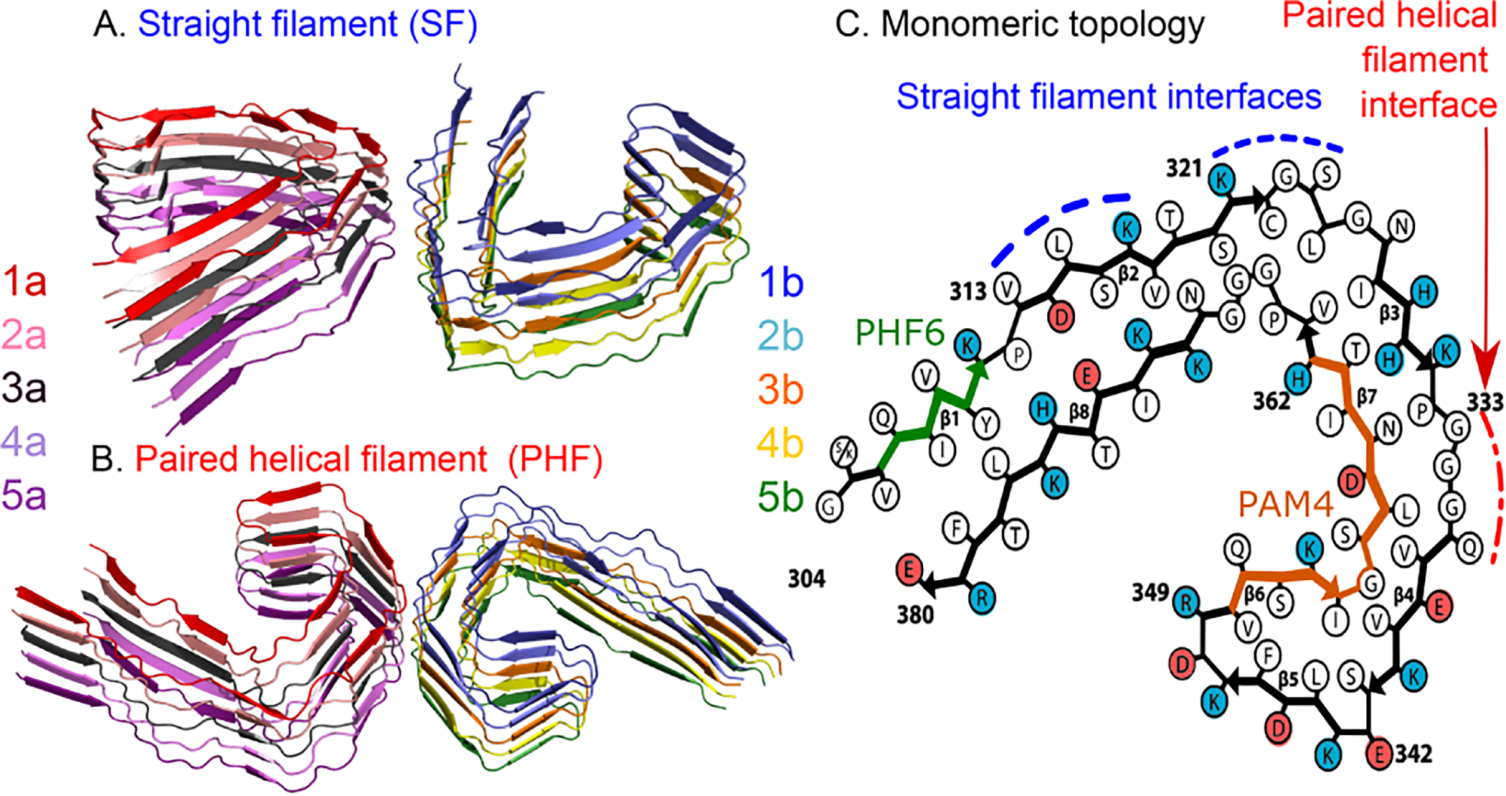
Structures of the simulated systems. The filament core consists of 10 peptides, i.e., two protofilaments of five peptides each. Each peptide is a c-shaped structure, but the interactions between the two protofilaments vary depending on the topology. (A) The paired helical filament (PHF, PDB ID: 7NRQ). (B) The straight filament (SF, PDB ID: 7NRS). (C) The topology of each monomeric peptide in its c-shaped conformation is common to both assemblies, but the SF has an asymmetric interface (blue), while the PHF is symmetric (red). In the fibrillar core of tau, positively charged residues (shaded blue) are more abundant than negatively charged ones (shaded red). Two important amyloidogenic motifs are emphasized: PHF6 (residues 306–311, green) and PAM4 (residues 350–362, orange). Adapted from ref 44. Available under a CC-BY-NC license. Copyright 2018 Goedert et al. Published by Cold Spring Harbor Laboratory Press.

Molecular dynamics (MD) simulations have been extensively used to study tau protein, especially to investigate its aggregation pathways. Different regions of the amyloid core of tau have a high aggregation potential and have been studied with particular attention, for example, the PHF6 region (306-VQIVYK-311)^11^ and the PAM4 (polymorphic amyloid motif of repeat 4, i.e., 350-VQSKIGSLDNITH-362).^12^ Simulation studies have provided evidence that PHF6 is essential for aggregation,^13,14^ and this finding has also been supported by experimental data.^15,16^ The influence of diverse posttranslational modifications on the structure,^17^ function,^18^ and aggregation^19–21^ of tau has been reported. Many of these studies only focus on monomeric fragments or oligomers of tau.^22,23^ Leonard et al. studied tau self-assemblies containing ten peptides,^17^ and He et al. simulated a full-length tau protein monomer.^24^ Another study also simulated the fibrillar core of tau, adding part of the N-terminal fuzzy coat, and showed the effect of different mutations on the structural behavior of tau.^25^ Other research includes the interaction of tau with membranes,^26–28^ microtubules,^29^ Aβ,^30,31^ or small molecules,^32^ which have been reviewed recently.^33^

For a long time, there has been interest in the effect of electric fields on protein structure. The application of electric fields began experimentally. For example, Zhao and Yang showed that the degradation of the secondary structure of lysozyme caused by electric fields is different from that caused by thermal effects.^34^ Complementarily, Budi et al. used MD simulations to understand the degradation of insulin under diverse electric fields.^35^ A later study proposed that there exists a specific electric field strength threshold at which protein structure is disrupted.^36^ Such an insight opened the door to the use of electric fields to perturb the structures of protein aggregates. Further experimental results suggest that electric fields can slow down the aggregation of amyloidogenic peptides of tau.^37^ In another study, electric fields were applied to *Drosophila* models of sleep disease caused by protein aggregation, obtaining encouraging results.^38^ MD simulation studies involving electric fields were reviewed as early as 10 years ago.^39^ In recent years, simulation studies by others and us have focused on the effects of static and oscillating electric fields on Aβ42.^40–42^ In another example related to neurodegenerative disease, α-synuclein fibrils were shown to lose their structure under static electric fields of different strengths, especially those starting at 300 mV/nm.^43^

Simulations of biological macromolecules can make use of models at different levels of details, ranging from the full electronic description obtained with quantum mechanical simulations, through atomistic descriptions in classical molecular dynamics, to coarse-grained modeling of molecular ″beads″ of different sizes. In atomistic simulations, further simplification can be obtained by avoiding the explicit treatment of solvent molecules, i.e., by treating the water molecules as a mean-field effect. This results in a considerable reduction in the calculations needed for each time step, as water molecules usually require about 90% of the computing time. Moreover, the lack of friction at the surface of the solute results in faster transitions.^45^ The acceleration in the rates of conformational change in implicit solvent simulations has been reported to vary, with a speed-up for large conformational transitions of up to a factor of 100 with respect to explicit solvent.^46,47^ Both types of simulations can be used to study the dynamics of proteins^48,49^ and peptides,^50^ investigate ligand binding,^51^ and estimate binding free energy.^52,53^

The implicit model of solvation has been widely applied to understand and predict the behavior of intrinsically disordered proteins (IDPs) and their folded and unfolded states.^54^ One of the first implicit solvent simulation studies of an amyloidogenic peptide revealed the key role of the side chains in the initial formation of the cross-β structure.^55^ Here, we make use of the fast analytical continuum treatment of solvation (FACTS),^56^ which is one of the several generalized Born models in the CHARMM suite for molecular simulations.^57,58^ FACTS simulation results were validated by NMR spectroscopy in a study of the influence on aggregation of different Aβ_12–28_ inhibitors.^59^ A limited number of studies of tau have been conducted in implicit solvent,^54^ e.g., stochastic conformational searches of monomeric tau.^60^ Enhanced sampling simulation protocols have played an important role in the study of tau aggregation due to its large size and the long time scales of self-assembly. Recent advancements in the study of tau protein and its interactions under high rates of mechanical stress using implicit solvent have revealed its viscoelastic properties and strong tau-tau and tau-microtubule binding, which are relevant to traumatic brain injury and neurodegeneration.^61^

Previously, we studied the degradation of dimeric Aβ42 in the presence of oscillating external electric fields (oeEFs).^40^ Here, we explore the effect of oeEFs on the fibrillar core of tau protein. To the best of our knowledge, these are the first simulations of tau under the influence of an external electric field. We use two topologies (SF and PHF), which originate from tissue samples extracted postmortem from the brains of patients.^62^ The SF and PHF simulation systems are each composed of a cross-β assembly of 10 peptides (Figure 1). For each of the two topologies, we make use not only of different field strengths (100 and 200 mV/nm) but also varying oscillation frequencies (0.1, 1, and 10 GHz), in addition to a control without an external field. At each combination of field strength and frequency, explicit water and FACTS^56^ implicit solvent simulations were carried out. The motivation for running the simulations with the FACTS model was 2-fold. First, we wanted to analyze whether the FACTS model preserves the fibrillar topologies in the absence of an oeEF. Second, we were interested in comparing whether the two models of solvation give consistent results under the influence of an oeEF. Note that the wall time required for a 100 ns simulation with explicit solvent or FACTS is similar because the water box has nearly an order of magnitude more atoms than the solute, and thus it scales better on parallel computers than any solute in implicit solvent. However, the FACTS simulations require a substantially smaller number of cores (a factor of about five fewer; see the benchmarks below). Thus, the total computational cost for FACTS sampling is substantially smaller.

The oeEFs used in the present simulations have a very high field strength, stronger than those that would be feasibly applied to brain tissue. This choice is justified by the much shorter time scales accessed using molecular simulations compared to the weaker field strengths and longer time scales that can be applied experimentally. This is analogous to pioneering simulation studies of protein unfolding in the mid-1990s, which used extremely high temperatures to accelerate the sampling of protein denaturation.^63,64^ It is an advantage of simulations that temperatures above the boiling point of water or extremely high electric field strengths can be employed to investigate slow processes.

## Methods

The effects of the oscillating external electric field (oeEF) on the dynamics and structure of the fibrillar core of tau protein fibrils were studied by using both explicit solvent and implicit solvent simulations. The initial structures originated from two cross-β topologies determined by cryogenic electron microscopy (cryoEM) using postmortem extracts of brains from patients. One is the so-called PHF (PDB ID: 7NRQ) and the other is the SF (PDB ID: 7NRS).^62^ Each decameric filament is composed of two pentameric protofilaments, which interact with each other. PHF and SF protofilaments possess the same structure but differ at the interface between the protofilaments (Figure 1).^6,44^ In the present simulation study, both topologies contain the same number of solute atoms (10 peptides of 77 residues each). Each of the 10 peptides consists of tau residues G304-E380, which form the fibrillar core in the cryoEM structures. To investigate the influence of the oeEF on the structural stability of the cross-β filaments, simulations were carried out at oscillation frequencies of 0.1, 1, and 10 GHz and field strengths of 100 and 200 mV/nm. Simulations without an electric field were performed as controls with both models of the solvent.

### Explicit Solvent Simulations

The starting structures were prepared using CAMPARI v5.^65^ The *N*- and C-termini of each peptide were capped with acetyl and *N*-methylamide, respectively, to avoid spurious formal charges. Explicit solvent simulations were carried out using the GROMACS 2021.5 software^66^ and the July 2022 GROMACS port of the CHARMM36m force field.^67^ The structures were solvated in cubic boxes of TIP3P (CHARMM)^68^ water molecules, 17.03 and 14.48 nm in size for the PHF and SF topology, respectively. Periodic boundary conditions (tridimensional in a cubic box) were applied to avoid edge effects. In the case of a static, i.e., nonoscillating electric field, a net dipole resulting from alignment with the external field will interact with the analogue dipoles in the periodic images.^69,70^ This potential artifact is negligible for electric fields oscillating at a high frequency. Ions of Na^+^ and Cl^–^ were added to neutralize charges up to a concentration of 0.150 M. Afterward, the system was subjected to energy minimization. A 5 ns NVT equilibration was performed, in which the system was kept under positional restraints, to reach 300 K. The strength of the positional restraints was halved every nanosecond, from 10 to 0.67 kcal/(mol Å^2^) to ensure relaxation of the structures. Production runs were carried out in the canonical (NVT) ensemble using a velocity-rescale thermostat with a coupling time of 1 ps. The number of independent runs and their lengths are listed in Table 1. The simulation protocol is the same as described in our previous study.^40^ The oeEF was applied using the respective GROMACS function,^69^ originating from the research described in ref 70. Practically, this meant defining the field strength (*E*_0_, units in V/nm) and the oscillation frequency (ω, units in ps^–1^ or THz). About 20 node-days (about 5760 core-hours) were required for 100 ns of production run of the PHF topology on a hybrid Xeon E5-2690 v3 at 2.60 GHz (12 cores, 64 GB RAM) + Tesla P100 16 GB.

**Table 1:** Explicit Solvent Sampling

Field		PHF	SF
Frequency (GHz)	Strength (mV/nm)	Length (ns)	Length (ns)
	No field	16 × 110	16 × 120
		8 × 400	8 × 400
0.1	100	12 × 110	12 × 120
		4 × 280	4 × 390
	200	12 × 110	12 × 120
		4 × 280	4 × 390
1	100	16 × 110	16 × 120
		8 × 400	8 × 600
	200	16 × 110	16 × 120
		8 × 400	8 × 600
10	100	12 × 110	12 × 120
		4 × 280	4 × 390
	200	12 × 110	12 × 120
		4 × 280	4 × 390

### Implicit Solvent Simulations

The structures of the two decameric systems were prepared for simulation using the CHARMM-GUI.^71^ The *N*- and C- termini were capped with neutral groups, as in the explicit solvent simulations. Implicit solvent simulations were carried out using the CHARMM c49b1 software^58^ and the CHARMM22* all-hydrogen force field, which is required by FACTS. CHARMM22* has been proven to be effective in simulating large IDPs.^72^ FACTS is an efficient Generalized Born implicit solvent model that accounts for the electrostatic contribution to solvation.^56^ This model is based on the fully analytical evaluation of the volume and spatial symmetry of the solvent displaced from around a solute atom by its neighboring atoms. An ionic strength of 0.150 M was approximated using the linearized Debye–Hückel model.^73^ The nonpolar contribution to the total effective energy was approximated by a term proportional to the solvent-accessible surface area of the solute, using a surface tension-like multiplicative parameter of 0.015 kcal/(mol Å^2^). The system was confined within a periodic cubic box with dimensions equivalent to those employed in the explicit solvent protocol for both the PHF and SF topologies. Subsequently, the system underwent energy minimization. A heating phase lasting 1 ns was conducted, during which the system was maintained under positional constraints with a value of 40 kcal/(mol Å^2^), and the temperature was raised from 200 to 300 K. The strength of the positional restraints was systematically reduced every nanosecond, from 20 down to 1.25 kcal/(mol Å^2^) to ensure the relaxation of the structures. Production runs in the canonical ensemble were carried out employing a Langevin integrator with low friction (coefficient of 0.15 ps^–1^) and a time step of 2 fs (Table 2). About 20 node-days (7600 core-hours) were required for 100 ns of production run using 16 MPI processes on one 64-core EPYC-7702 compute node.

**Table 2:** Implicit Solvent Sampling

Field		PHF	SF
Frequency (GHz)	Strength (mV/nm)	Length (ns)	Length (ns)
	No field	3 × 83	3 × 103
0.1	100	3 × 106	3 × 103
	200	6 × 98	6 × 103
1	100	3 × 104	3 × 99
	200	3 × 100	3 × 105
10	100	3 × 96	3 × 102
	200	3 × 97	3 × 99

### Analysis

We used the MDTraj Python package^74^ to calculate the interatomic distances, contacts, and the secondary structure (DSSP^75^ algorithm). Three types of contacts have been studied. First, intrapeptide contacts are defined as pairs of residues with Cα atoms located within 1.25 nm of each other, belonging to the same peptide chain (e.g., both residues in chain 1a), which are also more than three residues away along the sequence. Interpeptide contacts are the pairs of residues whose Cα atoms are located within 0.8 nm of distance, belonging to different peptide chains (irrespective of the protofilament in which they are located). Interprotofilament contacts are the pairs of residues where their Cα atoms have a distance of less than 1 nm and are located on different pentameric assemblies (corresponding to different protofilaments).

Root mean square fluctuations (RMSF) are calculated with a self-written function (see the Supporting Information). For this calculation, we align the structure to the central chain of each pentameric system (i.e., chain 3a or 3b) and calculate the fluctuations for each individual chain. The interface calculation was performed in PyMol, using the ″get_area″ command, with ″dot_solvent″ = 1 and ″dot_density″ = 4. The area of the decameric complex was subtracted from the sum of the individual pentameric assemblies to obtain the interface area.

The kinetics of β-sheet decay were fitted with one- and two-phase exponential functions. Four different models were tested: one parameter (one phase, *e* ^–*x*/*B*^), two parameters (*Ae* ^–*x*/*B*^), three parameters (two phases, *Ae* ^–*x*/*B*^ + (1 – *A*)*e* ^–*x*/*D*^), and, finally, four parameters (two independent phases, *Ae* ^–*x*/*B*^ + *Ce* ^–*x*/*D*^). The half-life values for the two-phase models were calculated from the slow phase. An unpaired, two-sample Student’s *t* test was used to quantify the significance of the differences in β-strand content between conditions, and the scipy implementation^76^ was used.

## Results

We first present the explicit solvent simulations, followed by the FACTS implicit solvent runs.

### The PHF Topology is More Stable Than the SF

The control simulations in the absence of oeEF show that both decameric self-assemblies are structurally stable on a time scale of about 0.5 μs (left panels of Figures 2 and 3).

**Figure 2:**
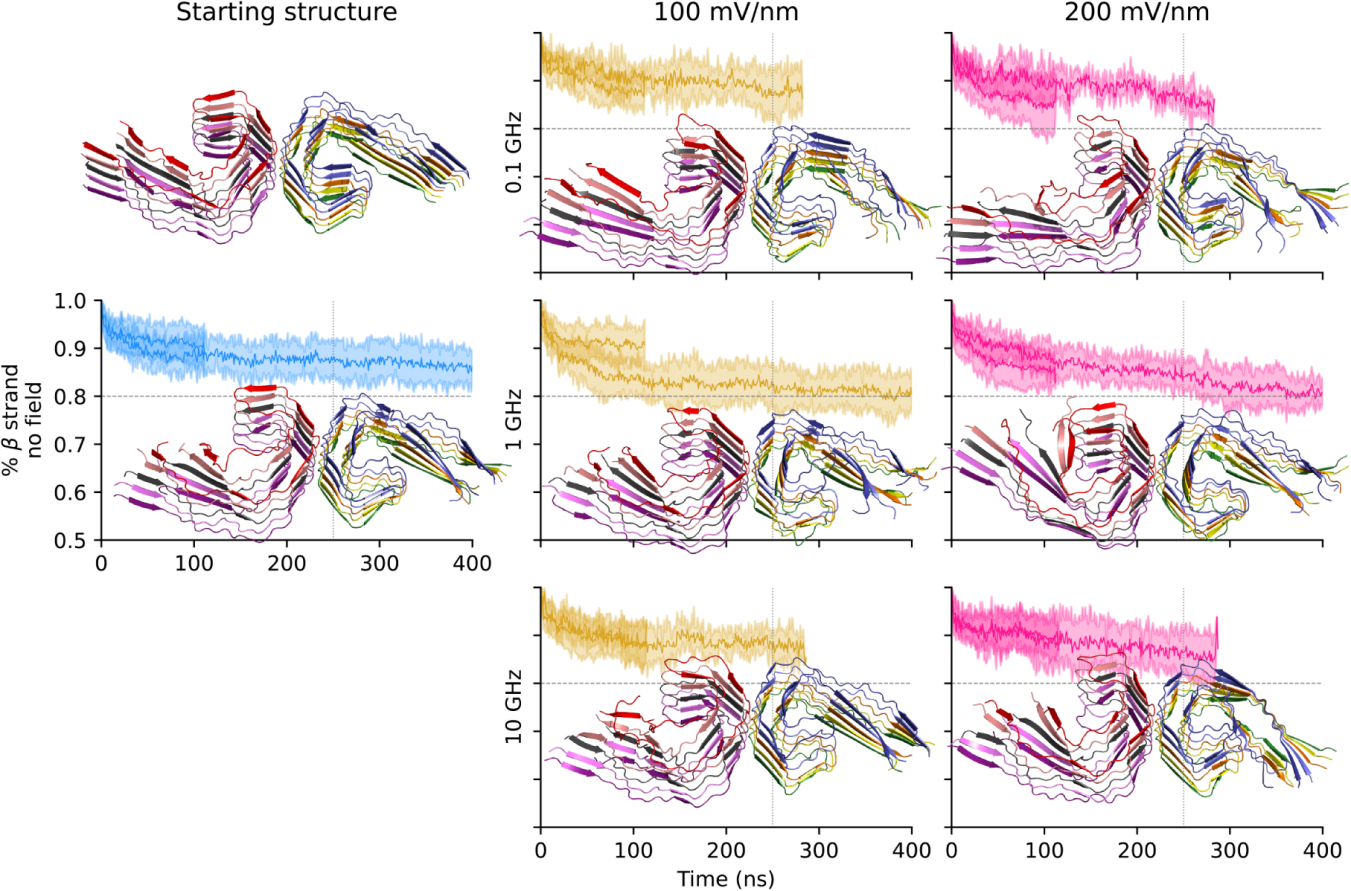
PHF topology is stable under the oeEFs in explicit solvent simulations. The mean β-strand content is shown for each of the seven conditions (solid lines) together with the standard deviation (colored bands). Representative snapshots (ribbon representation) were extracted after 250 ns (vertical lines). The initial structure (top left) is also shown.

**Figure 3:**
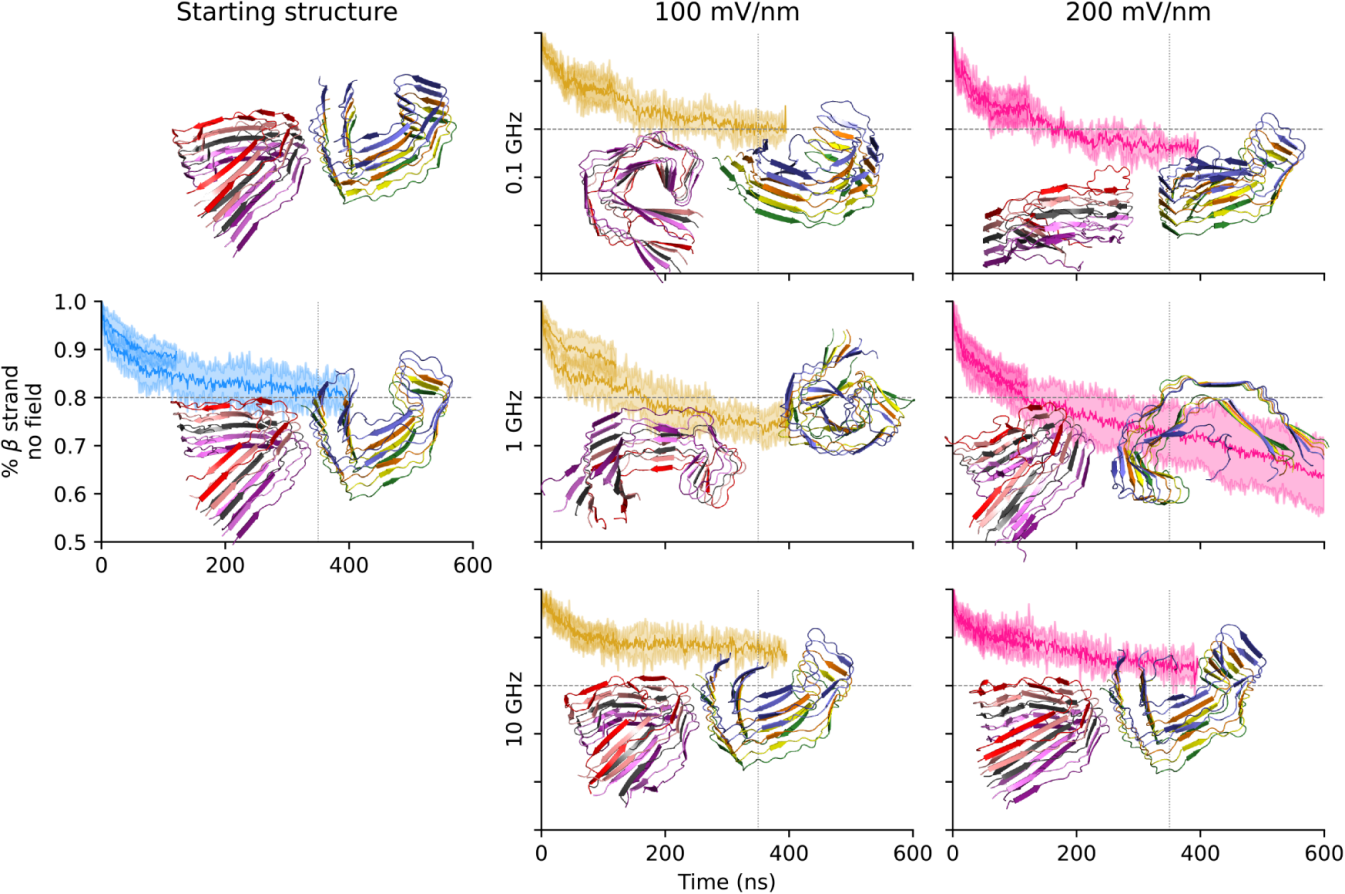
Decameric SF assembly separates into two pentameric protofilaments at frequencies of 0.1 and 1 GHz. Same as Figure 2, except for the representative structures, which are extracted at 350 ns instead of 250 ns.

We monitored the degradation of secondary structure to analyze the effects of oeEFs of different strengths and frequencies on the structure of the tau protofibrils (Figures 2 and 3). In the control runs without oeEF, there is a more pronounced loss of β-strand content for the SF topology (nearly 20% loss) than for the PHF topology (about 10%). A similar difference in stability is observed in the presence of an oeEF at 1 GHz, which shows a decay of β-strand content of about 30% and nearly 20% for SF and PHF, respectively. To assess the statistical significance of the β-strand decay, we compared the simulations under the field of 200 mV/nm oscillating at 1 GHz with those without oeEF, at the simulation times of 250 and 350 ns for the PHF and SF topologies, respectively. A Student’s *t* test for comparing the means yields a *P* value < 0.05 for the PHF and <0.01 for the SF. Similar statistical robustness was measured for the comparison of the control simulations with the oeEF of 100 mV/nm and 1 GHz (p-value < 0.05 for the PHF and <0.01 for the SF). At 0.1 GHz, only the SF topology under a field strength of 200 mV/nm shows a larger decay (about 25%) than the control runs, while the simulations at 10 GHz and both field strengths show a similar decay as in the control.

In all simulations, irrespective of the field, there is an increase in the twist of the β-sheets at the *N*- and C-terminal segments (Figure 4). The larger twist is likely to originate from finite-size effects and/or spontaneous relaxation of the model built from the cryo-EM density. The increase in the β-sheet twist is exemplified for the control and oeEF runs in the snapshots presented in Figure 5. The flexibility of the termini, which is consistent with the twist increase, is shown in the RMSF profiles for both topologies at a 1 GHz oscillation (Figure 6).

**Figure 4:**
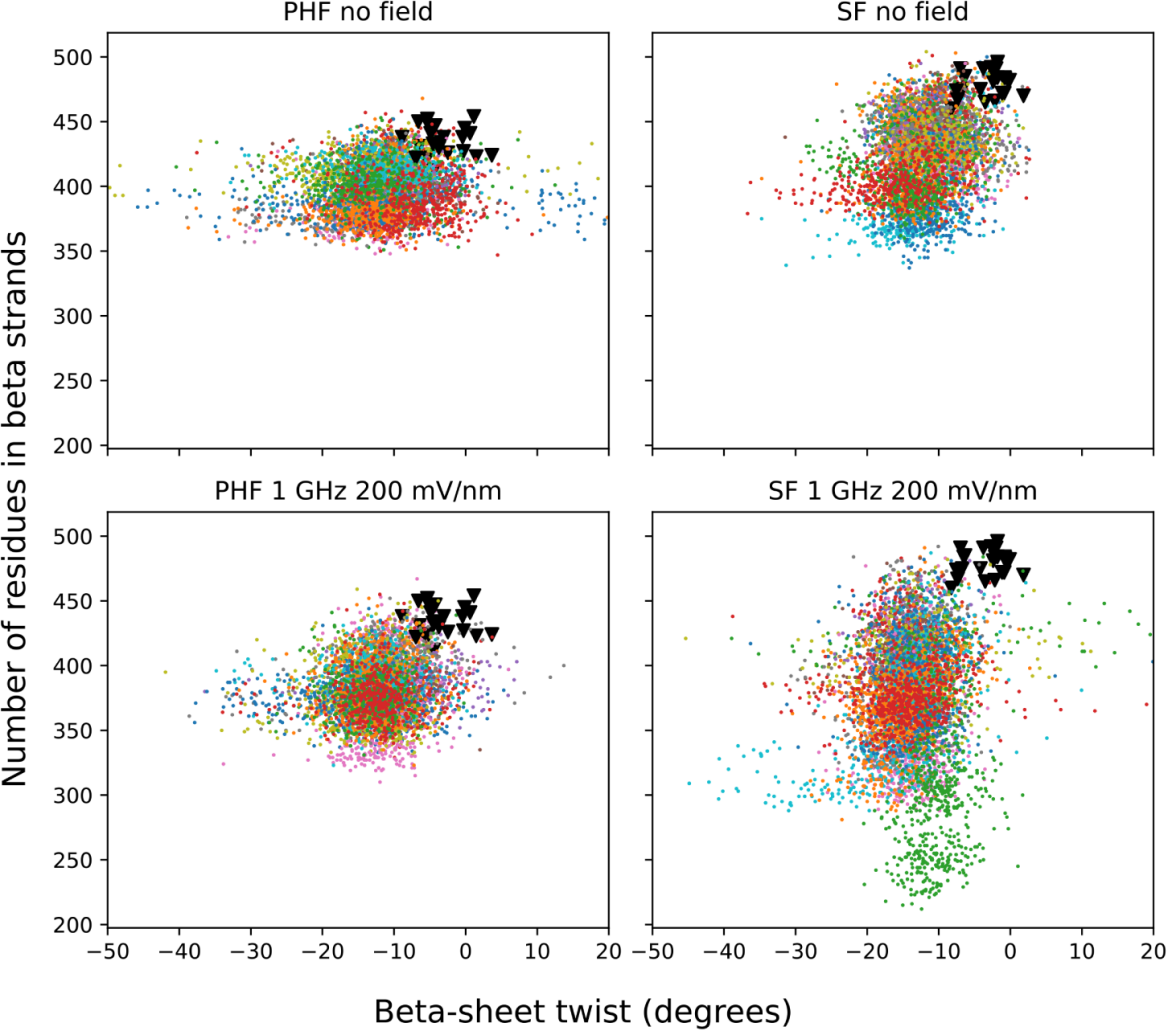
Scatterplot of the β-sheet content vs β-sheet twist. In all runs (different colors for each trajectory), there is an increase in the twist from the starting structures (black triangles). Furthermore, the twisting of the β-sheet is not correlated with the amount of secondary structure. The twist is calculated by the dihedral angle between the Cα atoms of adjacent peptides, more precisely, V306–Y310 of peptide 1a and Y310–V306 of peptide 2a.

**Figure 5:**
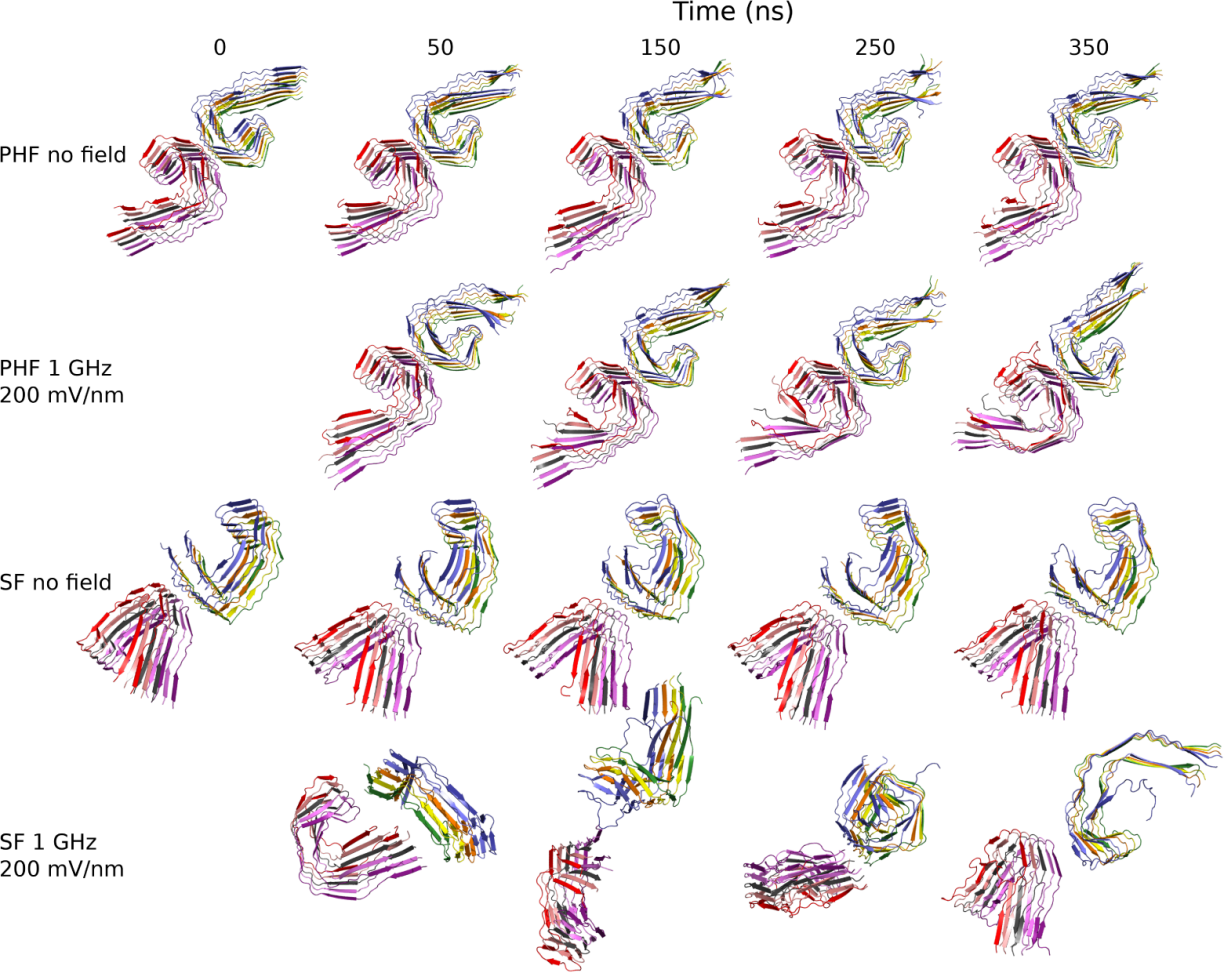
Time evolution of the PHF and SF in control simulations and in the presence of an oeEF of 200 mV/nm and a 1 GHz oscillation frequency. The snapshots are extracted from representative MD simulations.

**Figure 6:**
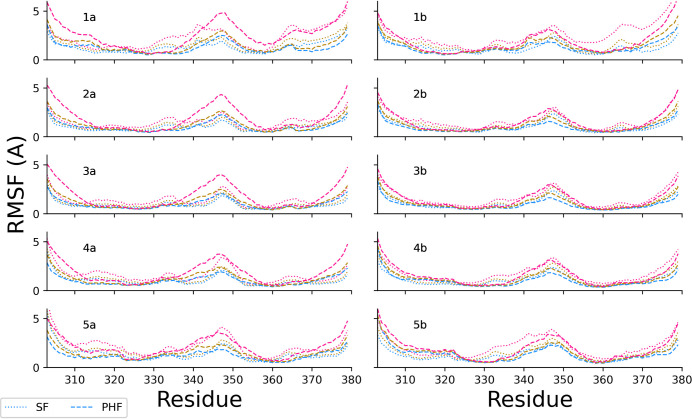
RMSF profiles for each peptide of the PHF (dashed) and SF (dotted) decameric assemblies. Each panel shows sequence profiles for each of the 10 peptides of the decameric assembly (1a–5a, left; 1b–5b, right). The colored lines show the RMSF profiles of the control runs (blue) and the simulations with 1 GHz oeEF at 100 mV/nm (gold) and 200 mV/nm (pink).

Overall, the more voluminous topology (PHF, Figure 2) is more stable than the more compact one (SF, Figure 3) under any combination of field strength and frequency, except for the simulations at 10 GHz that show a similar stability of the two assemblies. Although the protofilaments in PHF and SF have the same conformation, the packing between the two protofilaments differs. One possible explanation for the separation is the smaller contact surface between the two protofilaments in the SF topology, with about 1000 Å^2^ of interface area versus 2200 Å^2^ in the PHF. The symmetric binding of PHFs is stabilized by hydrogen bonds between the backbone atoms of 332-PGGGQ-336 on both fibrils. There are additional favorable polar interactions between the side chains of K331, Q336, and E338. Meanwhile, in SFs, there are only apolar interactions between 313-VDLSK-317 of one protofilament and 321-KCGS-324 of the other.^44^ The rapid loss of these interactions is exemplified in Figure 7 (green lines), where the peptide 3a of the SF loses its interfibrillary contacts in the 200 mV/nm simulation and also for all individual peptides in Figures S1–S7 for PHF and Figures S8–S14 for the SF). This behavior is consistent with a previous simulation study of tau in the absence of oeEF.^77^

**Figure 7:**
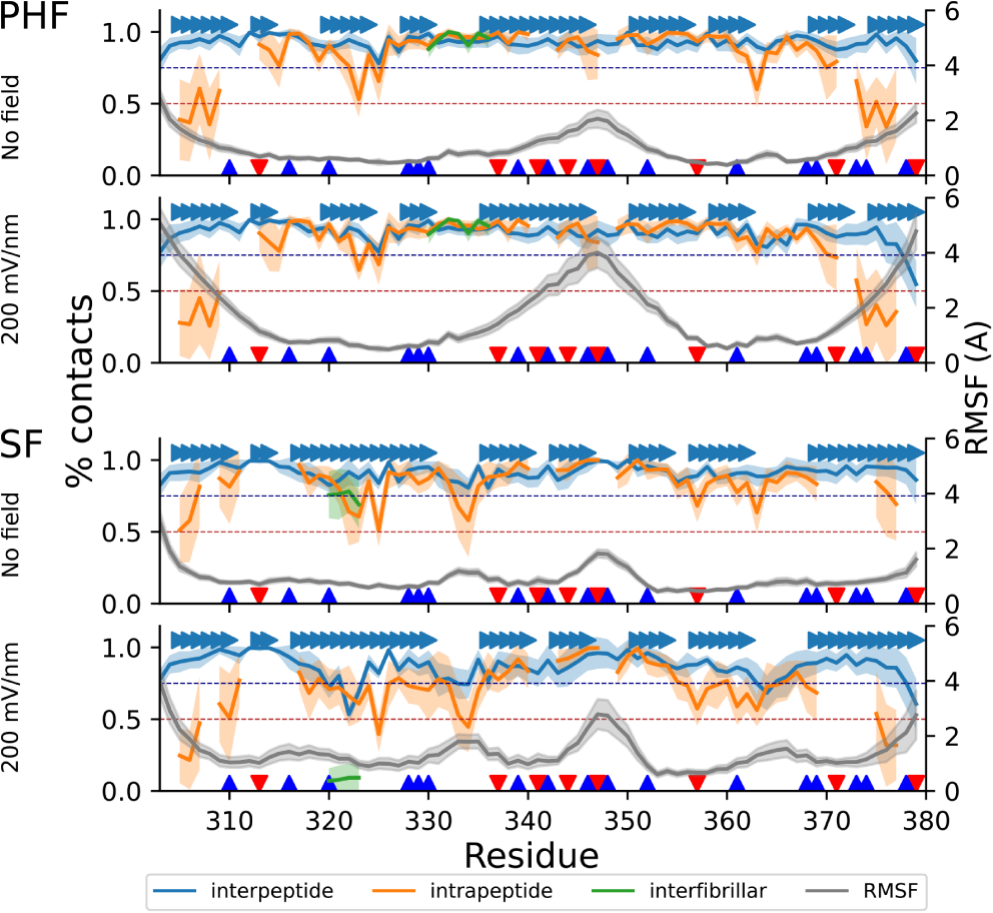
Fraction of interpeptide (blue), intrapeptide (orange), and interfibrillar (green) contacts, as well as the structural flexibility (gray, RMSF labels on the *y*-axis on the right), for peptide 3a, without and with 1 GHz 200 mV/nm oeEF in the explicit solvent runs. Peptide 3a is located in the middle of the decameric assembly. Solid lines show the mean over all trajectories, while the colored bands represent one standard deviation. Individual β-strands of the cryoEM structure are also shown (arrows). Positively and negatively charged residues are labeled (blue and red triangles). The application of the oeEF results in an increase in intrinsic flexibility. In the SF topology, the application of the field separates the fibrils, resulting in an almost complete loss of interfibrillar contacts.

It is interesting to note that an additional density appears in the CryoEM maps, which was suggested to help stabilize the SF fibrils. It was theorized that it could correspond to the N-terminal segment 7-EFE-9.^8^ We have carried out simulations of the SF structure with this tripeptide in the region of the density, but the small peptide was not stable at the interface between protofilaments (see Figure S86). Several authors propose that this density corresponds to ubiquitin^78,79^ or heparin.^80^ In any case, the relative instability of the SF decameric assembly, the existence of the positive charges of K317 and K321 in close proximity, and the small interface area indicate that the fibrillar core of the SF topology is marginally stable.

The degradation of the secondary structure is a complex process that depends on several factors, both related to the oeEF, such as the field strength or the oscillation frequency, and those related to the protein, including volume, ability to align itself to the oeEF, and composition. In previous simulation studies, it was shown that chignolin, a 10-residue β-hairpin peptide, aligns its dipole with that of the oeEF and responds particularly to the application of near-perpendicular oeEFs.^81^ The SF topology, being smaller than the PHF, might be more capable of rotating to align its dipole with the oeEF, with the friction exerted by the solvent allowing this rotation to be delayed enough to suffer a degradation of the secondary structure.

This can also explain why the PHF, more voluminous than the SF, is more resistant to the oeEF as it realigns less rapidly to the oeEF. The separation of the pentameric subunits of the SF indicates that they react individually to the oeEF, yielding even less voluminous subunits.

The simulation results obtained at different frequency values provide information about the influence of the frequency on the stability of the (proto)­filaments. As mentioned above, for the SF topology, the β-strand content decreases significantly for fields oscillating at 0.1 and 1 GHz, while at 10 GHz, it remains similar to the simulation without oeEF. This is quantified by the half-life of the β-strand content, which is largest at 10 GHz (Table 3). The SF at 100 mV/nm oscillating at a frequency of 10 GHz has a half-life similar to that of the control simulation, while the fastest degradation is observed at 1 GHz for both field strengths. One potential explanation is that at 10 GHz, the change in the direction of the field (every 0.1 ns) is too fast for the peptide chains to move under its influence or weaken the favorable polar interactions within the solute. The observation is consistent for both topologies and is congruent with recently reported simulations of Aβ_16–22_, where high-frequency fields of 10 THz resulted in a marginal effect on the oligomer plaques.^42^ Studies in insulin also reported a strong dependence on the frequency of the oscillating field, with oeEFs at lower frequencies showing effects similar to those of static fields.^82^ For PHF, there is a considerable acceleration in the decay of the β-sheet content at 1 GHz of oscillation. The 0.1 GHz frequency results in a half-life similar to that without a field (Table 3).

**Table 3:** Decay of the β-Strand Content

Field		PHF	SF
frequency (GHz)	strength (mV/nm)	half-life^a^ (μs)	half-life^a^ (μs)
	No field	6.2	4.1
		2.2	1.8
0.1	100	5.9	2.2
		2.8	0.9
	200	4.9	1.8
		4.5	1.2
1	100	2.0	1.5
		2.8	1.5
	200	2.3	1.5
		1.7	1.2
10	100	4.2	4.0
		1.7	0.8
	200	2.6	2.8
		1.5	1.9

a Half-life values of the slow phase in the two-phase three-parameter model (see Figures S58–S71 for the temporal evolution and fitting curves). For each combination of frequency and field strength, the top and bottom rows refer to explicit water and implicit solvent simulations, respectively.

The intrinsic flexibility of the backbone, as monitored by the profiles of RMSF, is lower at 10 GHz than at the two lower frequencies (see Section 3.2). This observation is consistent with the previously mentioned higher β-sheet content at 10 GHz. In the SF, there is also no separation of the decameric assembly in the 10 GHz simulations (Figure 3 cartoon representations). Budi et al. observed a similar dependence, where fields of the same strength have a higher effect at lower frequencies than at higher ones.^35^ In general though, we do not observe the ″plaque explosion″ that has been described for simpler systems, such as Aβ_16–22_.^42^ The decameric assembly of tau is much larger than the fragments used in the Aβ_16–22_ study and, therefore, is less susceptible to changes in field orientation, which are believed to promote, at least in part, the breaking of the secondary structure.^81^

Concerning the sequence of events, for both PHF and SF, the structural decay starts at the termini of individual peptides and mainly the peptides in the ″1″ tip of the decameric assembly (Figures S30–S43). The increased flexibility, following a field-strength-dependent pattern, is also exemplified in the RMSF profile for the 1 GHz runs (Figure 6). The polarity of the fibrillar arrangement could be a cause of this behavior. In metadynamics-enhanced MD simulations of tau PHF (PDB 5O3L), a similar mechanism of unbinding of the C-terminal segment of the tip peptide has been observed in the absence of an electric field.^17^ A study of fibrils of Aβ_16–42_ has found a similar effect, in which the alignment of the fibrillar dipole moment to the direction of the static field is observed,^83^ as in the chignolin simulations.^81^ In the same study, it was reported that the larger the aggregates (from monomeric to pentameric Aβ_16–42_), the more resistant to disruption they are.^83^ This is also something we observe, where the relatively large systems remain particularly stable at all field strengths for the time scales we studied. As expected, and in accordance to previous reports,^83^ regions with a higher number of charged residues, for example, the turn of each peptide, respond more strongly to the electric field.

### Flexibility of the Termini and the Central Segment 340–350

The comparison of the RMSF profiles at different field strengths shows that the oeEF enhances the intrinsic flexibility measured in the absence of a field, in a field-dependent manner (Figure 6). This observation is consistent with previous simulations of dimeric Aβ.^40^ As mentioned above, the values of the RMSF are higher at the terminal segments of the peptides for all conditions examined (Figure 6). Figure 7 shows the stability of contacts and the flexibility of one central peptide in the decameric assembly without the oeEF and at 200 mV/nm. The intrapeptide contacts (Figure 7 orange traces) are unstable in the terminal segments of the individual peptides, while they are mainly preserved in most of the remaining part of the sequence. The contacts between protofilaments (Figure 7 green traces) break in the simulations started from the SF topology and not in those started from PHF. Besides the termini, segment 340-KSEKLDFKDRV-350 shows pronounced flexibility. It forms the turn in the C-shaped structure of the protofilament and contains a large fraction of charged side chains (7/11, namely four positively charged and three negatively charged), which respond strongly to the oeEF. Surprisingly, the flexibility of the turn of the monomers is higher in the PHF than in the SF assembly (Figure 6). This might originate from the fact that the PHF, being larger, cannot fully rotate and align with the field, while the SF pentameric assemblies can rotate and align themselves to the oeEFs.

A recent experimental study has analyzed the amyloid propensity of tau using a combination of biophysical methods and a library of peptides spanning the sequence of tau.^12^ Besides the previously recognized PHF6 (306-VQIVYK-311) motif,^11^ a new segment called PAM4 (polymorphic amyloid motif of Repeat 4, i.e., 350-VQSKIGSLDNITH-362) is involved in favorable energetic contributions that stabilize tau amyloids.^12^ The six-residue PHF6 and 13-residue PAM4 segments show low flexibility in our simulations and maintain their contacts in the (largely ineffective) high frequency of 10 GHz. Indeed, the interpeptide contacts, which also report on the stability of the β-sheet along the fibrillar axis, are maintained under all conditions for the PAM4 region (Figure 7).

### Implicit Solvent

The control simulations without oeEF indicate that the PHF topology is quite stable on the 0.1 μs time scale and slightly more stable than that of the SF (Figures 8 and 9). In the presence of an oeEF, the decay of the β-strand content is faster for SF than for PHF, which is consistent with the explicit solvent. The stability of PHF is evident in the snapshots shown in Figure 8, where, after 100 ns of oeEFs in different conditions, they still strongly resemble the starting structure. Further comparing implicit with explicit solvent, one observes a less pronounced loss of β-sheet content in implicit solvent for the SF topology (only about 10% loss in Figure 9). Implicit and explicit solvent models exhibit markedly different decays of the β-strand content (Table 3) under the same field conditions, with implicit models generally producing more variable values of the half-life, especially in the PHF system. Despite the quantitative divergence between models, qualitative agreement on field-dependent destabilization is present. Limited comparability is likely due not only to the different models of the solvent but also to the significantly smaller sampling of the implicit solvent simulations (only three independent runs at each condition vs 16–24 independent runs at each condition with explicit solvent). No ″plaque explosion″^42^ is observed in the implicit solvent simulations, aligning with findings in explicit solvent and suggesting a general resilience of larger assemblies to oeEFs. Overall, the results confirm that oeEFs at 10 GHz are less disruptive to the secondary structure of the decameric assembly than those at lower frequency values.

**Figure 8:**
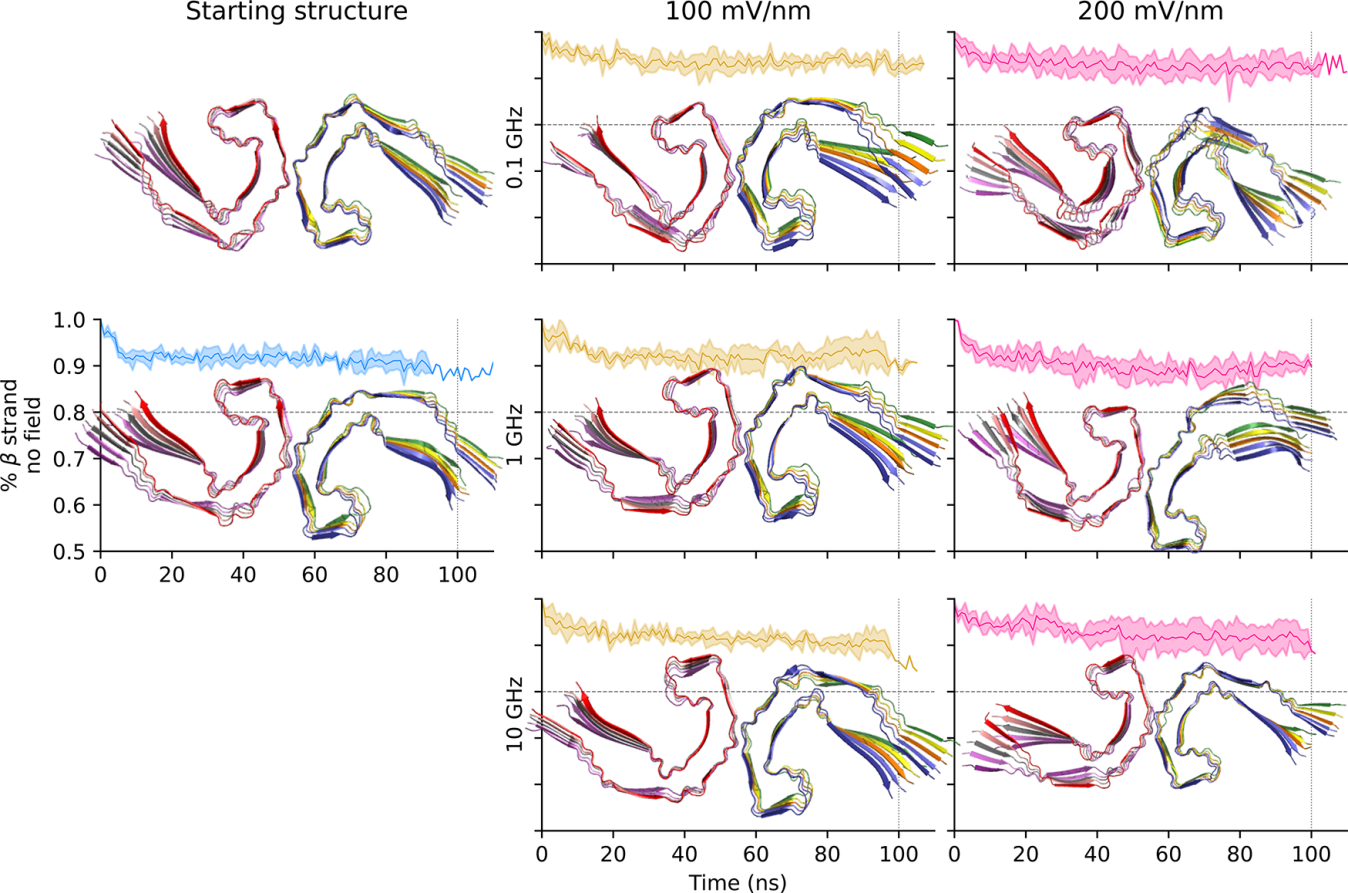
PHF topology is stable under the oeEFs in implicit solvent simulations. Same as Figure 2, except for the cartoon representation, which is extracted after 100 ns of simulation.

**Figure 9:**
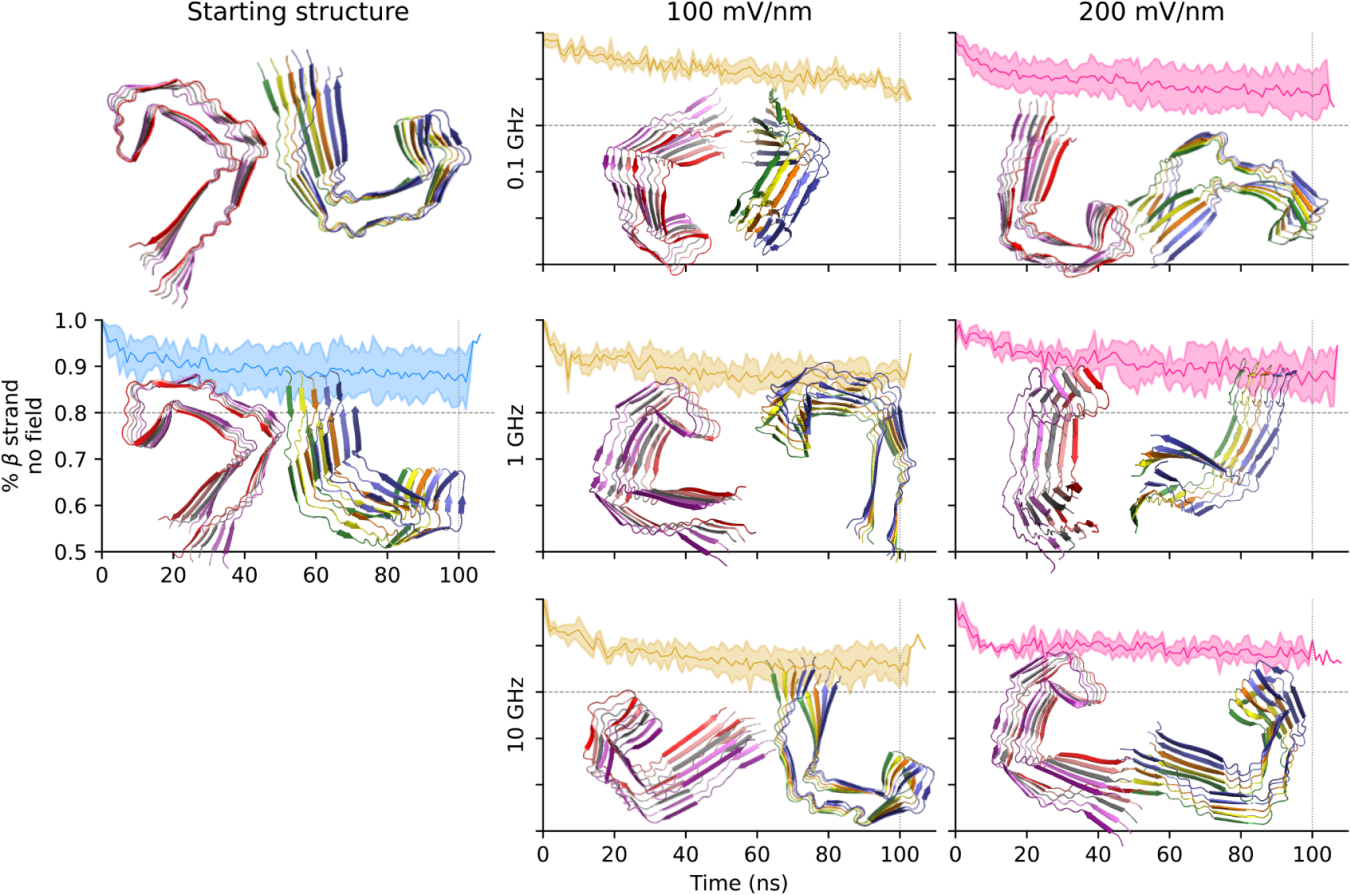
SF topology simulations in an implicit solvent. The cartoon representation of SF structures of arbitrary trajectories after 100 ns is shown.

For the simulations started from the PHF topology, the conformations are comparable between the explicit (Figure 2) and implicit solvent (Figure 8). In the case of the SF, it is more difficult to compare them (Figure 3 vs [Fig fig9]) due to the separation of the protofilaments. In some of the implicit solvent runs, the rupture of the SF interface resulted in a new, more extended arrangement of the two pentameric assemblies (see Figure 9), which was not observed in the explicit solvent runs. The new interface is sampled in some of the implicit solvent trajectories of SF, for instance, at 0.1 GHz and 100 mV/nm (two of three runs) or 1 GHz and 200 mV/nm (two of three runs). A similar extended conformation has been reported previously for individual protofilaments of SF in explicit solvent simulations using CHARMM27.^77^

In the implicit solvent simulations, the protofilaments (pentameric assemblies) of the SF topology deform, and the peptides lose their characteristic C-shaped topology, becoming more extended. This deformation is not present in the simulations with explicit water, where the dissociated protofilaments tend to take a more closed, almost globular form (Figure 3).

Similar to the explicit solvent simulations, at the lowest frequency (0.1 GHz), there is a marked increase in RMSF, as detailed in Figures S22–S28. Notably, at 0.1 GHz, the RMSF profile and intra/interpeptide contacts display higher standard deviations in the implicit solvent simulations compared to the explicit solvent, likely originating from the lack of friction and steric hindrance from the solvent. Consistent with explicit solvent simulations, certain regions of the system exhibit a greater structural variability than others. These fluctuations are comparable across both topologies. Among structural motifs, PHF6 and PAM4 remain the most resistant to the effects of the oscillating electric field (oeEF), although a decay of the intrapeptide contacts is observed for the PHF (Figure 10).

**Figure 10:**
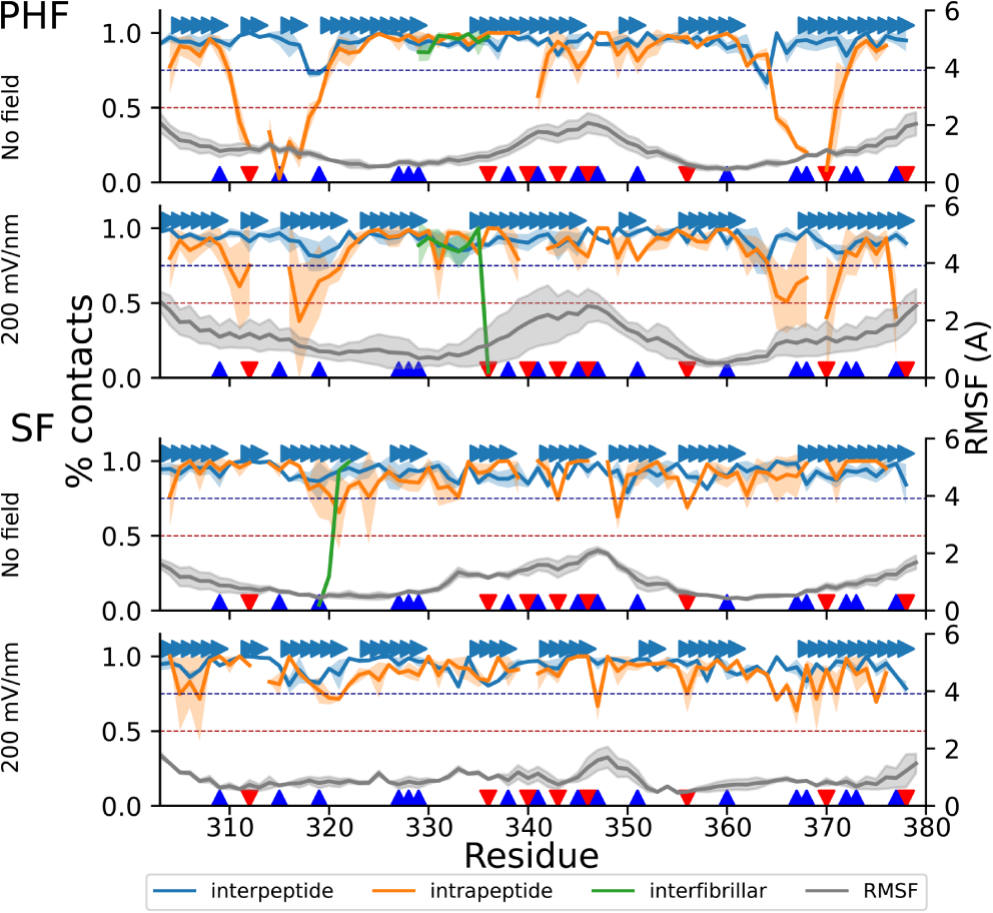
Analogous to Figure 7 for the implicit solvent. The 1-GHz 200-mV/nm oeEF does not substantially increase the intrinsic flexibility with respect to the control (gray lines with axis labels on the right). As for the explicit solvent, intrapeptide contacts (orange) are less stable than interpeptide, i.e., beta-sheet contacts (blue). For SF, there is a partial and complete loss of interfibrillar contacts for the control and under the influence of the oeEF, respectively (the green segment is visible only for the control runs of SF).

## Concluding Discussion

We presented an MD simulation study of amyloid filaments of tau under the influence of oscillating external electric fields. The simulations were started from two different filament topologies of tau (PHF and SF, respectively), which were determined by cryo-EM using brain extracts of patients.^8^ The MD simulations were carried out using six different conditions, namely, the possible combinations of three oscillation frequencies (0.1, 1, and 10 GHz) and two strengths of the electric field (100 and 200 mV/nm). Furthermore, two different models of the aqueous solvents were used. One main observation is that the PHF topology is more resistant to degradation than the SF topology under all field conditions and solvent models. This simulation result offers a plausible explanation for the larger amount of PHFs than SFs in the frontal cortex of a patient with AD, which resulted in higher-resolution cryo-EM structures of PHFs than SFs.^6,7,62^ The fibrillar core of tau degrades more rapidly and thoroughly under oeEFs with frequencies of 1 and 0.1 GHz rather than 10 GHz. Moreover, only minor differences emerge from the comparison of the trajectories obtained with frequencies of 1 and 0.1 GHz on the time scales sampled.

For both types of filament and under all conditions of field strength and frequency, the terminal segments and segment 340-KSEKLDFKDRV-350 (which is the turn of the C-shaped topology) show the largest flexibility. The presence of the oeEF enhances the intrinsic plasticity of the self-assembly without modifying the qualitative behavior of the RMSF profile along the sequence. In other words, the perturbing effect of the oeEF does not result in additional flexibility at sequence stretches that are almost rigid in the absence of a field. Thus, the oeEF seems to promote structural degradation more by an entropic effect, i.e., enhancement of intrinsic plasticity, rather than enthalpic destabilization of the more rigid segments.

There are a series of limitations in this study. First, we have used only the ordered core of the tau filament, disregarding the effect of the electric field on the disordered ″fuzzy″ coat. The lack of order means that the fuzzy coat at the N-terminal sections of tau might respond more rapidly to oscillating fields. Furthermore, it has been reported that the N-terminal segment of tau plays an important role in stabilizing the SF topology.^8^ However, in the control simulations without oeEF, the fibrillar core shows shape and behavior similar as those observed after 1 μs of simulations in which part of the N-terminal fuzzy coat was included.^25^ It is still difficult to predict the behavior of these disordered regions under the oeEF. The large size of tau (441 residues) makes it prohibitive to simulate full-length tau in a fibrillar arrangement using explicit solvent MD.

Previous literature reported a significative speed-up in the conformational sampling originated by implicit solvent.^47^ In contrast, under the influence of an electric field, a substantial increase in sampling efficiency is not observed compared to the explicit solvent runs. Nevertheless, interesting conformations have been sampled, like the noncanonical interaction between different pentameric assemblies and peptides in the assembly that lost their characteristic C-shaped arrangement (Figure 9 shows results for the runs at 0.1 GHz at 100 mV/nm or 1 GHz at 200 mV/nm). Overall, the implicit solvent results are in agreement with the explicit solvent simulations as the β-strand content is similar and the conformations are comparable. In neither case do we observe a complete degradation of the fibrillar structure along the time scale of the simulations presented here.

The time scale of the simulations is another limitation. Multiple independent runs were carried out up to 600 ns in explicit water and 110 ns in an implicit solvent. Although these time scales are clearly too short for the full degradation of the tau core filaments, they are sufficiently long for discriminating the structural stability of the two topologies and in both models of the solvent. Moreover, the submicrosecond time scales are adequate for investigating the intrinsic flexibility along the sequence of tau and the influence of the oeEF on it.

Regarding the possible (therapeutic) application of this study, it is necessary to keep in mind that noninvasive treatments based on oeEFs have the challenge of tissue impedance, which means that the perceived field in the brain is weaker than the one administered. This reduction in strength might be counterbalanced by the longer time scales over which a possible clinical treatment would be applied.

A future perspective would involve a simulation study of tau fibrils under a static electric field. Considering that tau filaments in the presented simulations respond more to the field oscillating at 0.1 GHz than at 10 GHz, it is likely that a nonoscillating field would more rapidly promote the decay of β-sheet content and/or the dissociation of the two protofilaments of SF.

## Supplementary Material



## Notes

The definition of the RMSF function, the full set of plots for the structural profiles, i.e., analogous to Figure 7 (Figures S1–S14) for the explicit solvent and to Figure 10 (Figures S15–S28) for the implicit solvent; plots of the RMSF of the tip peptides for the explicit solvent (Figure S30–S43) and implicit solvent (Figure S44–S57); the fits of the decays of the β-strand content: Figures S58–S71 for explicit solvent and Figures S72–S85 for implicit solvent runs. Finally, it presents the RMSD of the 7-EFE-9 peptide in the SF interface (Figure S86) and an assessment of the stability of the implicit solvent runs (Figures S87 and S88) (PDF)
