# Supplementary material for: Disrupting Amyloid Filaments of Tau by Means of Electric Fields
Source: J Phys Chem B. 2025 Aug 27;129(36):9124–37. doi: 10.1021/acs.jpcb.5c04393 (PMC12434675; doi:10.1021/acs.jpcb.5c04393)
Supplement: Supplementary file 1 [file jp5c04393_si_001.pdf]

Disrupting amyloid filaments of tau by means of electric fields
Supplementary Information

Pablo Andrés Vargas-Rosales, Giuseppe Giangreco, and Amedeo Caflisch*

Department of Biochemistry, University of Zurich, Zürich, Switzerland

E-mail: caflisch@bioc.uzh.ch

Phone: +41 44 635 5521

Methods

Flexibility calculation

The calculation of root mean square fluctuations (RMSF) is as follows:

```
def rmsf(traj, ts= 1, dt=10, ca=True):
    ## input
    # traj: mdtraj trajectory (package)
    # ts: timestep of the trajectory
    # dt: time to use for average
    # ca: get only RMSF of alpha carbons
    step=dt//ts
    disps_i = []
    if ca: #only calculate CA RMSF
        calphas = [ca.index for ca in traj.top.atoms_by_name("CA")]
        sliding=np.lib.stride_tricks.sliding_window_view(traj.xyz[:, calphas,:],10,axis=0)
        # 1. Calculate displacements
        disps_i = traj.xyz[:,calphas,:]-np.concatenate(([traj.xyz[:i, calphas,:].mean(axis=0) for i in range(1,step)],sliding.mean(axis=3)))
        # 2. Square and convert to A
        sq_disps = np.square(np.sum(disps_i, axis=2)*10)
        # 3. Square root
        rmsf = np.sqrt(np.mean(sq_disps, axis=0))
    else: #calculate heavy atom RMSF
        #heavy_atoms = [i for i in traj.top.select_atom_indices("heavy")]
        heavy_atoms = traj.top.select("mass > 2")
        heavy_residues = [traj.top.atom(i).residue.index for i in heavy_atoms]        sliding=np.lib.stride_tricks.sliding_window_view(traj.xyz[:,heavy_atoms,:],10,axis=0)
        # 1. Calculate displacements
        disps_i = traj.xyz[:,heavy_atoms,:]-np.concatenate(([traj.xyz[:i, heavy_atoms,:].mean(axis=0) for i in range(1,step)],sliding.mean(axis
```

```
=3)))
        # 2. Square and convert to A
        sq_disps = np.square(np.sum(disps_i, axis=2)*10)
        # 3. Square root
        rmsf = np.sqrt(np.mean(sq_disps, axis=0))
        # 4. Aggregate in residues
        rmsf = [np.mean(i) for i in np.split(rmsf, np.unique(
heavy_residues, return_index = True)[1])[1:]]
    # Output
    ## array in shape of residues
    return rmsf
```

It is important to note, that unlike traditional RMSF, we align the system on the central chain of each pentameric assembly and calculate the RMSF on each peptide of the assembly.

Results

Here we report additional data from the simulations. Due to the large amount of conditions tested, we prefer not to show them in the main text.

Structural profiles of the systems

In the following we present the analysis of the structural stability of the decameric assemblies. We consider contacts and fluctuation. Three types of contact were considered: interpeptide, referring to residues within 0.8 nm from other residues in peptides within the same protofilament; intrapeptide, meaning the contacts (distance ¡ 1.25 nm) between residues of the same peptide chain; interfibrillar, monitoring the interaction between residues located in different protofilaments which have a distance smaller than 1 nm.

Explicit solvent simulations

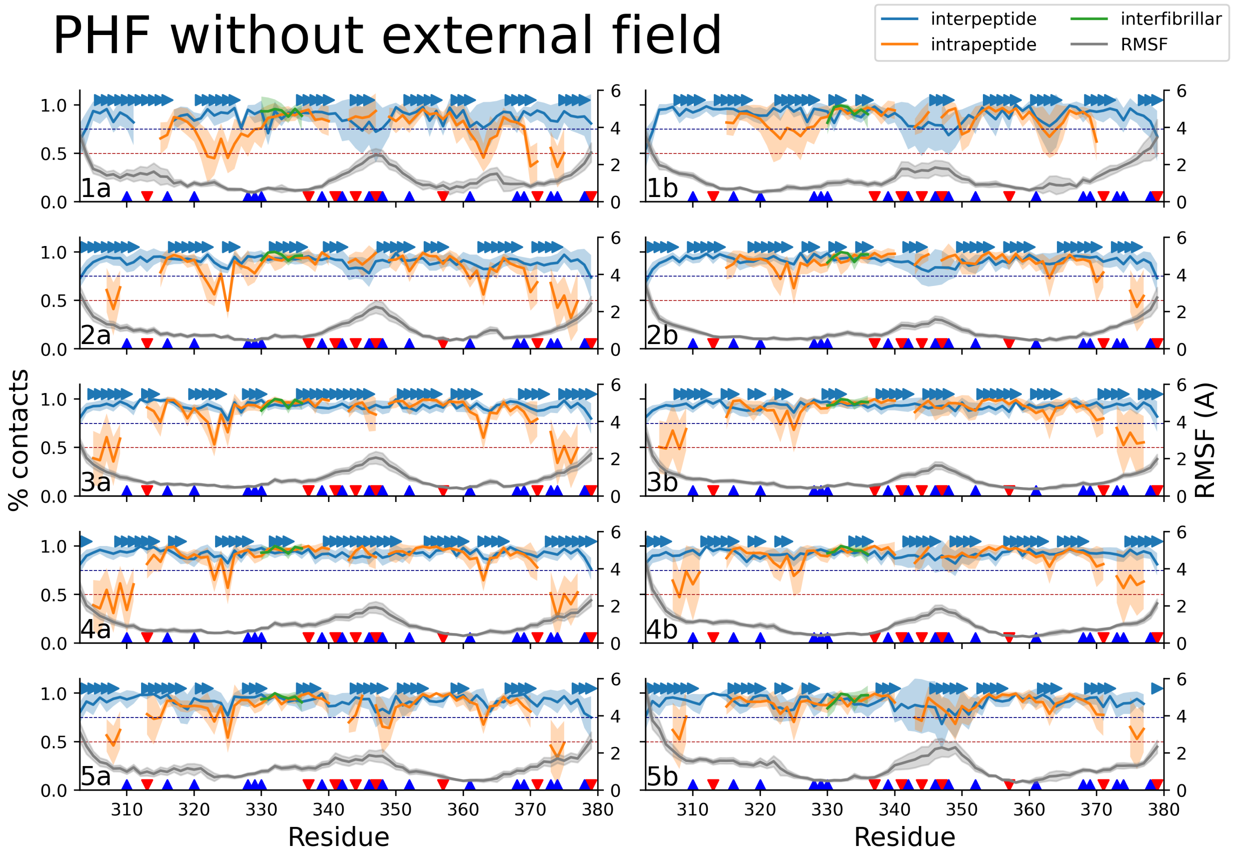

Figure S 1: Structural stability of PHF protofibril at peptide and residue level, in absence of oeEF. The percentage of contacts (left y-axis) and structural flexibility (right y-axis) for each peptide of the protofibril are shown. Three different types of contact are shown: interpeptide, referring to contacts with residues from other adjacent peptides within the same protofilament; intrapeptide, meaning the contacts between residues of the same peptide chain; interfibrillar, monitoring the interaction between the two protofilaments. The root mean square fluctuation is shown as a gray trace. For each peptide, the frames are aligned to the central (i.e. 3a or 3b) peptide of each protofilament, and the fluctuations calculated averaging over blocks of 10 ns. The beta strand regions for each peptide are shown in the top of each peptide as blue horizontal arrows, while the presence of positively and negatively charged residues are marked by vertical blue and red triangles in the bottom respectively.

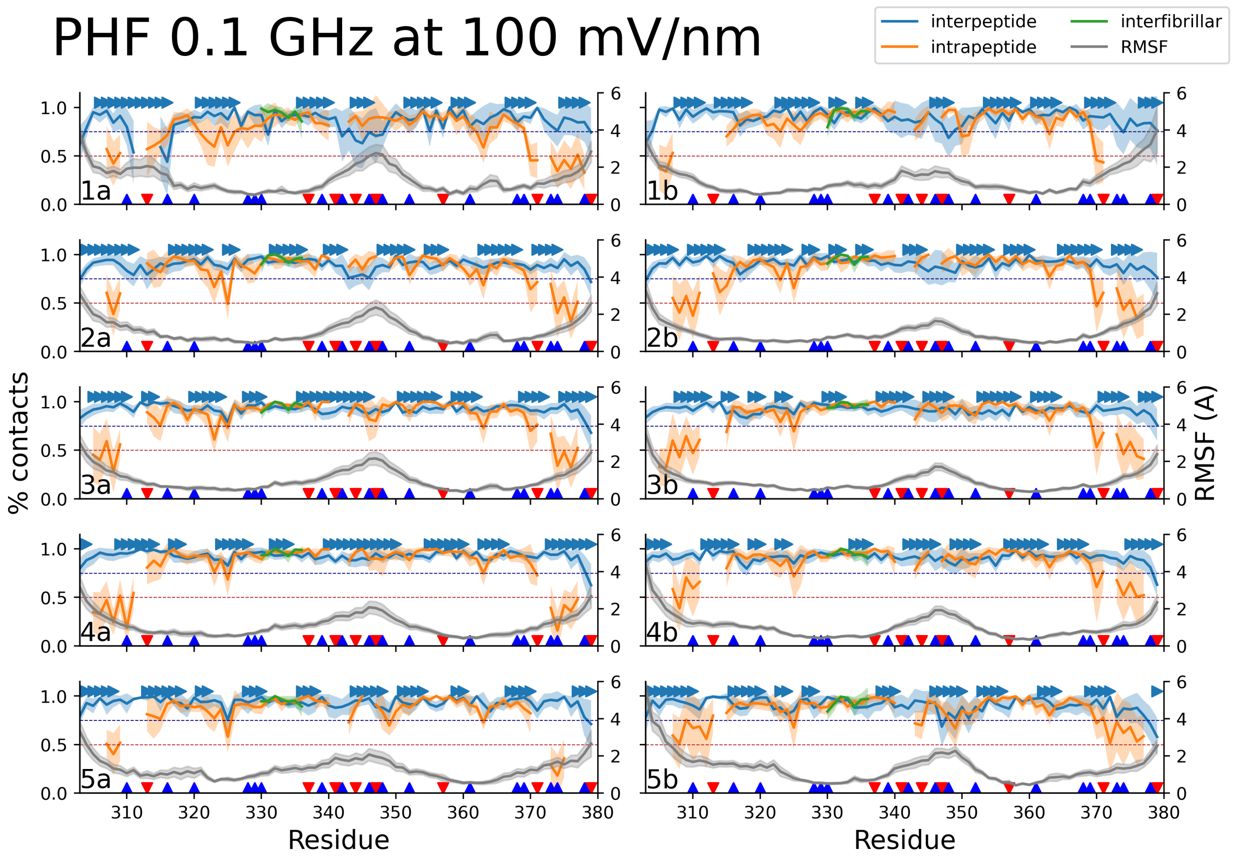

Figure S 2: Structural stability of PHF protofibril at peptide and residue level, with 100 mV/nm oeEF oscillating at 0.1 GHz. Same representation as Figure S1

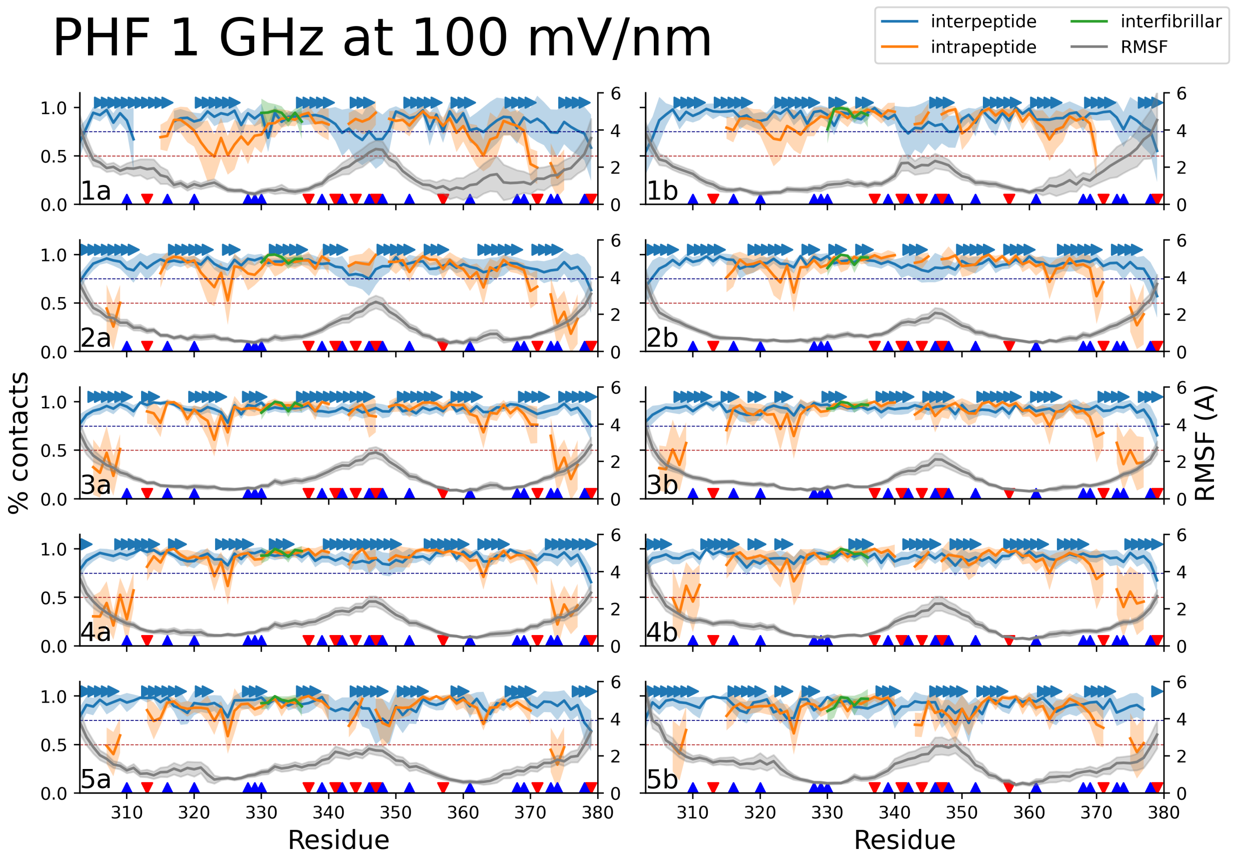

Figure S 3: Structural stability of PHF protofibril at peptide and residue level, with 100 mV/nm oeEF oscillating at 1 GHz. Same representation as Figure S1

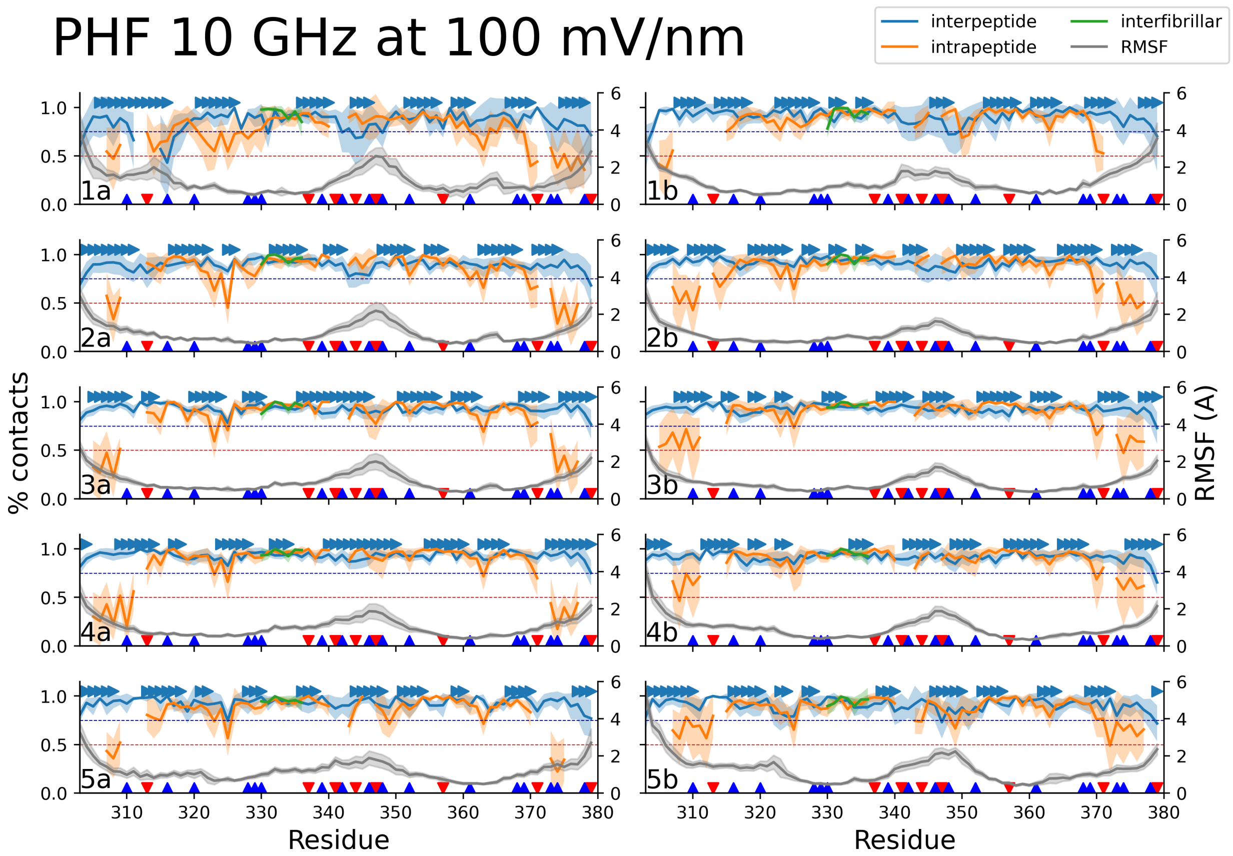

Figure S 4: Structural stability of PHF protofibril at peptide and residue level, with 100 mV/nm oeEF oscillating at 10 GHz. Same representation as Figure S1

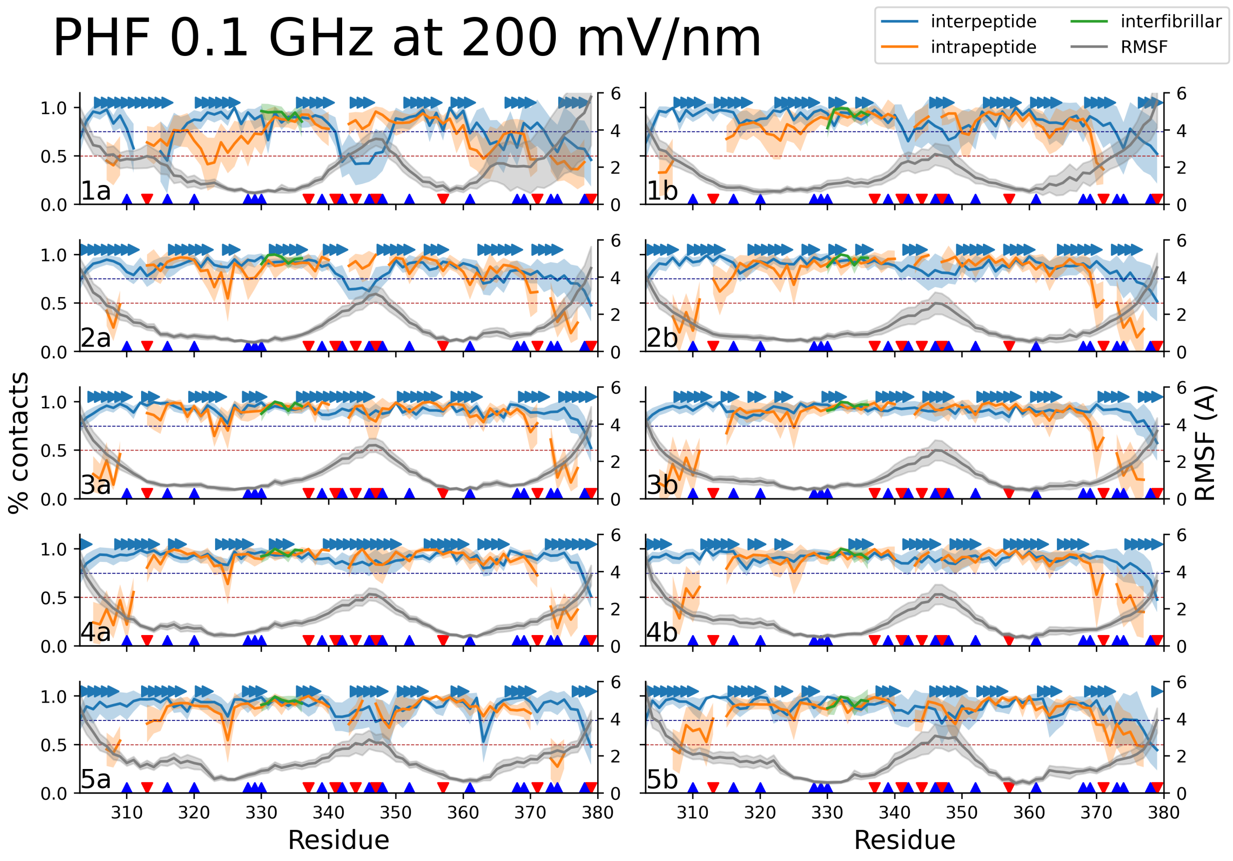

Figure S 5: Structural stability of PHF protofibril at peptide and residue level, with 200 mV/nm oeEF oscillating at 0.1 GHz. Same representation as Figure S1

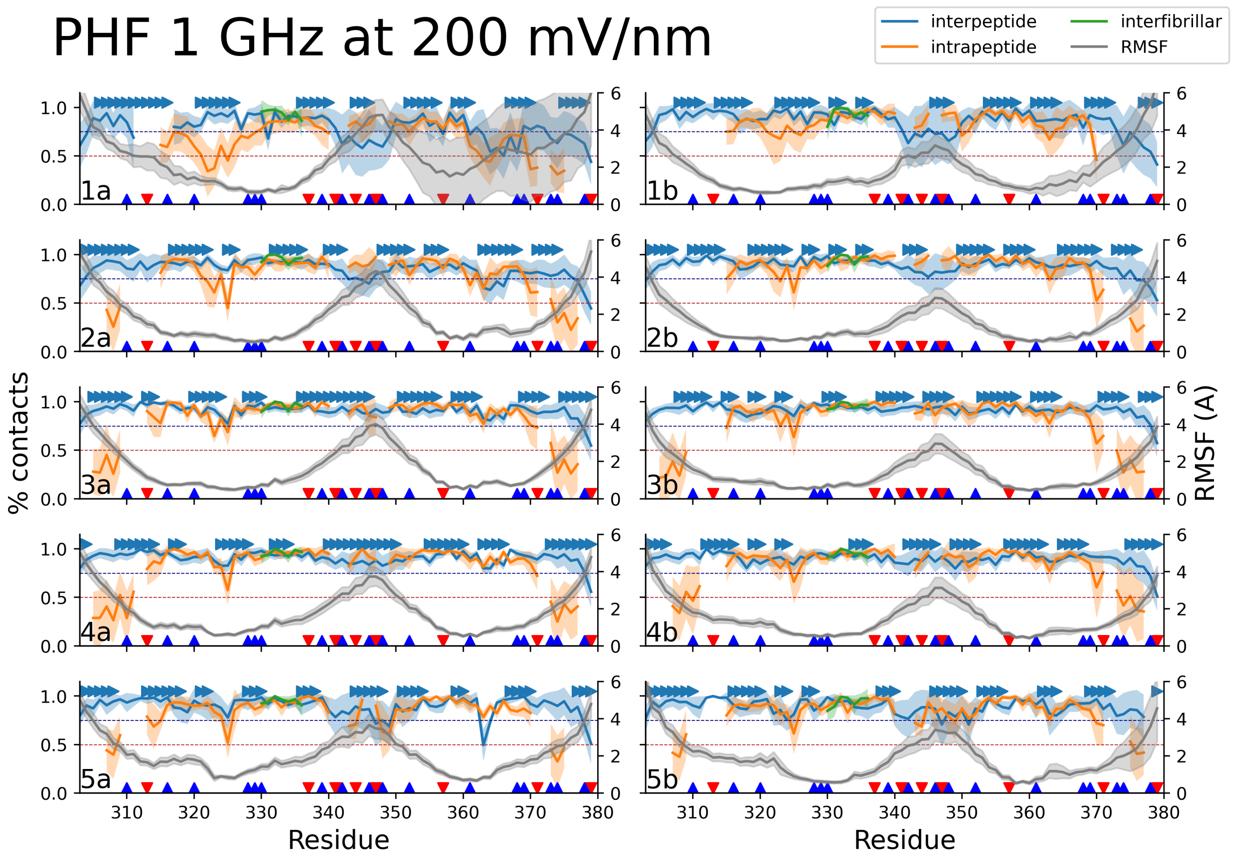

Figure S 6: Structural stability of PHF protofibril at peptide and residue level, with 200 mV/nm oeEF oscillating at 1 GHz. Same representation as Figure S1

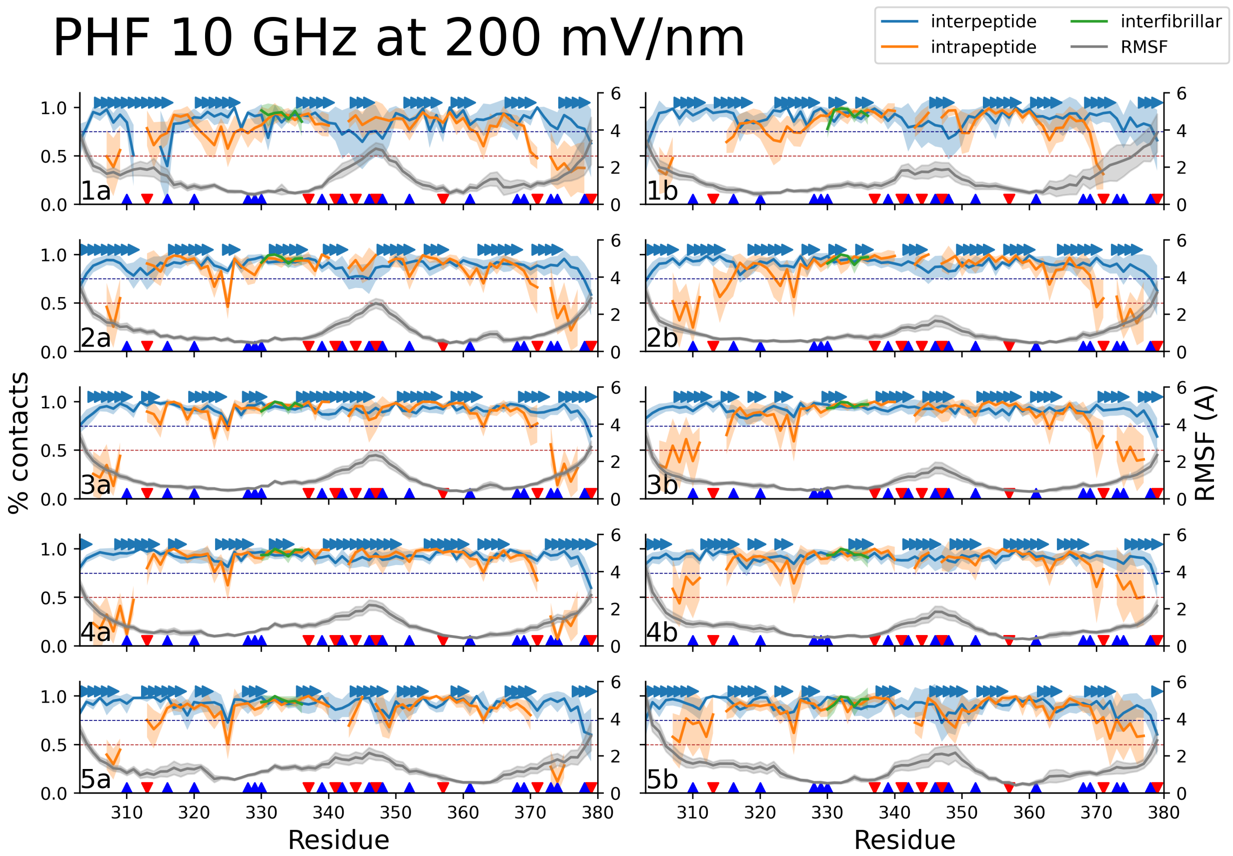

Figure S 7: Structural stability of PHF protofibril at peptide and residue level, with 200 mV/nm oeEF oscillating at 10 GHz. Same representation as Figure S1

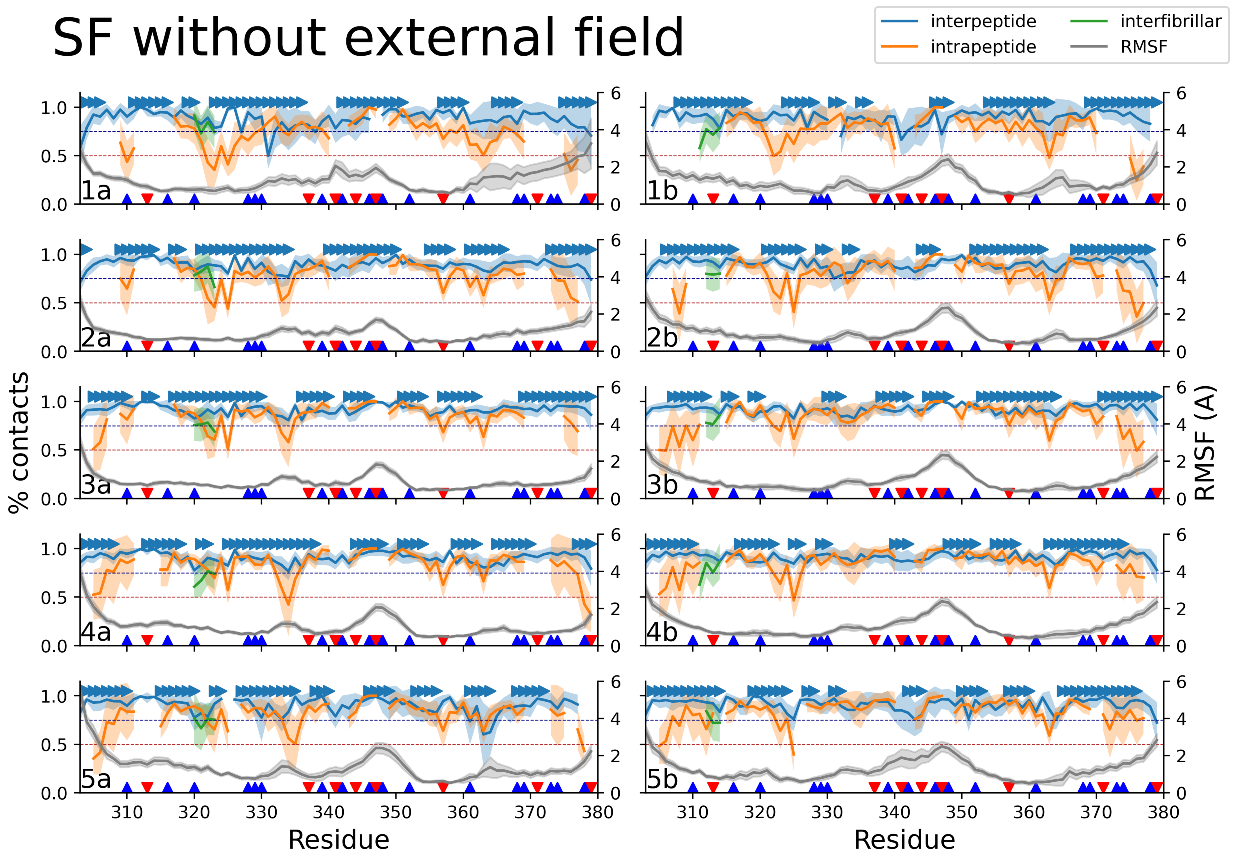

Figure S 8: Structural stability of SF protofibril at peptide and residue level, in absence of oeEF. Same representation as Figure S1

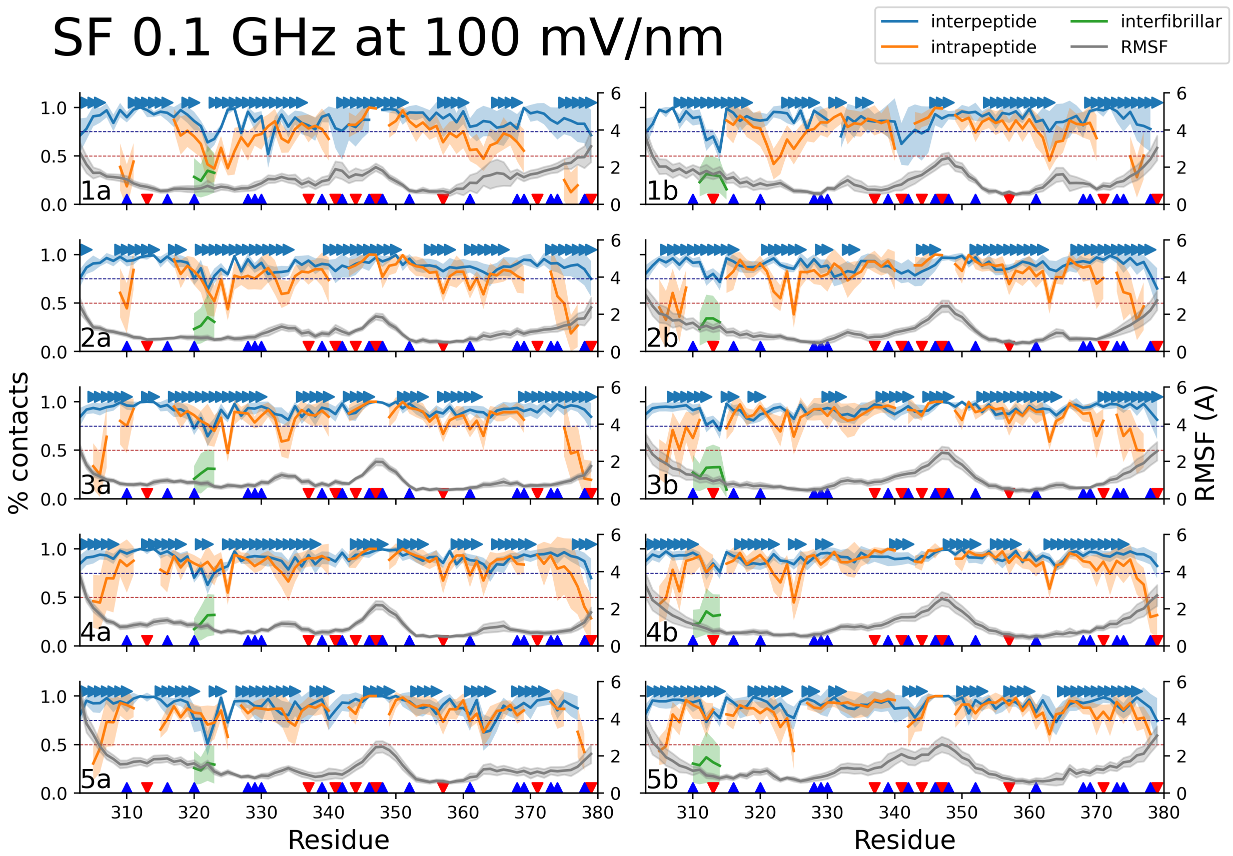

Figure S 9: Structural stability of SF protofibril at peptide and residue level, with 100 mV/nm oeEF oscillating at 0.1 GHz. Same representation as Figure S1

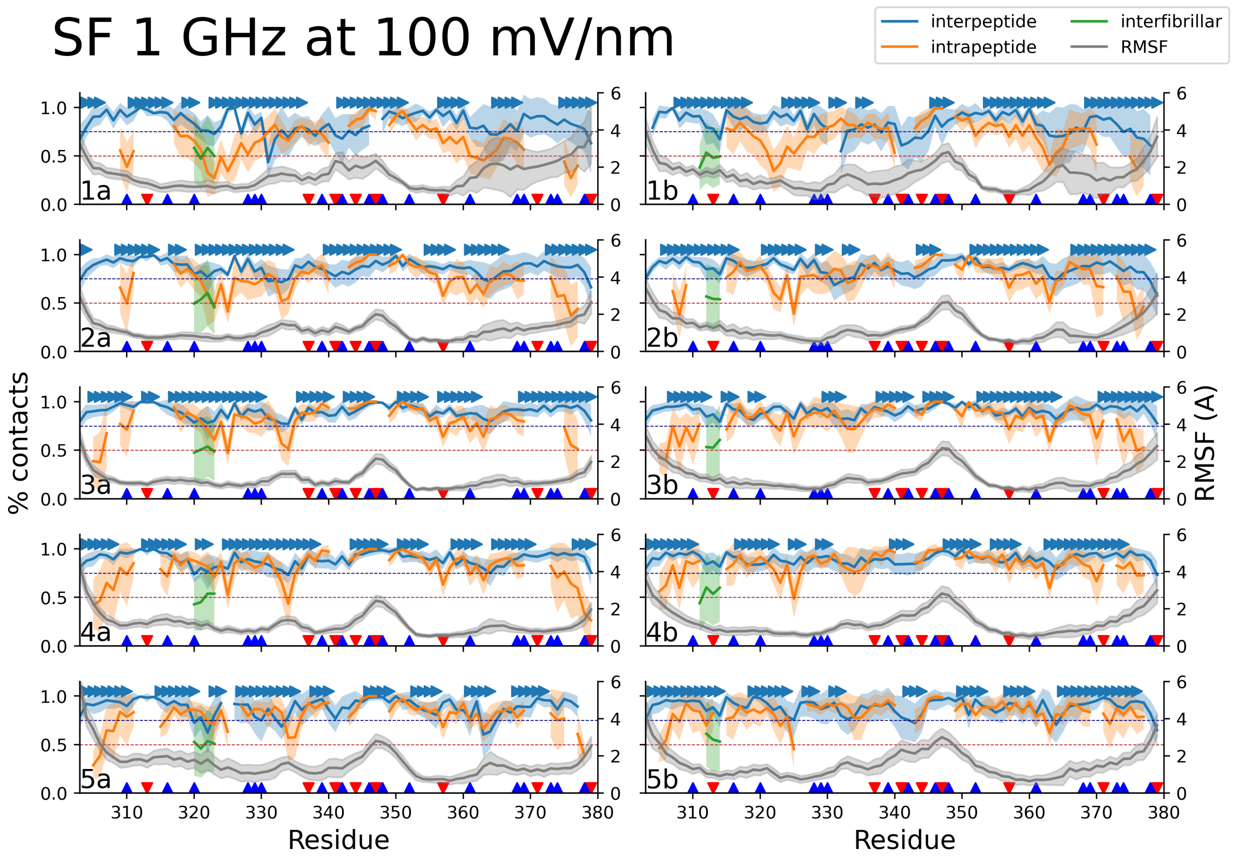

Figure S 10: Structural stability of SF protofibril at peptide and residue level, with 100 mV/nm oeEF oscillating at 1 GHz. Same representation as Figure S1

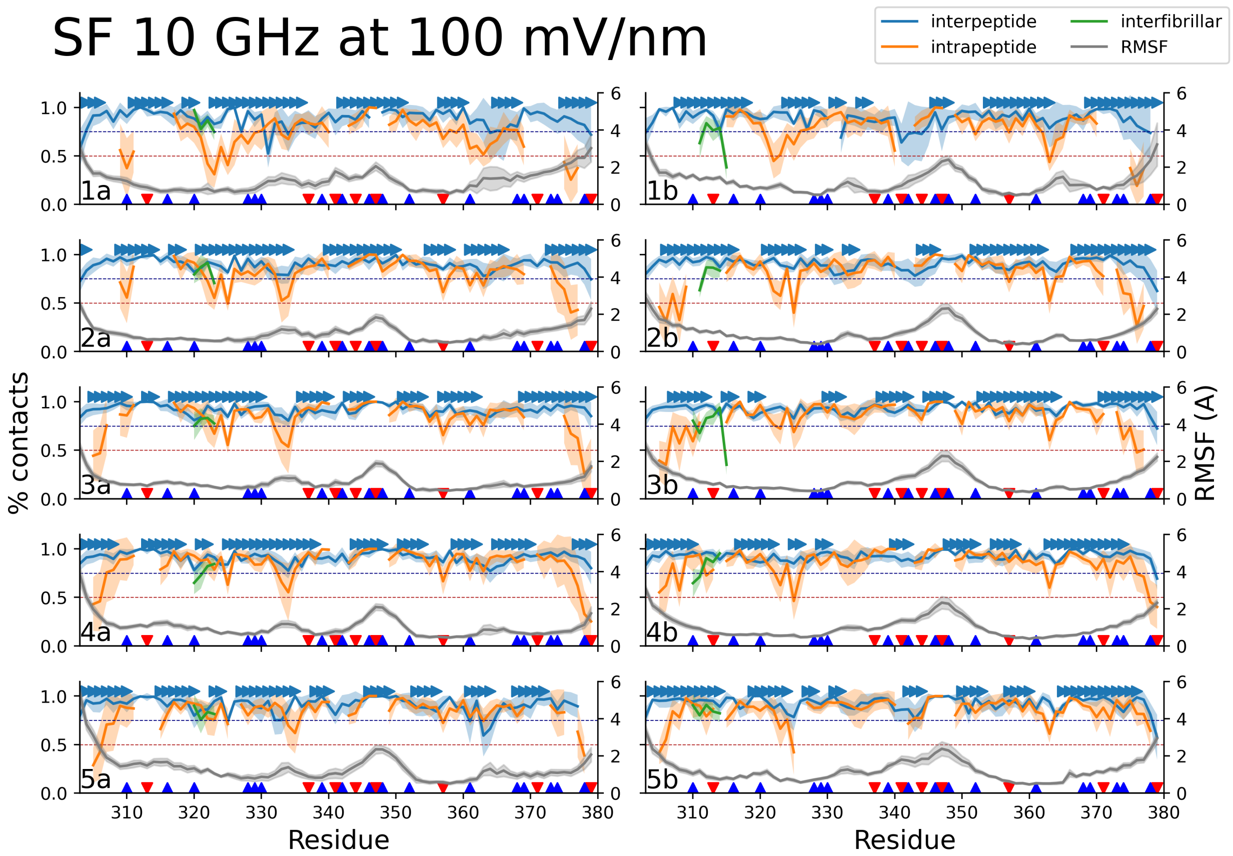

Figure S 11: Structural stability of SF protofibril at peptide and residue level, with 100 mV/nm oeEF oscillating at 10 GHz. Same representation as Figure S1

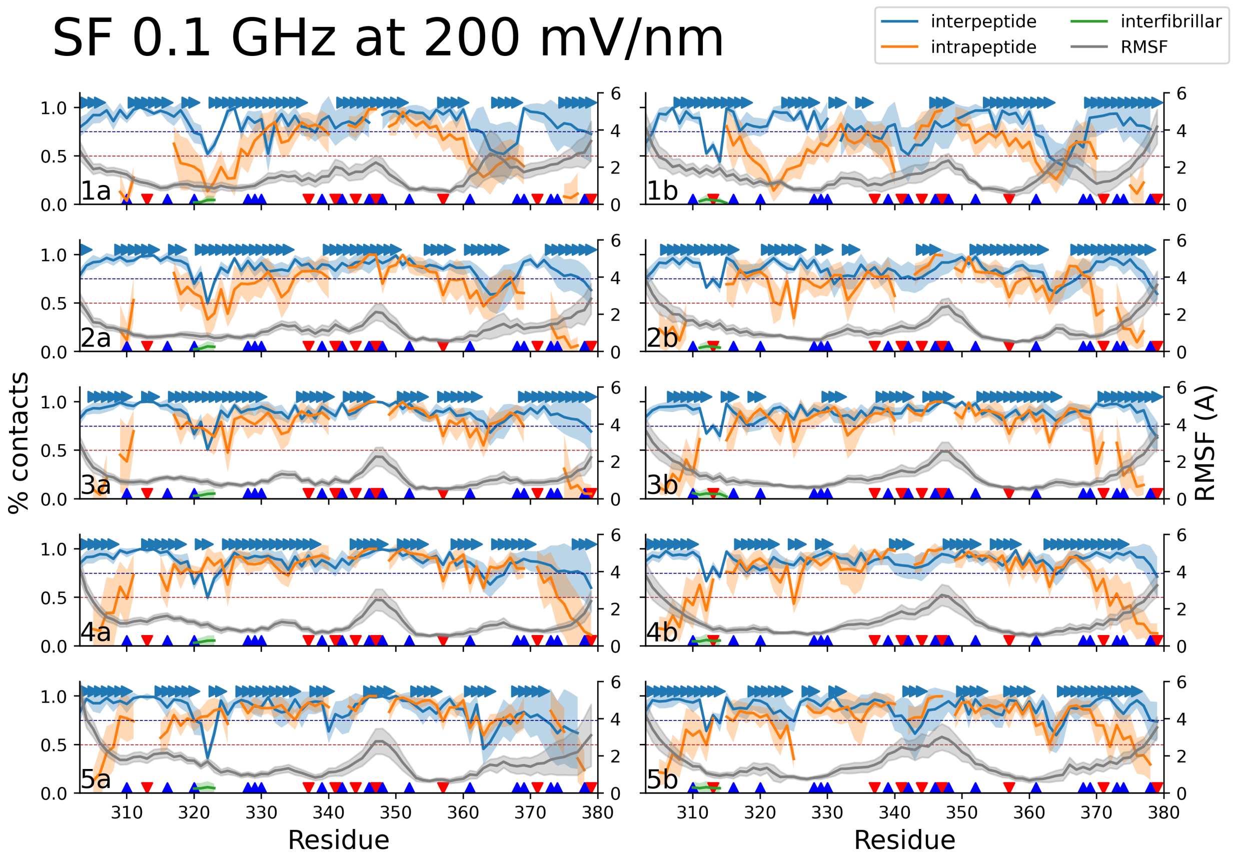

Figure S 12: Structural stability of SF protofibril at peptide and residue level, with 200 mV/nm oeEF oscillating at 0.1 GHz. Same representation as Figure S1

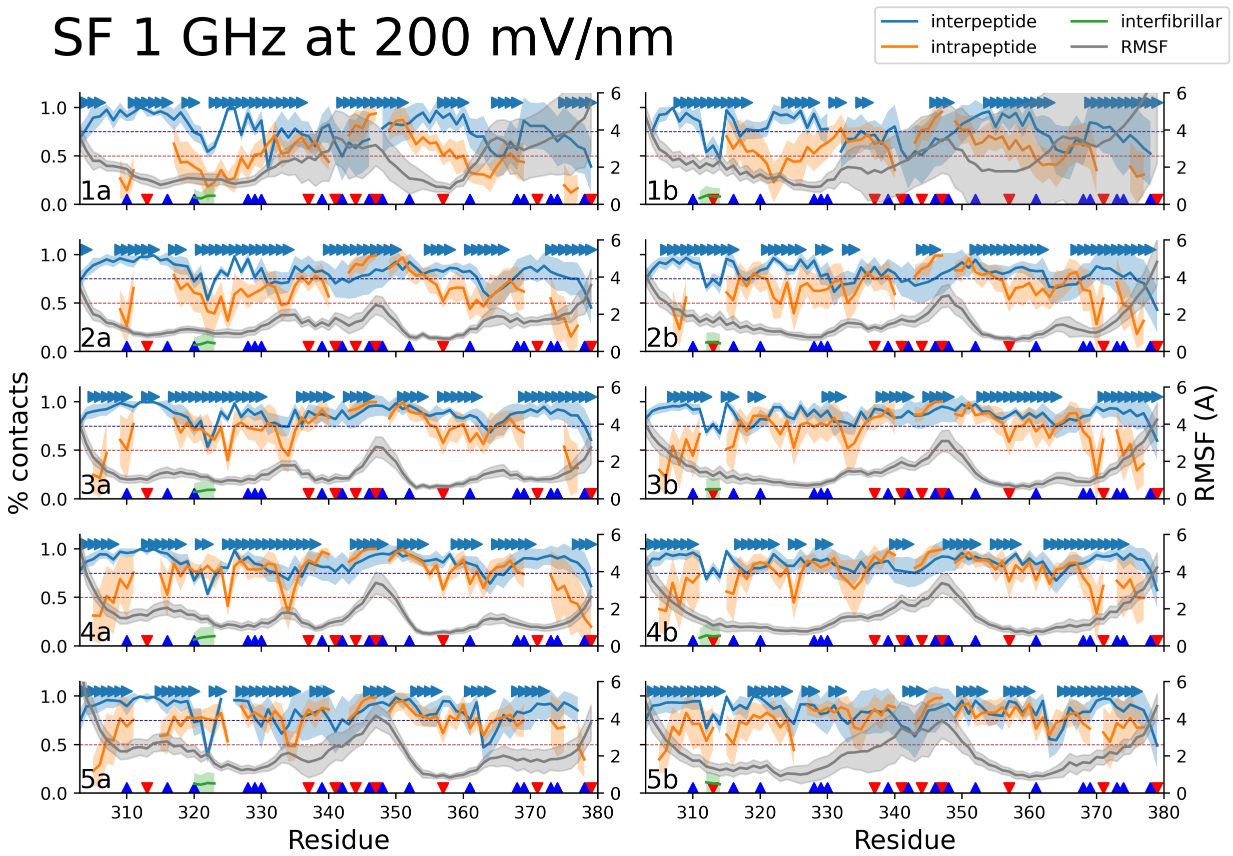

Figure S 13: Structural stability of SF protofibril at peptide and residue level, with 100 mV/nm oeEF oscillating at 1 GHz. Same representation as Figure S1

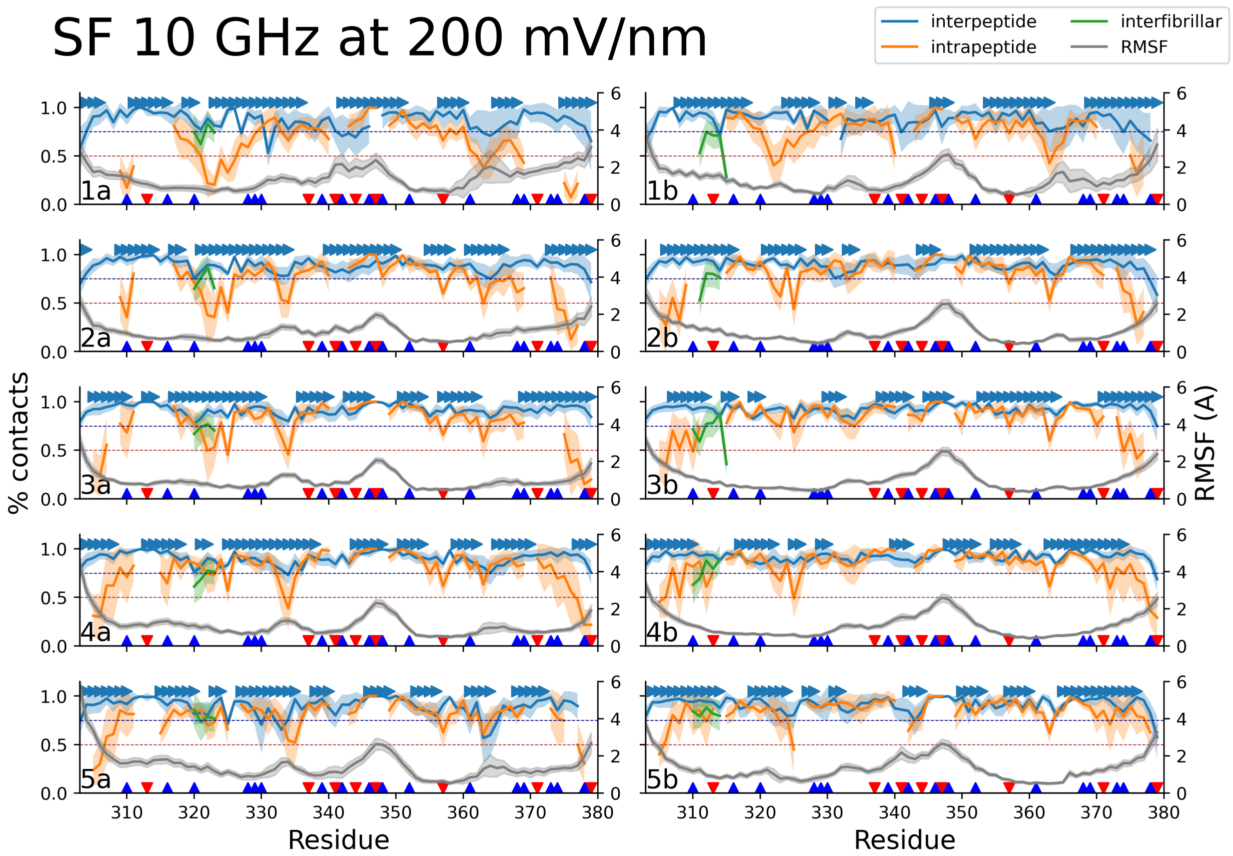

Figure S 14: Structural stability of SF protofibril at peptide and residue and residue level, with 100 mV/nm oeEF oscillating at 10 GHz. Same representation as Figure S1

Implicit solvent simulations

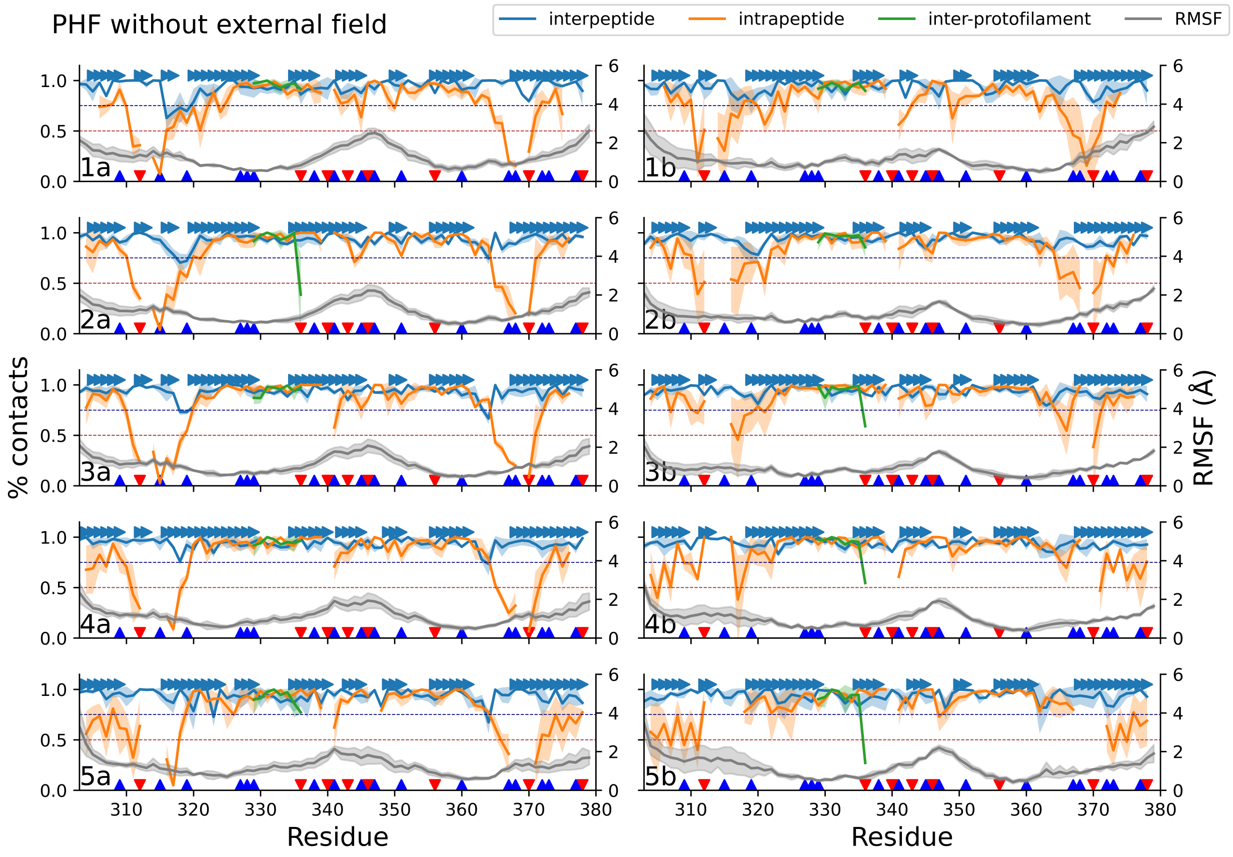

Figure S 15: Structural stability of PHF protofibril at peptide and residue level in implicit solvent, in absence of oeEF. Same representation as Figure S1

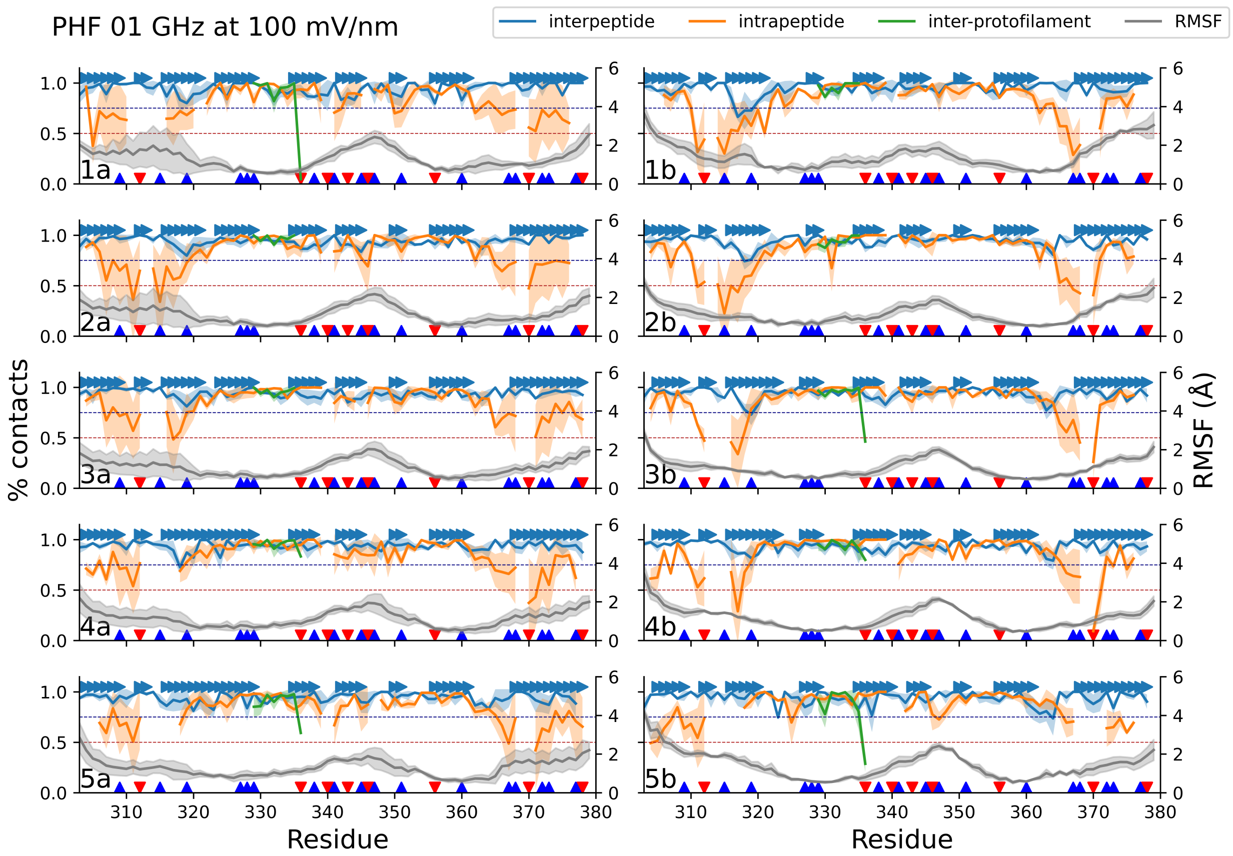

Figure S 16: Structural stability of PHF protofibril at peptide and residue level in implicit solvent, with 100 mV/nm oeEF oscillating at 0.1 GHz. Same representation as Figure S1

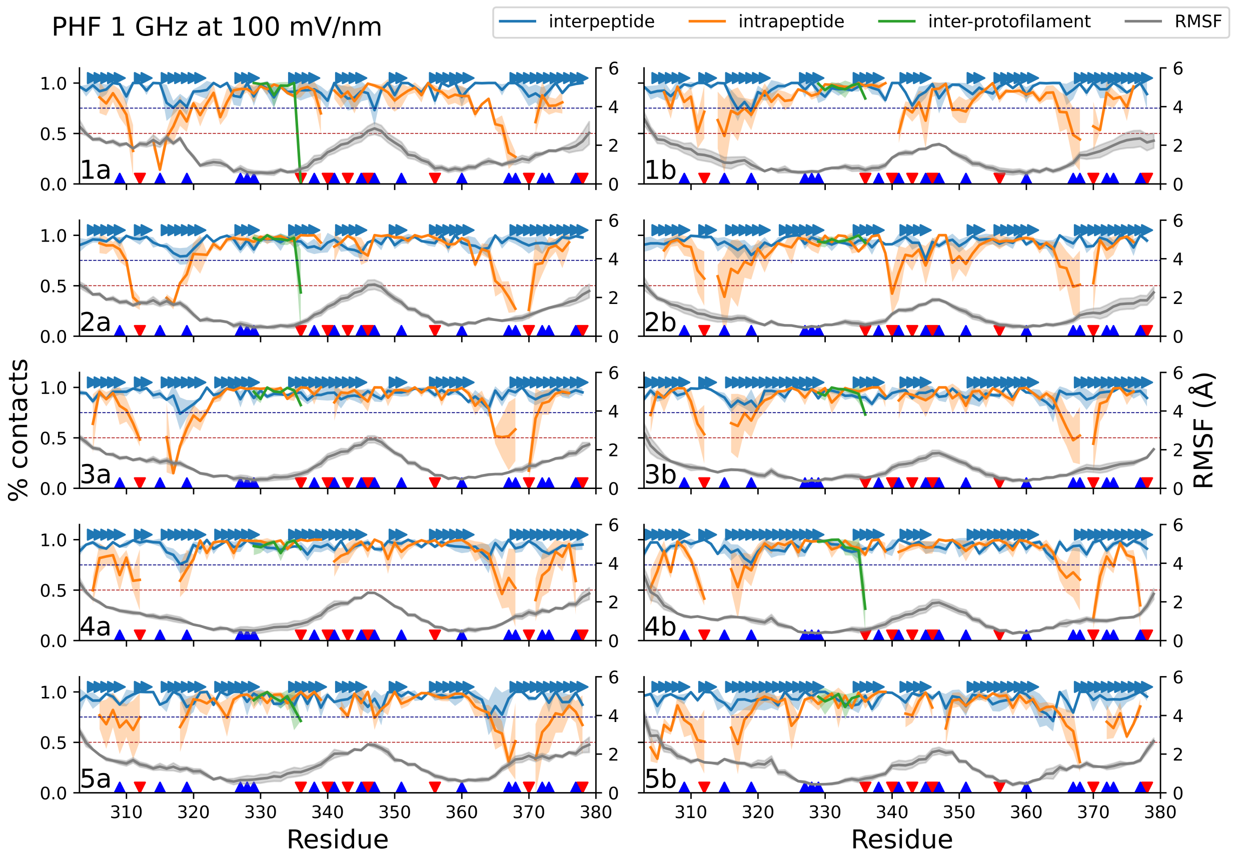

Figure S 17: Structural stability of PHF protofibril at peptide and residue level in implicit solvent, with 100 mV/nm oeEF oscillating at 1 GHz. Same representation as Figure S1

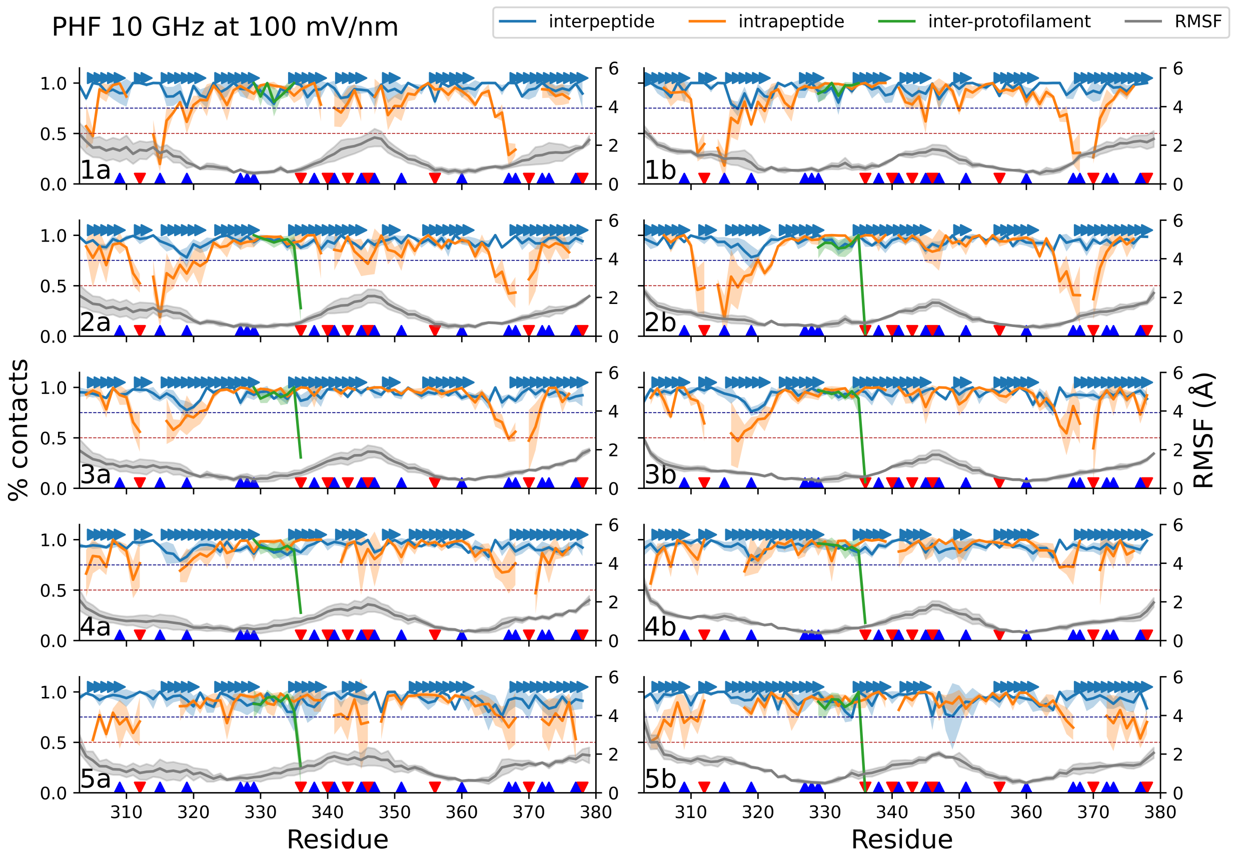

Figure S 18: Structural stability of PHF protofibril at peptide and residue level in implicit solvent, with 100 mV/nm oeEF oscillating at 10 GHz. Same representation as Figure S1

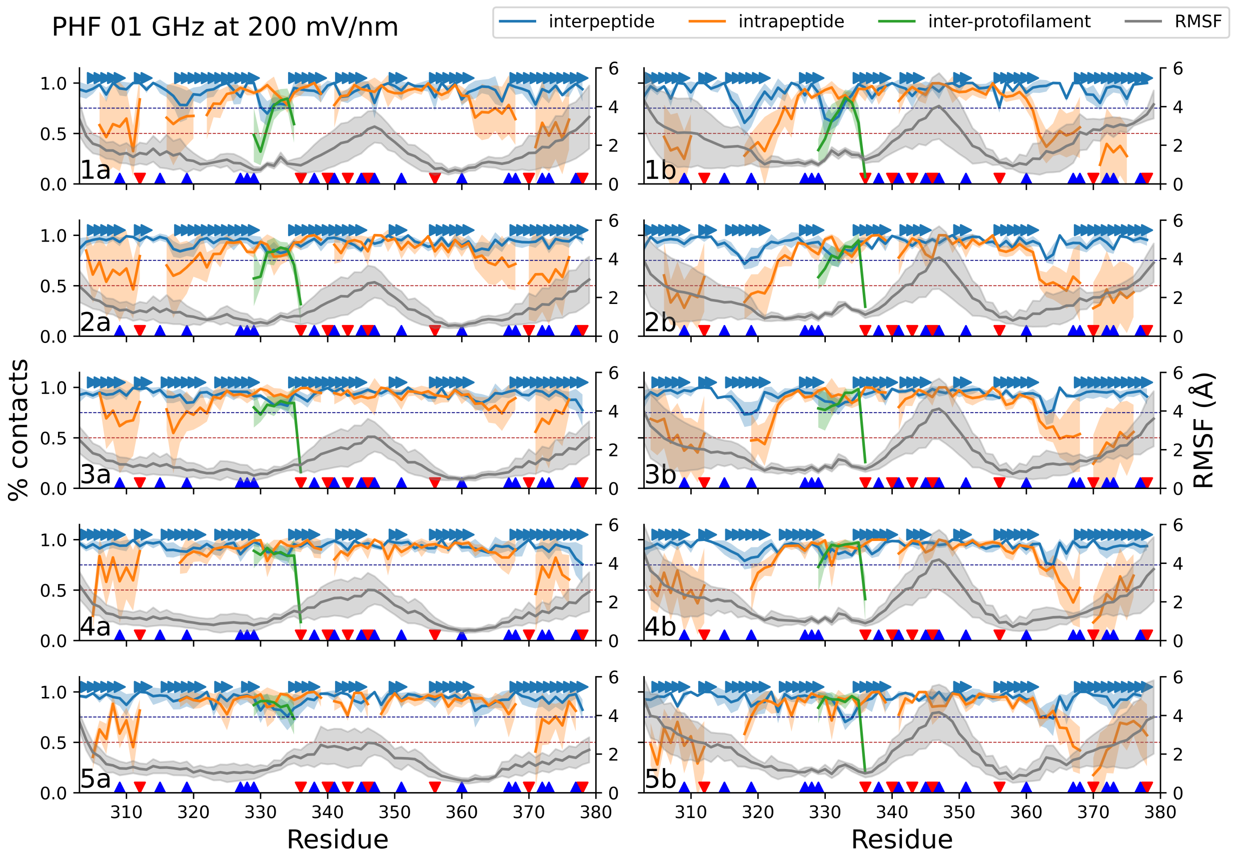

Figure S 19: Structural stability of PHF protofibril at peptide and residue level in implicit solvent, with 200 mV/nm oeEF oscillating at 0.1 GHz. Same representation as Figure S1

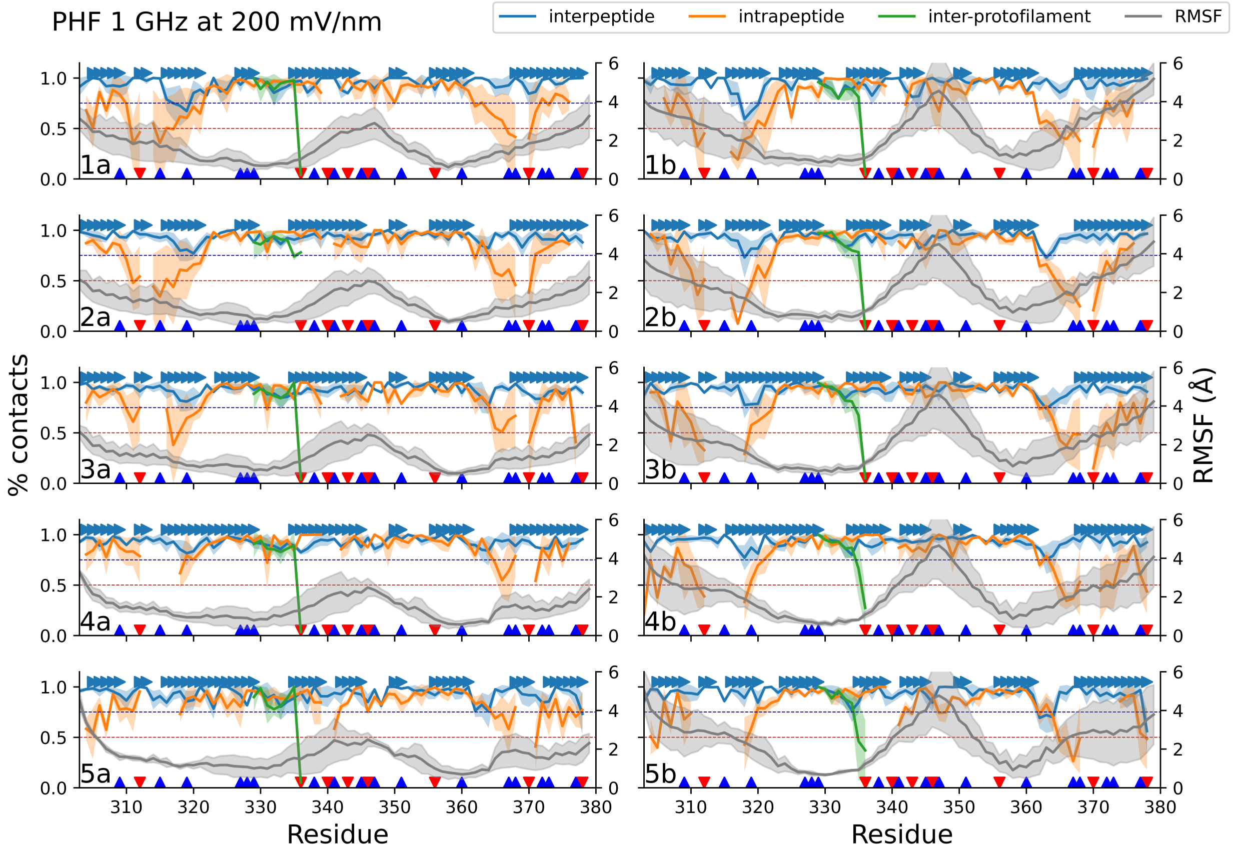

Figure S 20: Structural stability of PHF protofibril at peptide and residue level in implicit solvent, with 200 mV/nm oeEF oscillating at 1 GHz. Same representation as Figure S1

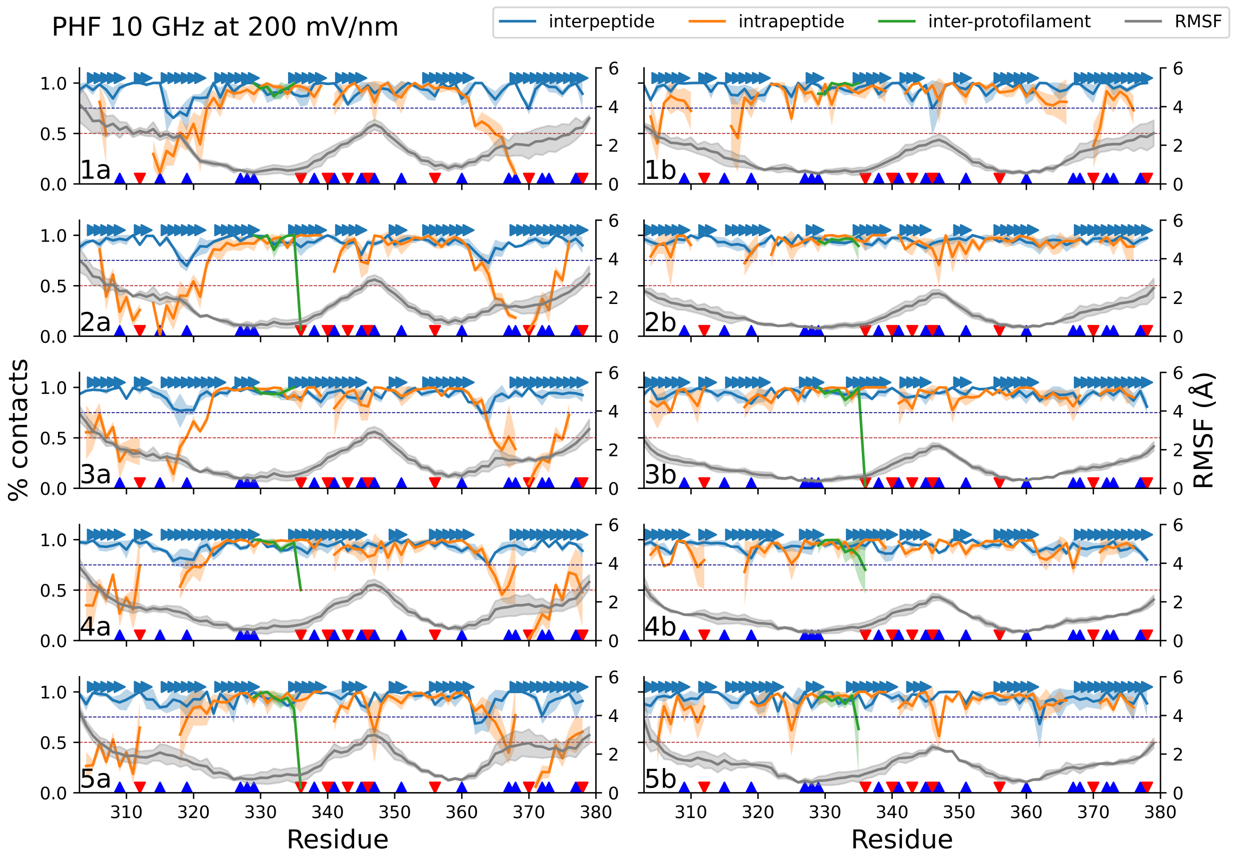

Figure S 21: Structural stability of PHF protofibril at peptide and residue level in implicit solvent, with 200 mV/nm oeEF oscillating at 10 GHz. Same representation as Figure S1

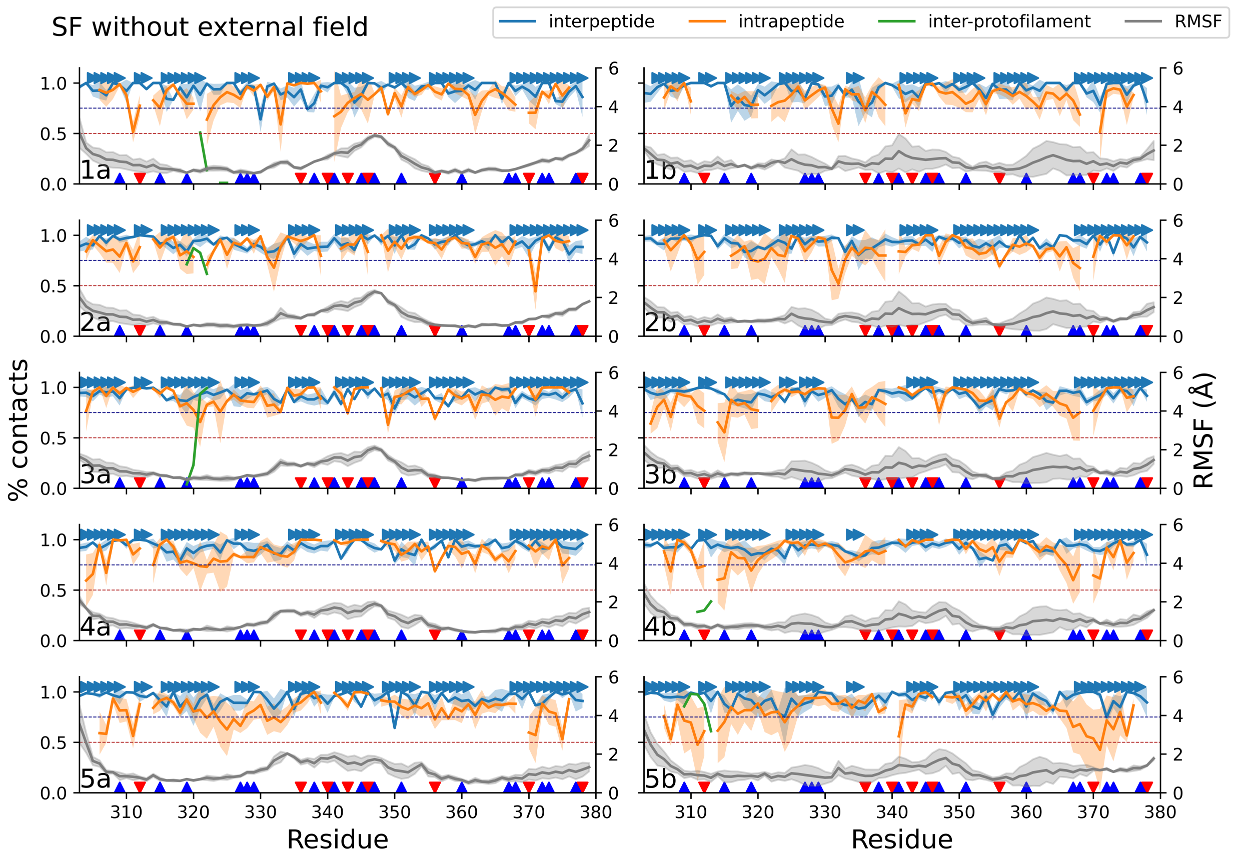

Figure S 22: Structural stability of SF protofibril at peptide and residue level in implicit solvent, in absence of oeEF. Same representation as Figure S1

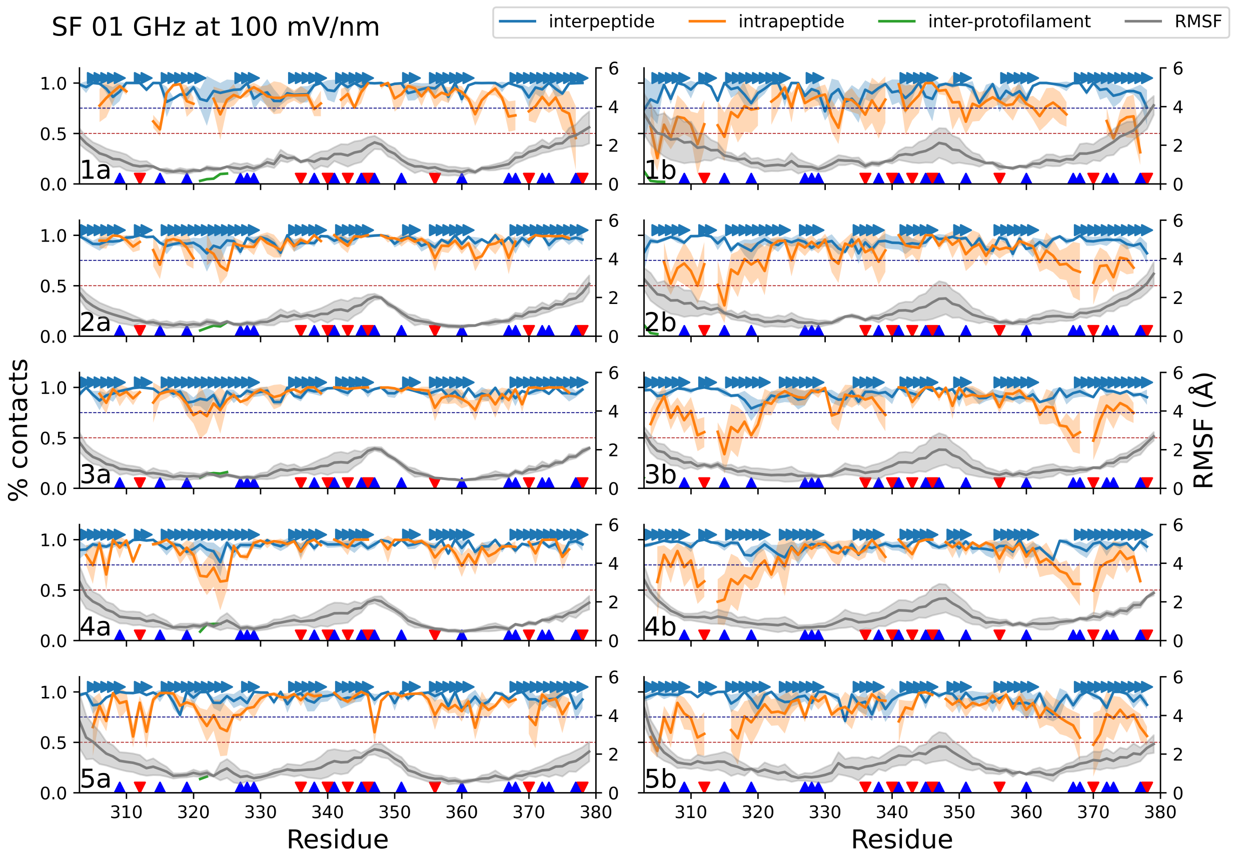

Figure S 23: Structural stability of SF protofibril at peptide and residue level in implicit solvent, with 100 mV/nm oeEF oscillating at 0.1 GHz. Same representation as Figure S1

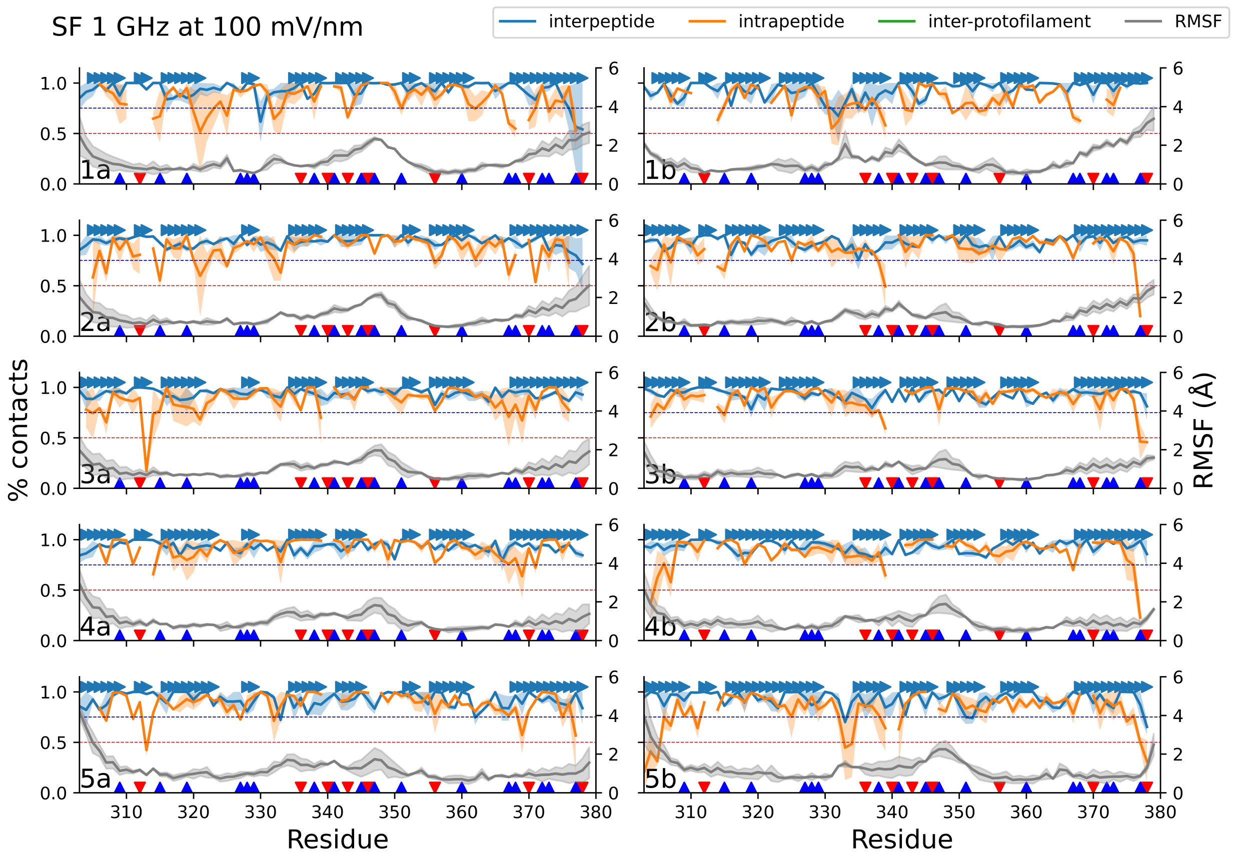

Figure S 24: Structural stability of SF protofibril at peptide and residue level in implicit solvent, with 100 mV/nm oeEF oscillating at 1 GHz. Same representation as Figure S1

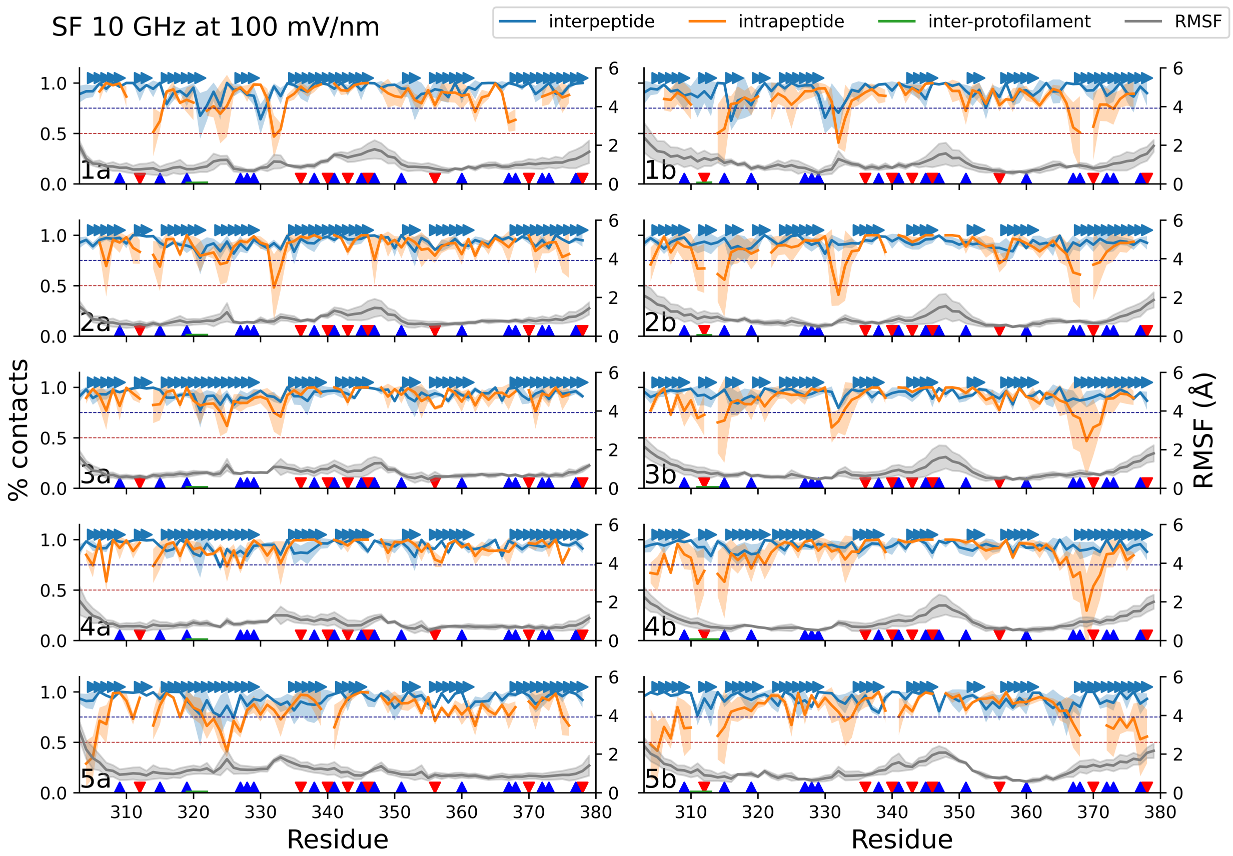

Figure S 25: Structural stability of SF protofibril at peptide and residue level in implicit solvent, with 100 mV/nm oeEF oscillating at 10 GHz. Same representation as Figure S1

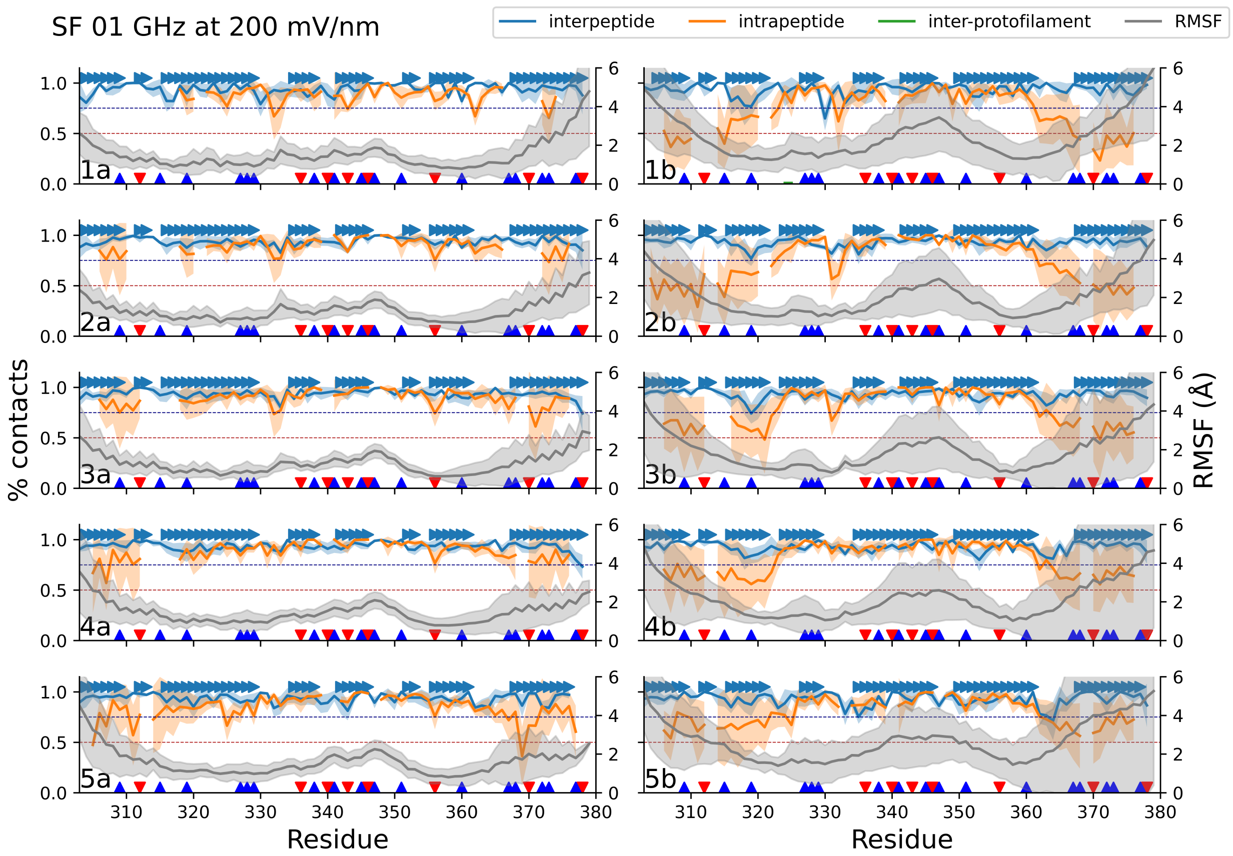

Figure S 26: Structural stability of SF protofibril at peptide and residue level in implicit solvent, with 200 mV/nm oeEF oscillating at 0.1 GHz. Same representation as Figure S1

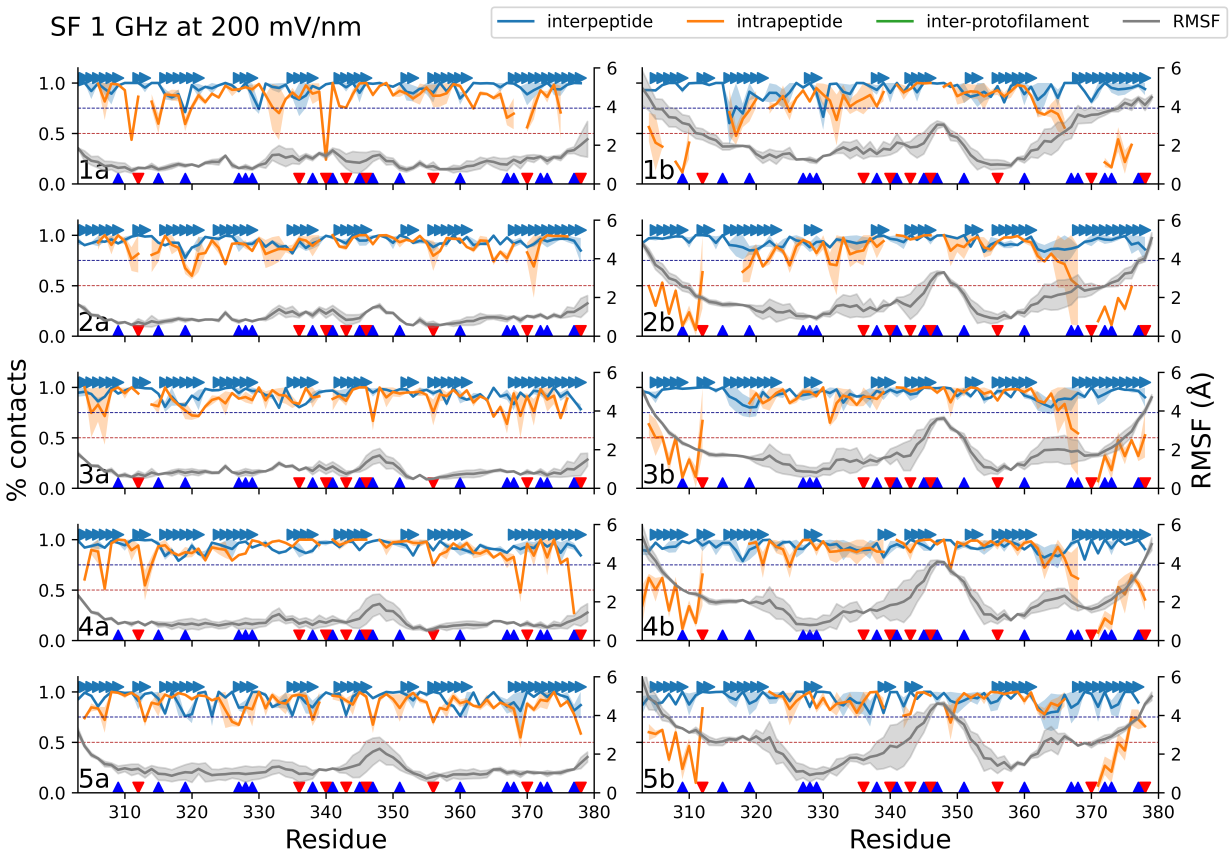

Figure S 27: Structural stability of SF protofibril at peptide and residue level in implicit solvent, with 100 mV/nm oeEF oscillating at 1 GHz. Same representation as Figure S1

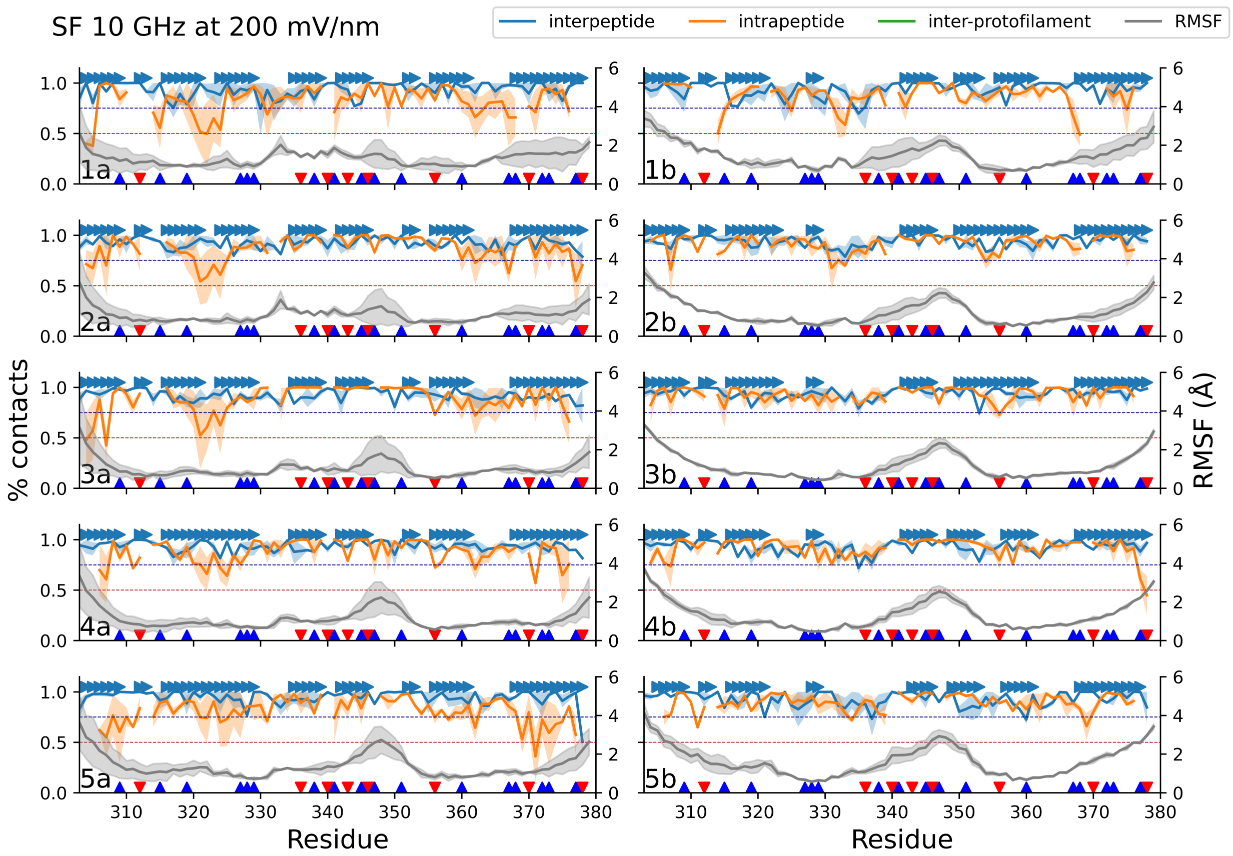

Figure S 28: Structural stability of SF protofibril at peptide and residue level in implicit solvent, with 100 mV/nm oeEF oscillating at 10 GHz. Same representation as Figure S1

Disorder at the tips of the protofibril

We show here the behavior of the tip peptides: 1a, 1b, 2a, 2b, 4a, 4b, 5a, and 5b.

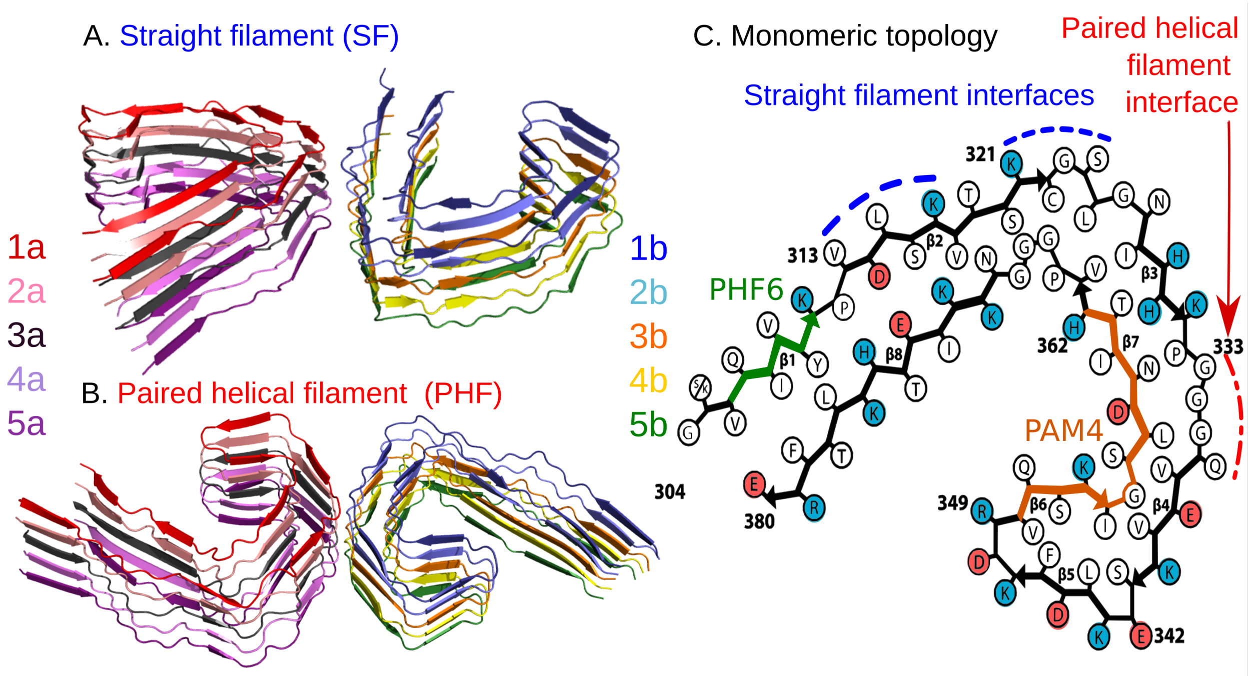

Figure S 29: Topology of tau fibrils.

Explicit solvent simulations

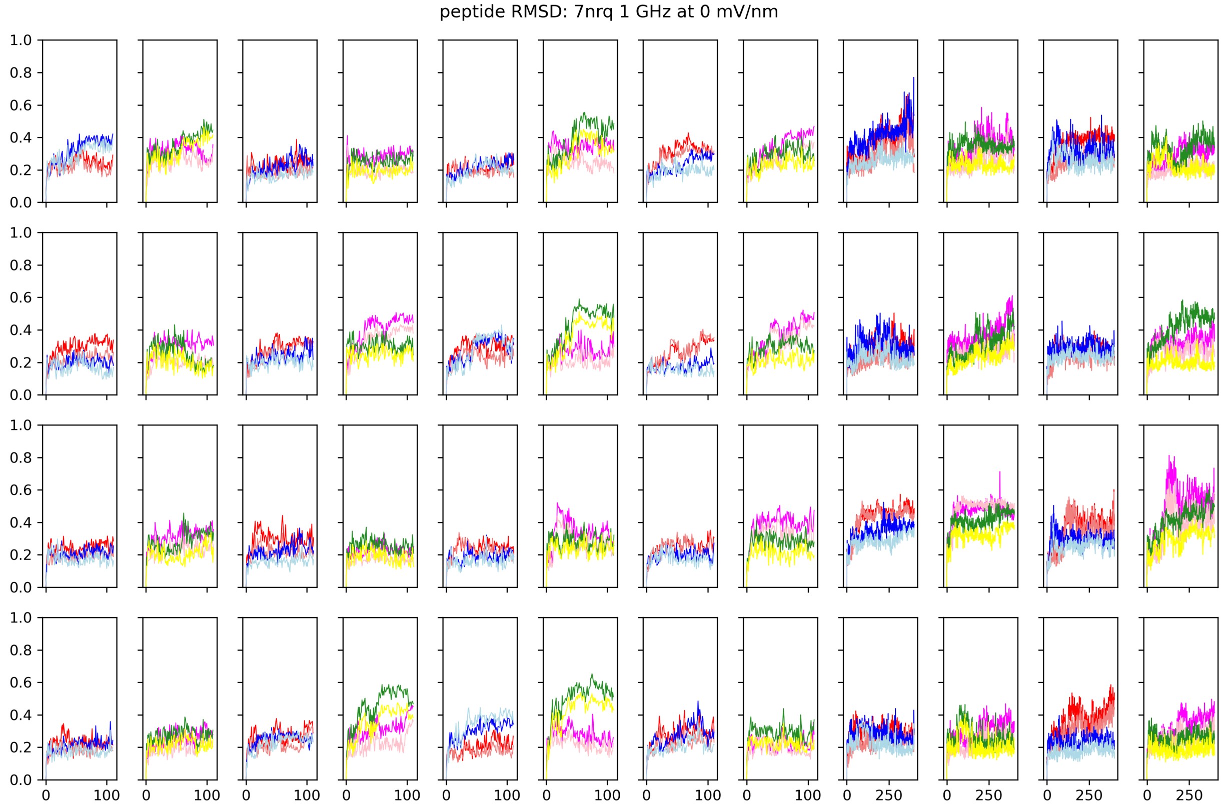

Figure S 30: Time-resolved root mean square deviation of tip peptides of PHF without oeEF. The RMSD of the peptides at the tips are shown. The coloring follows the one of Figure S29. Every two columns correspond to the same trajectory.

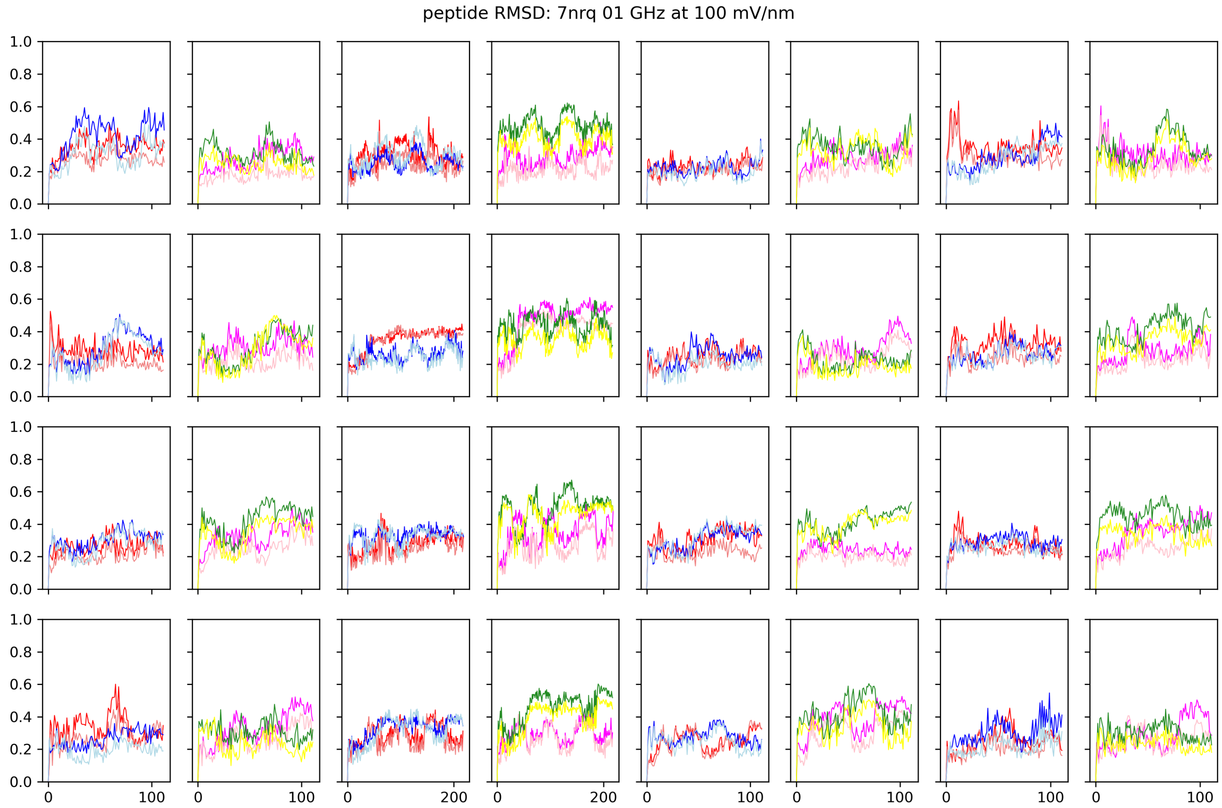

Figure S 31: Time-resolved root mean square deviation of tip peptides of PHF with a 100 mV/nm oeEF oscillating at 0.1 GHz. Same visualization as Figure S30

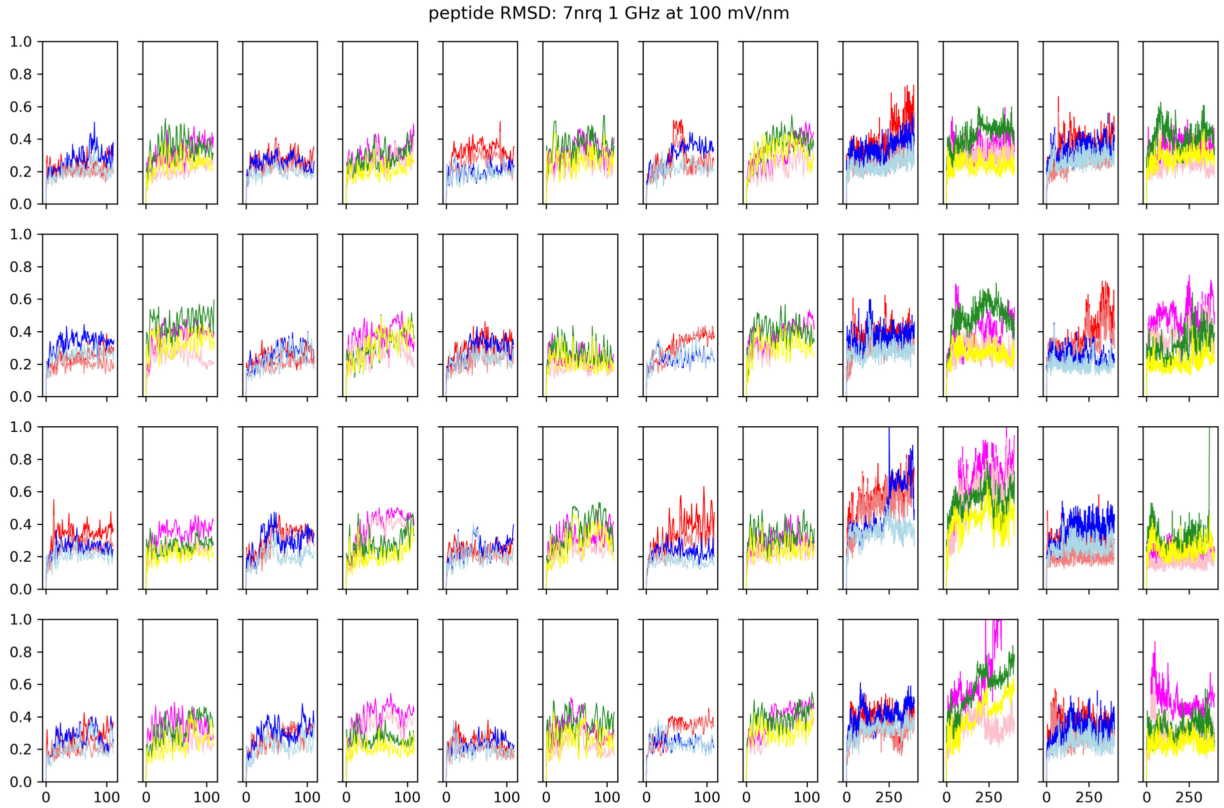

Figure S 32: Time-resolved root mean square deviation of tip peptides of PHF with a 100 mV/nm oeEF oscillating at 1 GHz. Same visualization as Figure S30

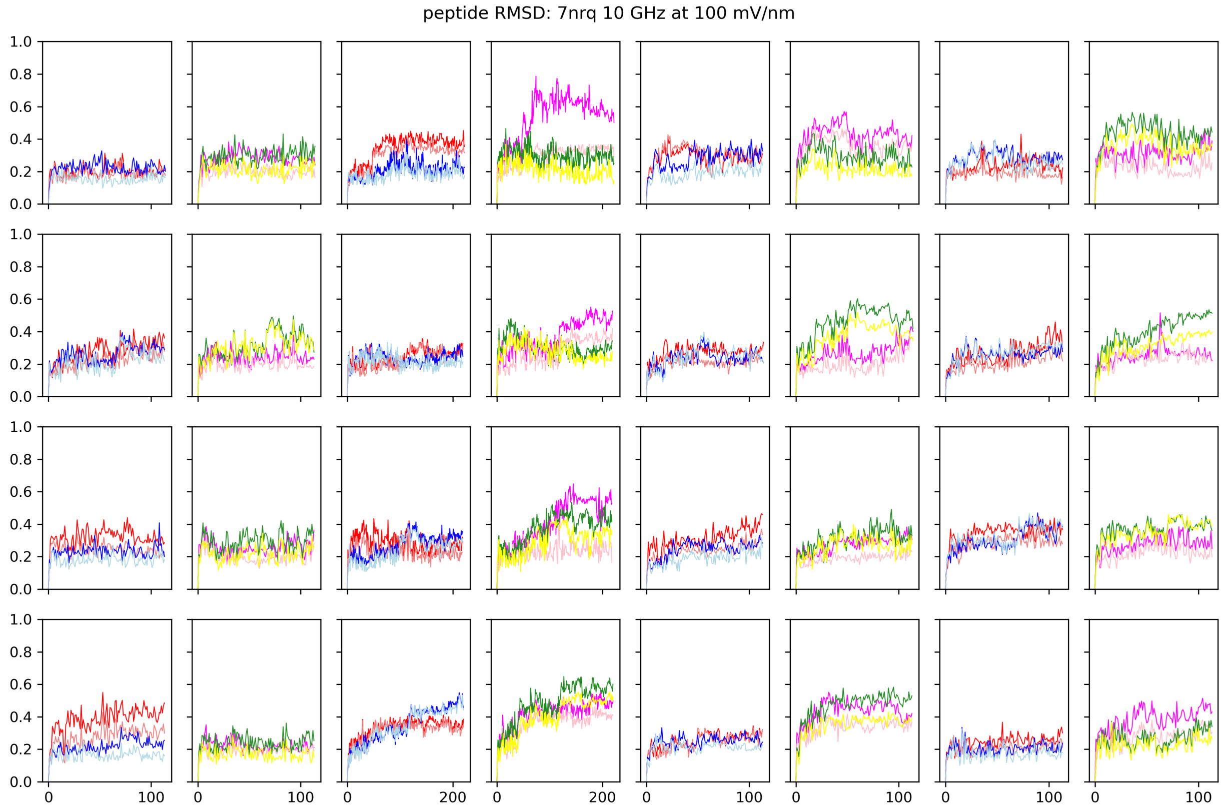

Figure S 33: Time-resolved root mean square deviation of tip peptides of PHF with a 100 mV/nm oeEF oscillating at 10 GHz. Same visualization as Figure S30

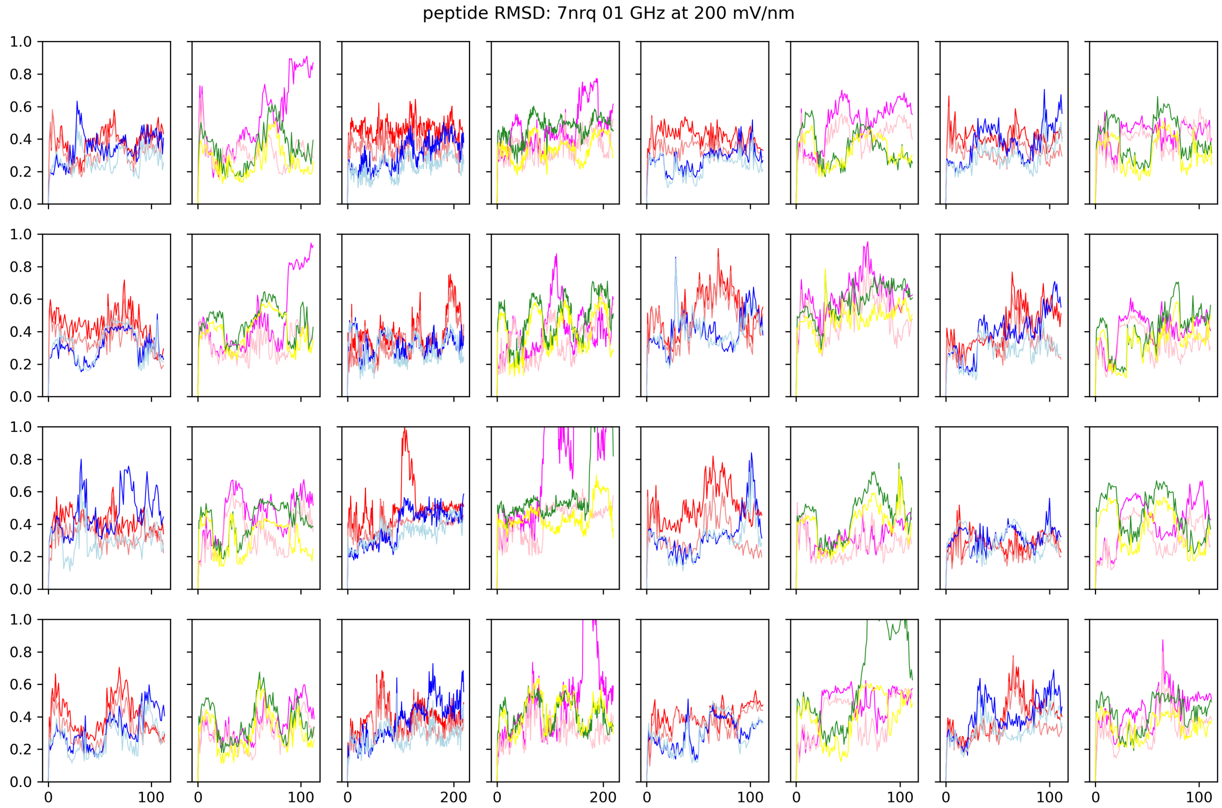

Figure S 34: Time-resolved root mean square deviation of tip peptides of PHF with a 200 mV/nm oeEF oscillating at 0.1 GHz. Same visualization as Figure S30

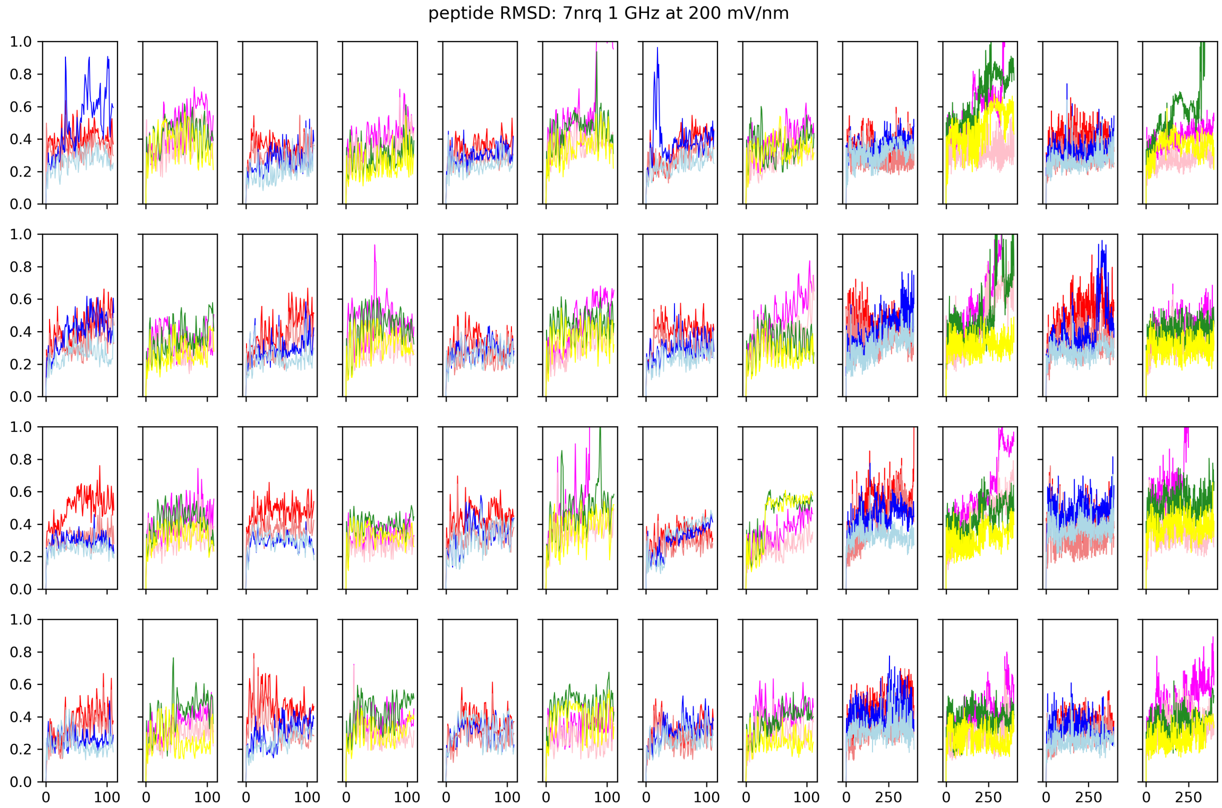

Figure S 35: Time-resolved root mean square deviation of tip peptides of PHF with a 200 mV/nm oeEF oscillating at 1 GHz. Same visualization as Figure S30

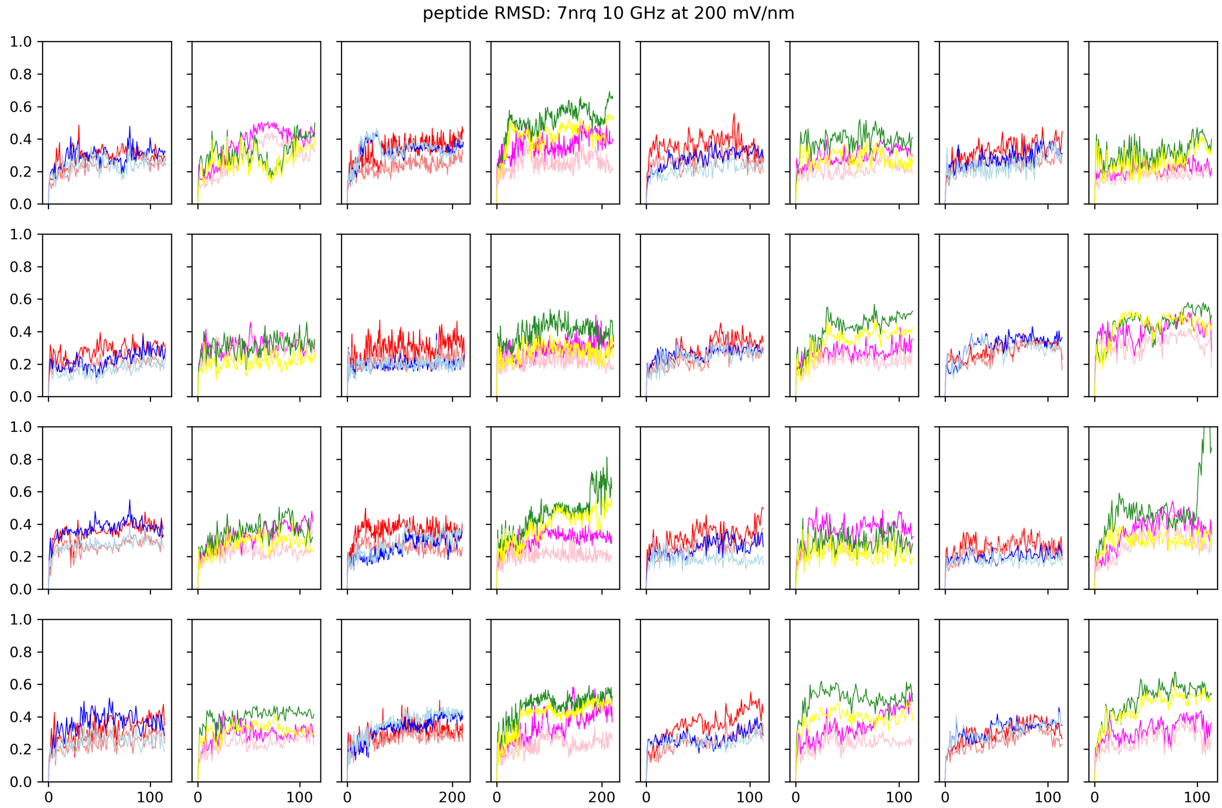

Figure S 36: Time-resolved root mean square deviation of tip peptides of PHF with a 200 mV/nm oeEF oscillating at 10 GHz. Same visualization as Figure S30

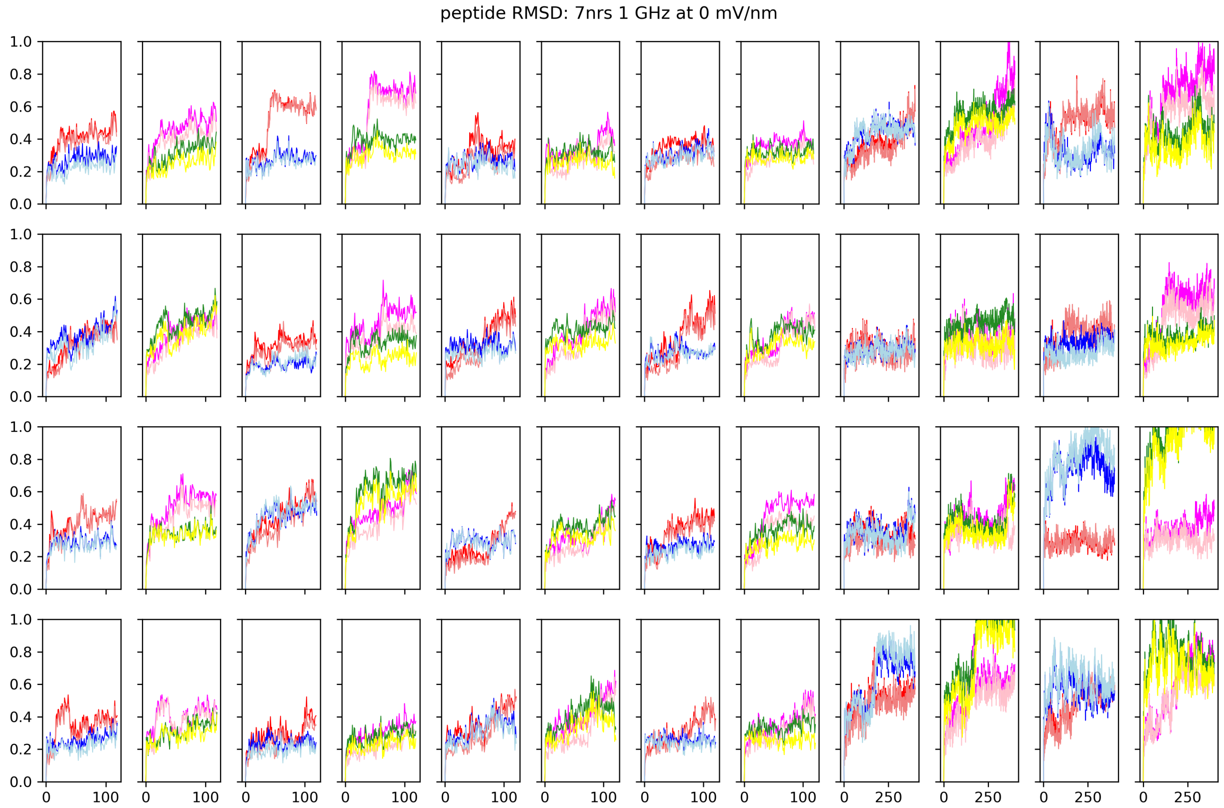

Figure S 37: Time-resolved root mean square deviation of tip peptides of SF without an oeEF. Same visualization as Figure S30

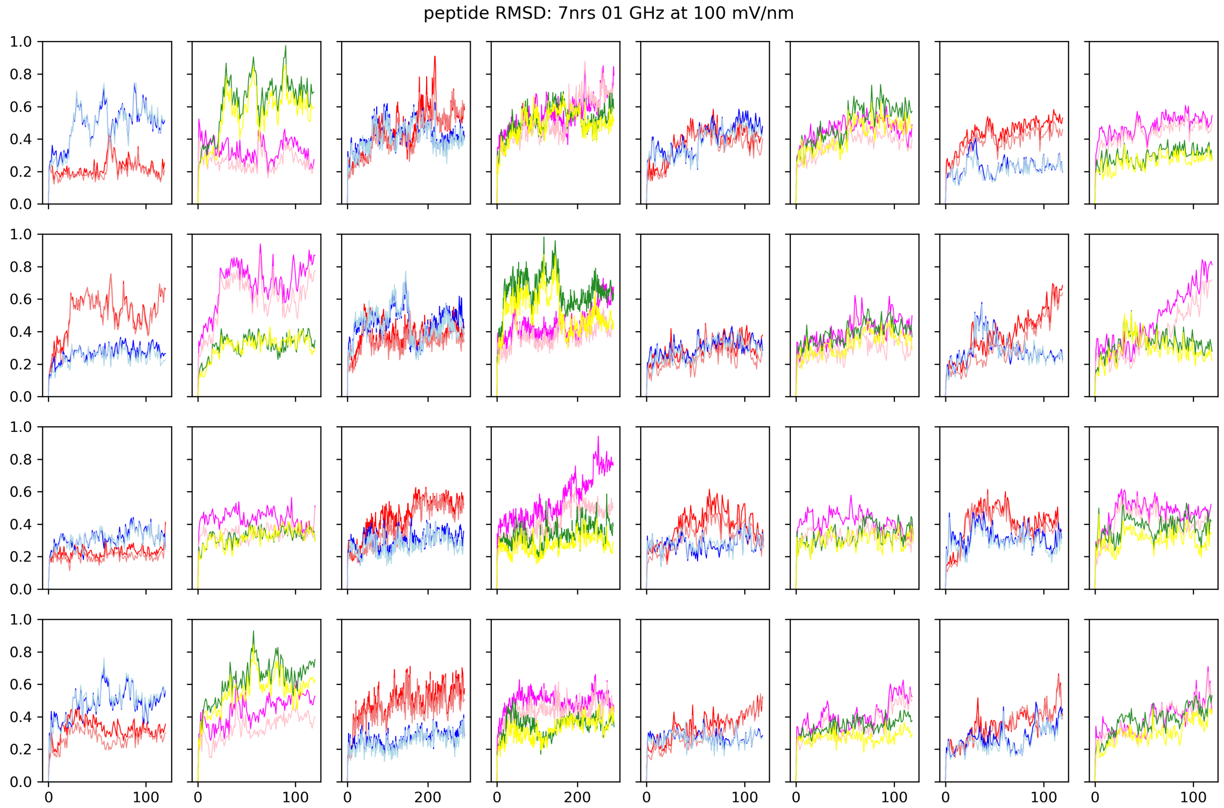

Figure S 38: Time-resolved root mean square deviation of tip peptides of SF with a 100 mV/nm oeEF oscillating at 0.1 GHz. Same visualization as Figure S30

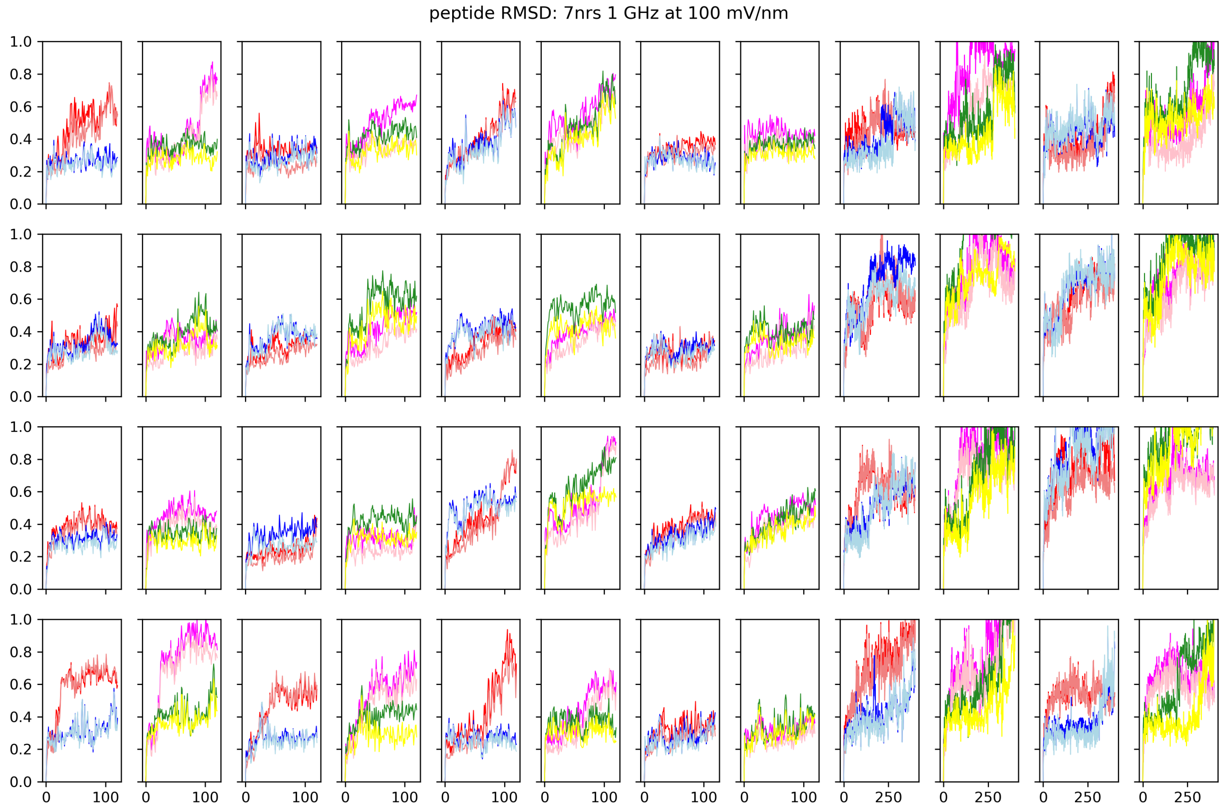

Figure S 39: Time-resolved root mean square deviation of tip peptides of SF with a 100 mV/nm oeEF oscillating at 1 GHz. Same visualization as Figure S30

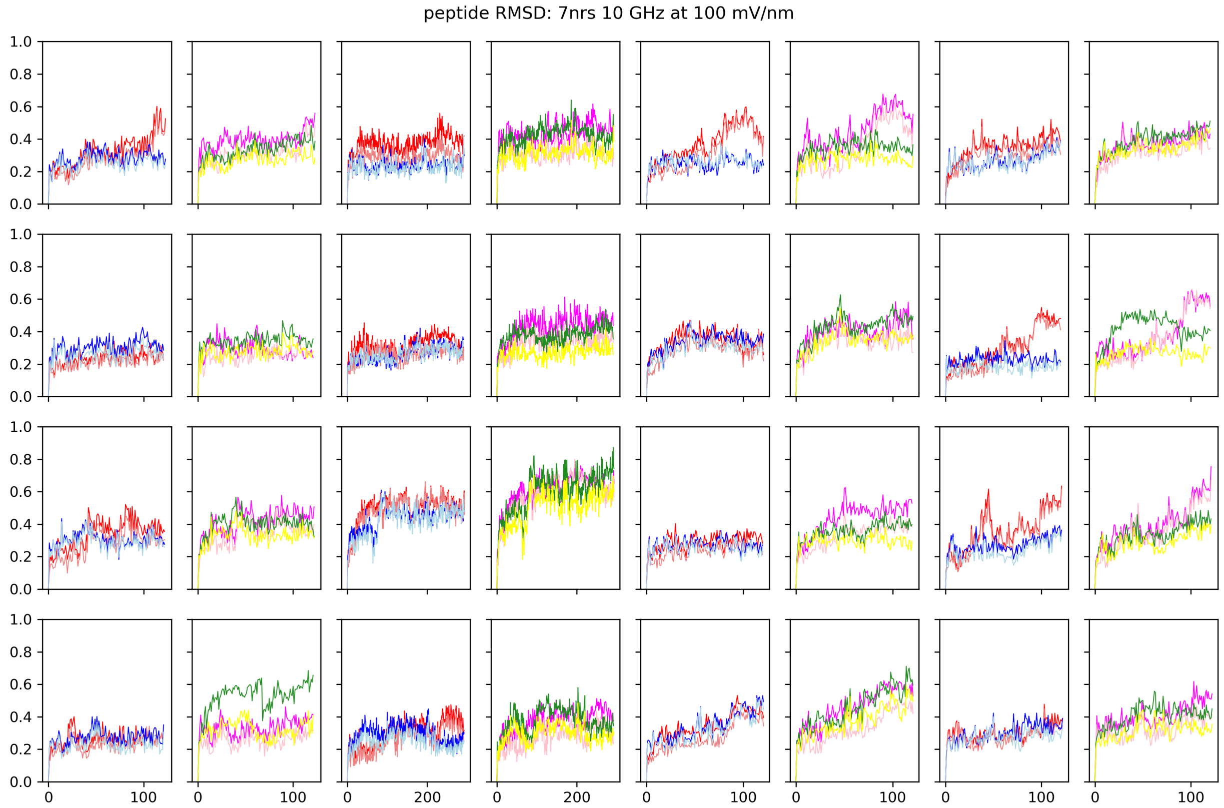

Figure S 40: Time-resolved root mean square deviation of tip peptides of SF with a 100 mV/nm oeEF oscillating at 10 GHz. Same visualization as Figure S30

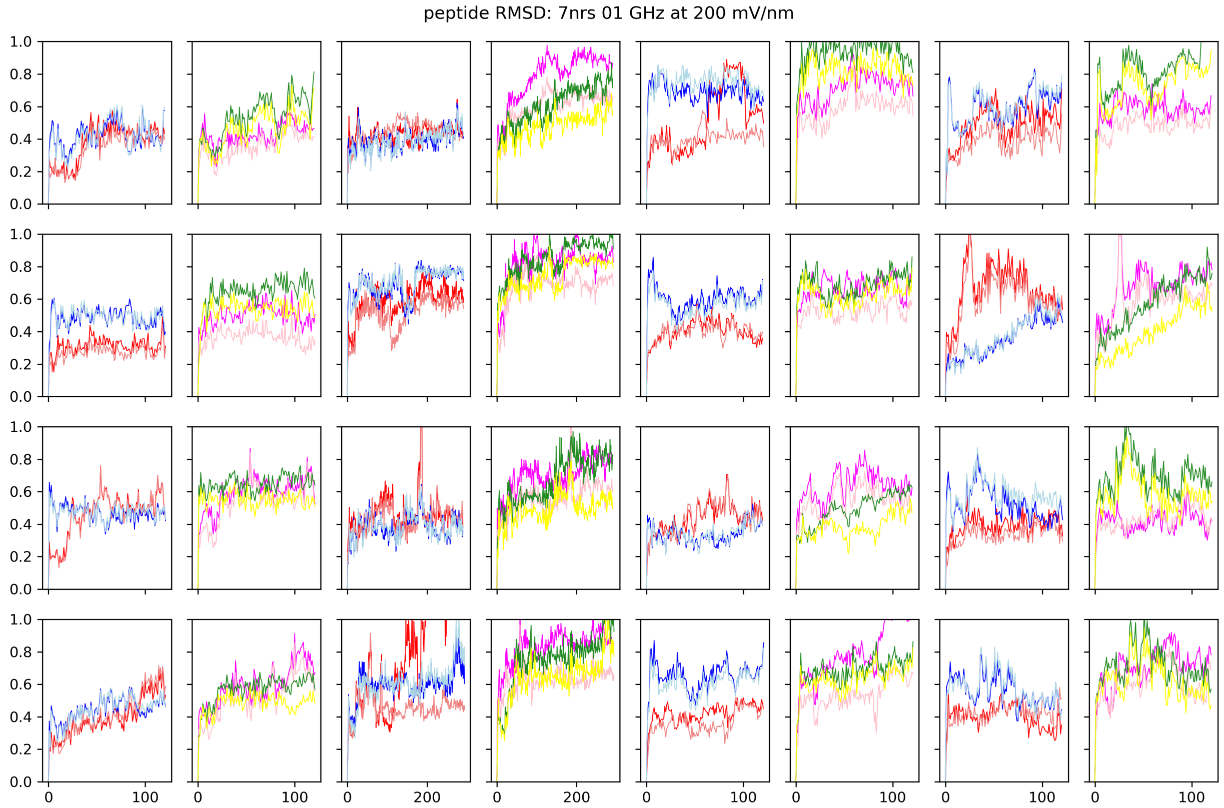

Figure S 41: Time-resolved root mean square deviation of tip peptides of SF with a 200 mV/nm oeEF oscillating at 0.1 GHz. Same visualization as Figure S30

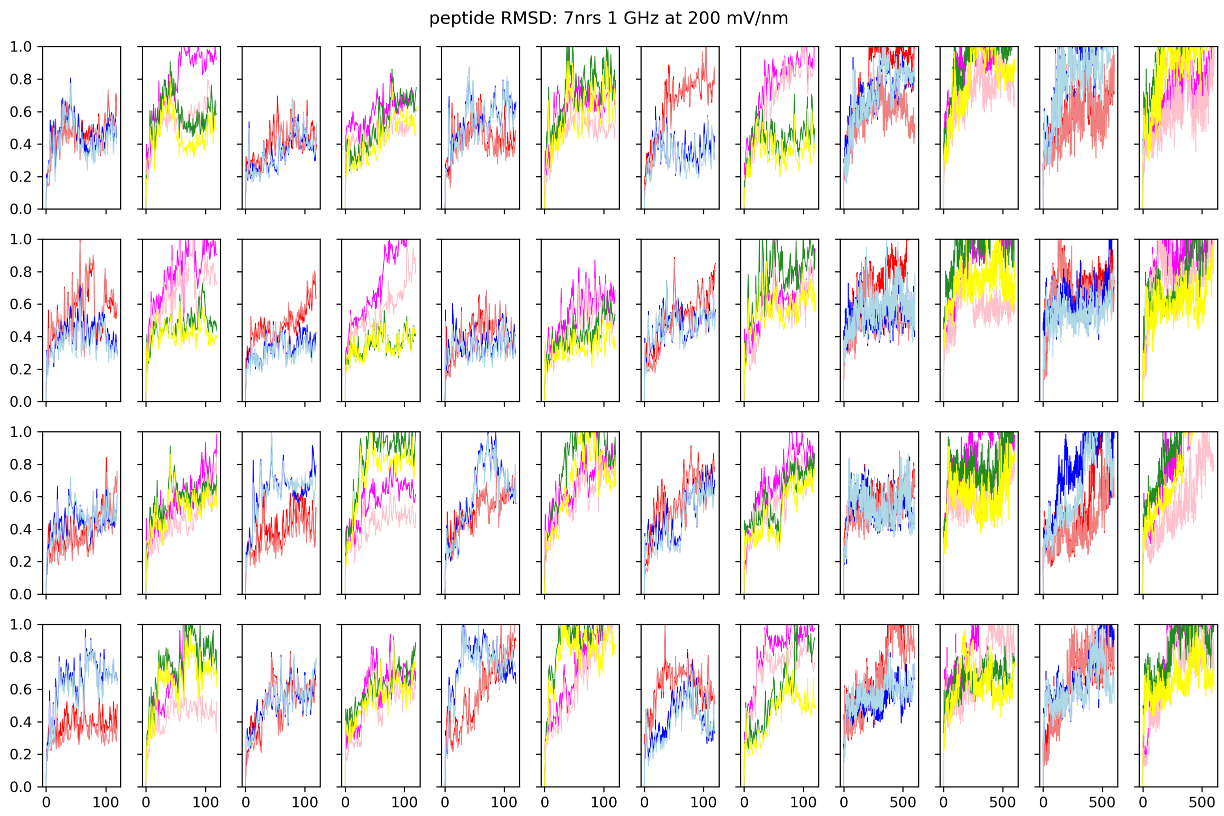

Figure S 42: Time-resolved root mean square deviation of tip peptides of SF with a 200 mV/nm oeEF oscillating at 1 GHz. Same visualization as Figure S30

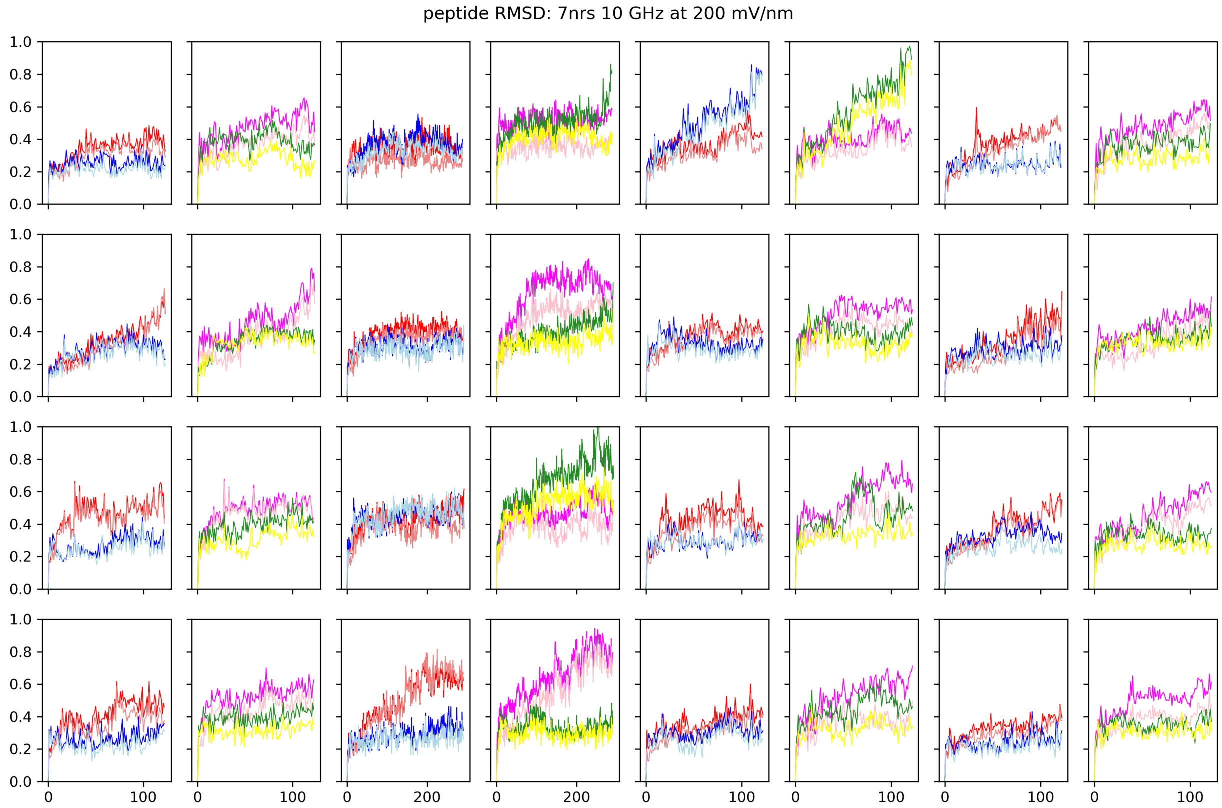

Figure S 43: Time-resolved root mean square deviation of tip peptides of SF with a 200 mV/nm oeEF oscillating at 10 GHz. Same visualization as Figure S30

Implicit solvent simulations

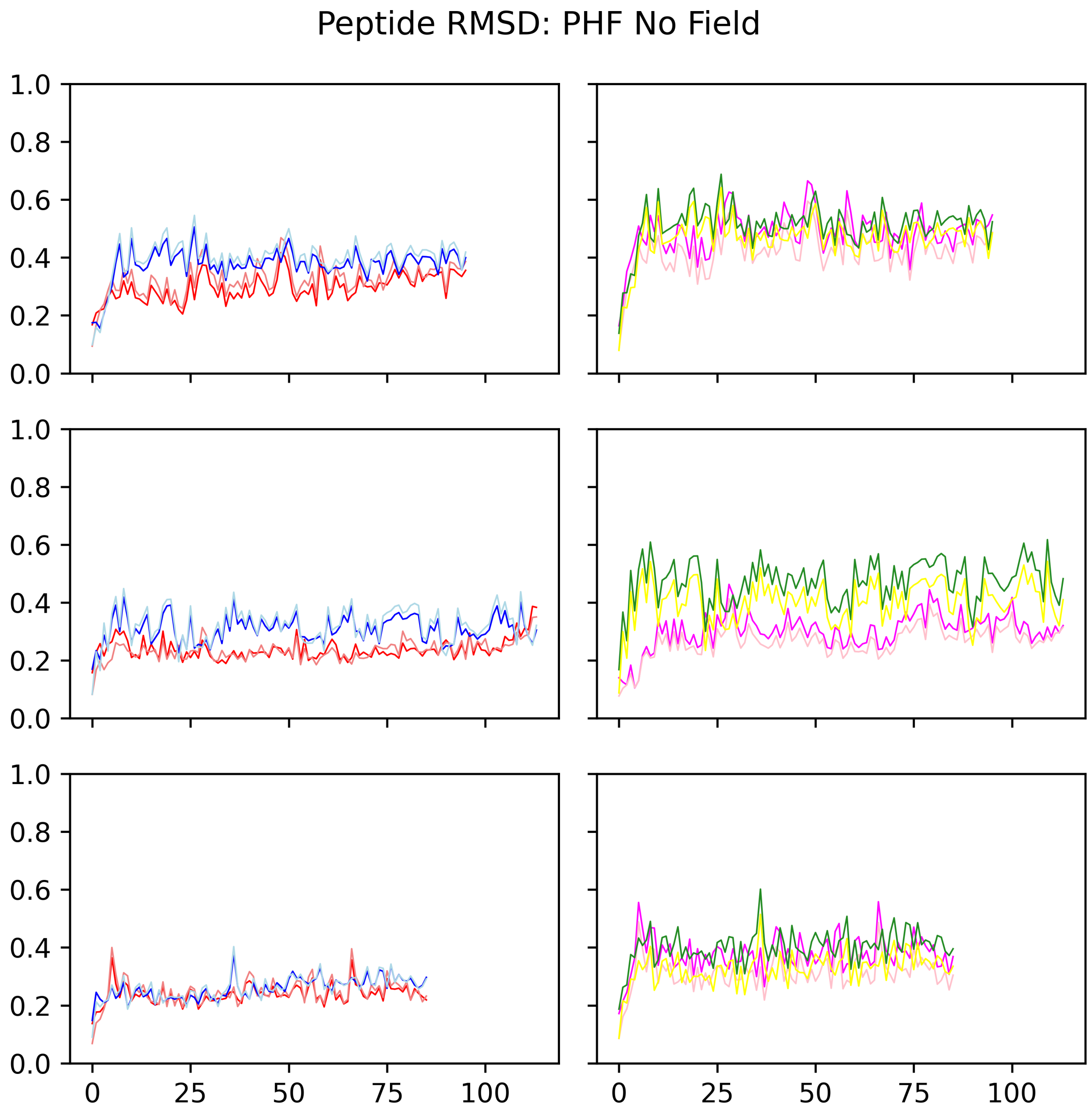

Figure S 44: Time-resolved root mean square deviation of tip peptides of PHF without oeEF in implicit solvent. Same visualization as Figure S30

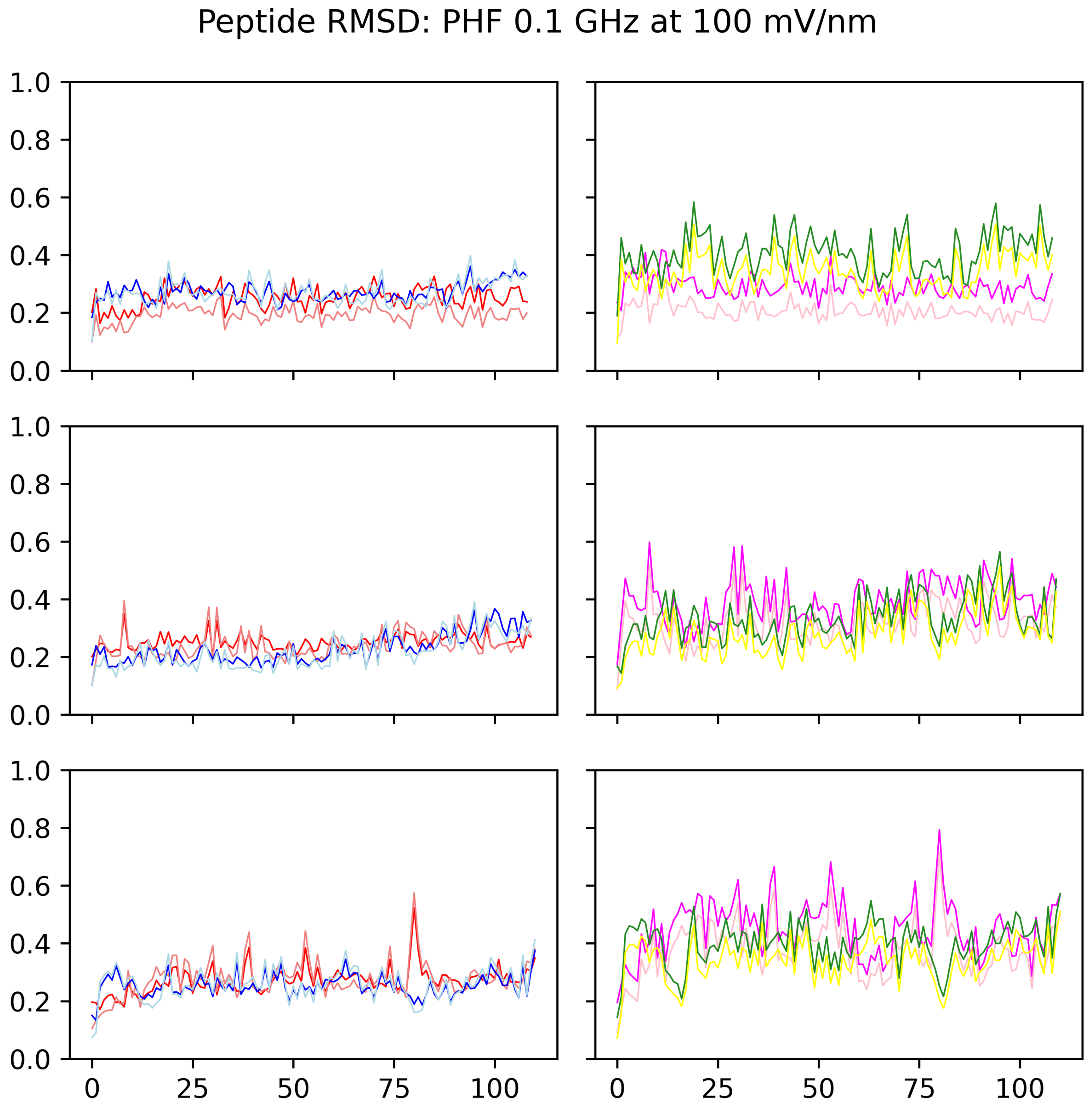

Figure S 45: Time-resolved root mean square deviation of tip peptides of PHF with a 100 mV/nm oeEF oscillating at 0.1 GHz in implicit solvent. Same visualization as Figure S30

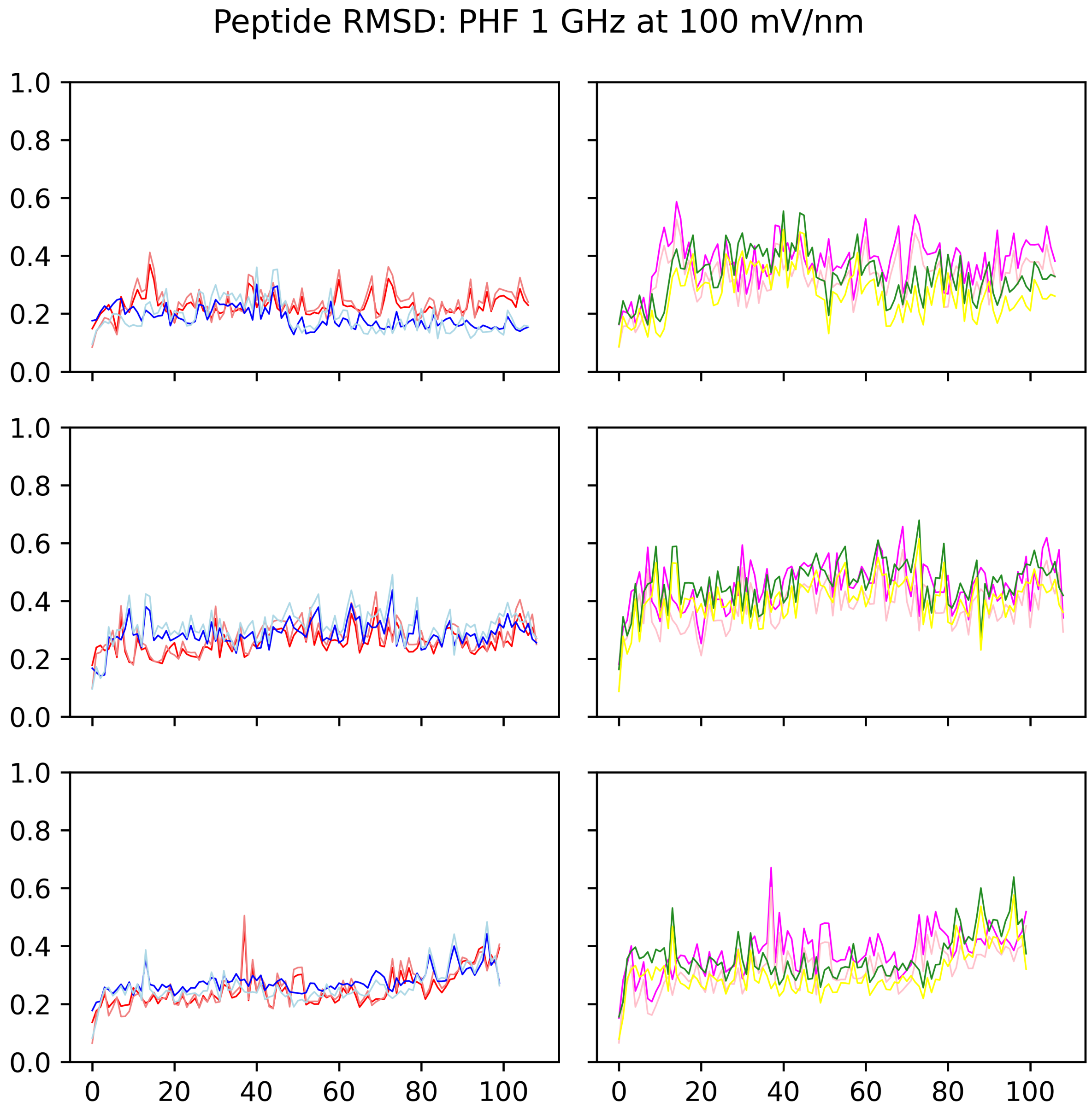

Figure S 46: Time-resolved root mean square deviation of tip peptides of PHF with a 100 mV/nm oeEF oscillating at 1 GHz in implicit solvent. Same visualization as Figure S30

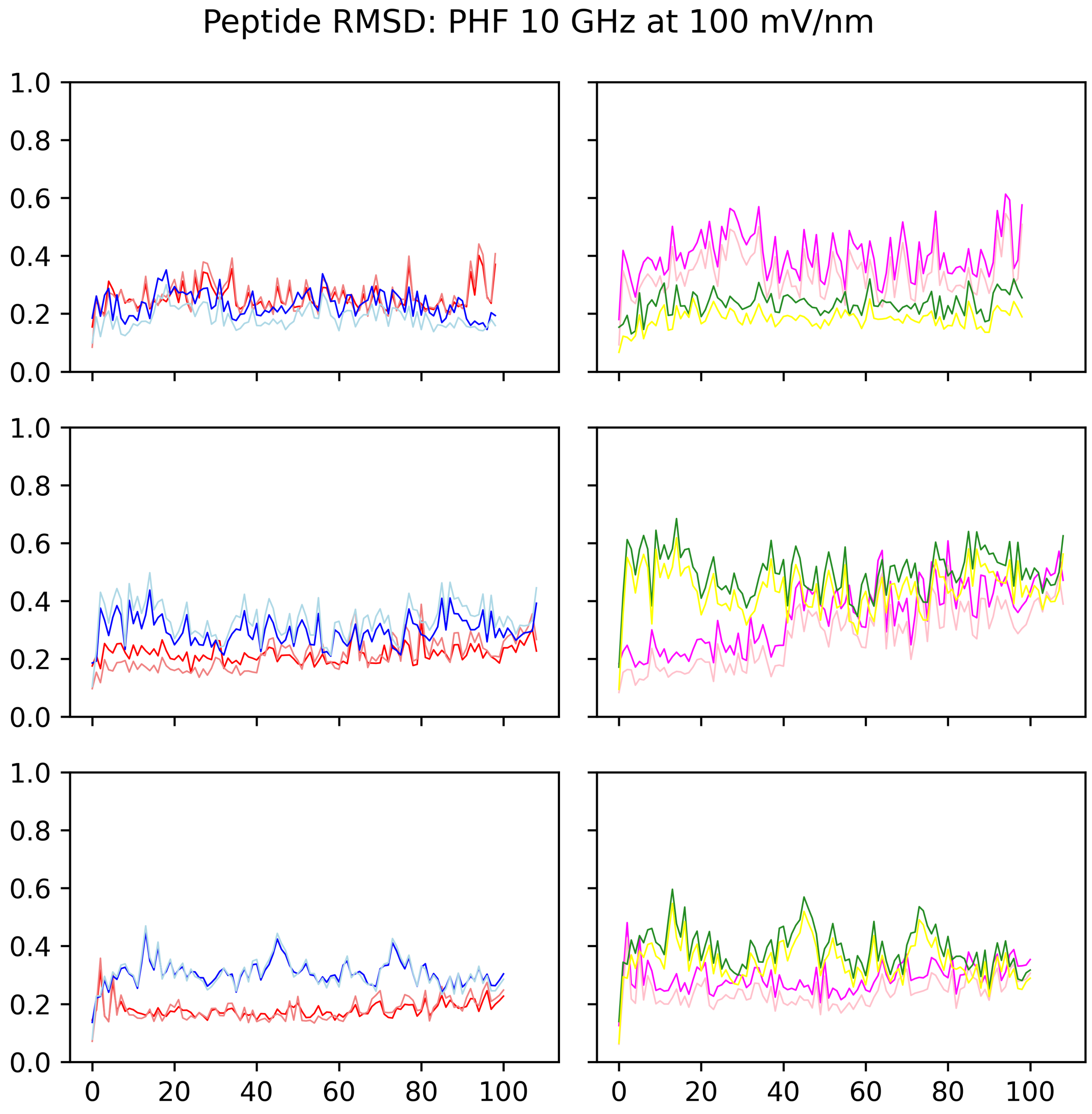

Figure S 47: Time-resolved root mean square deviation of tip peptides of PHF with a 100 mV/nm oeEF oscillating at 10 GHz in implicit solvent. Same visualization as Figure S30

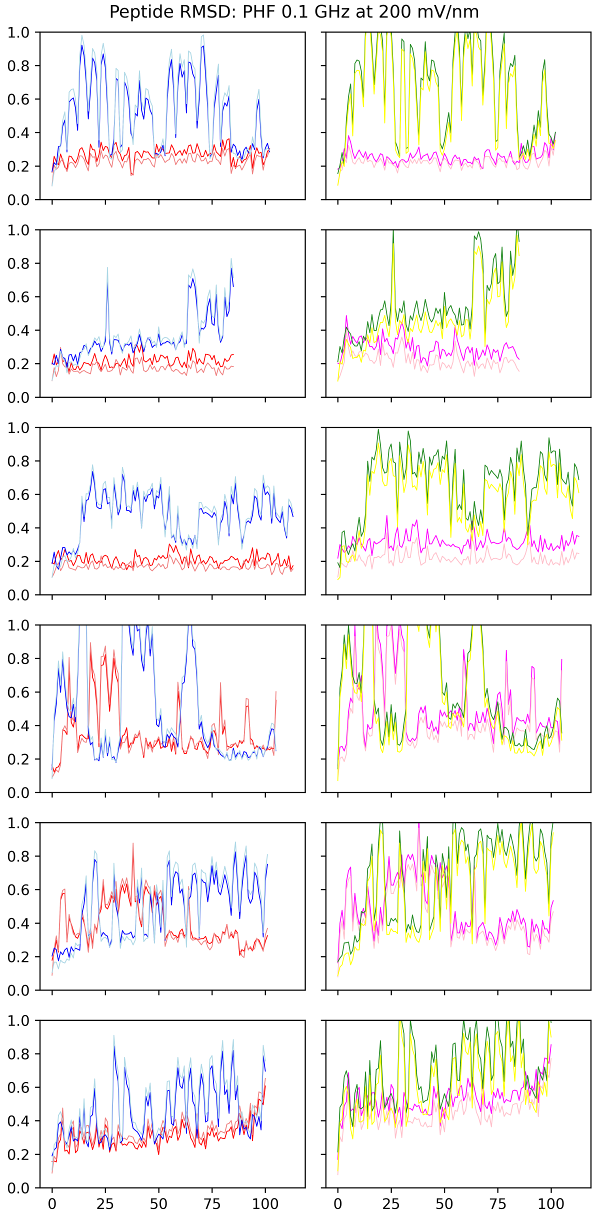

Figure S 48: Time-resolved root mean square deviation of tip peptides of PHF with a 200 mV/nm oeEF oscillating at 0.1 GHz in implicit solvent. Same visualization as Figure S30

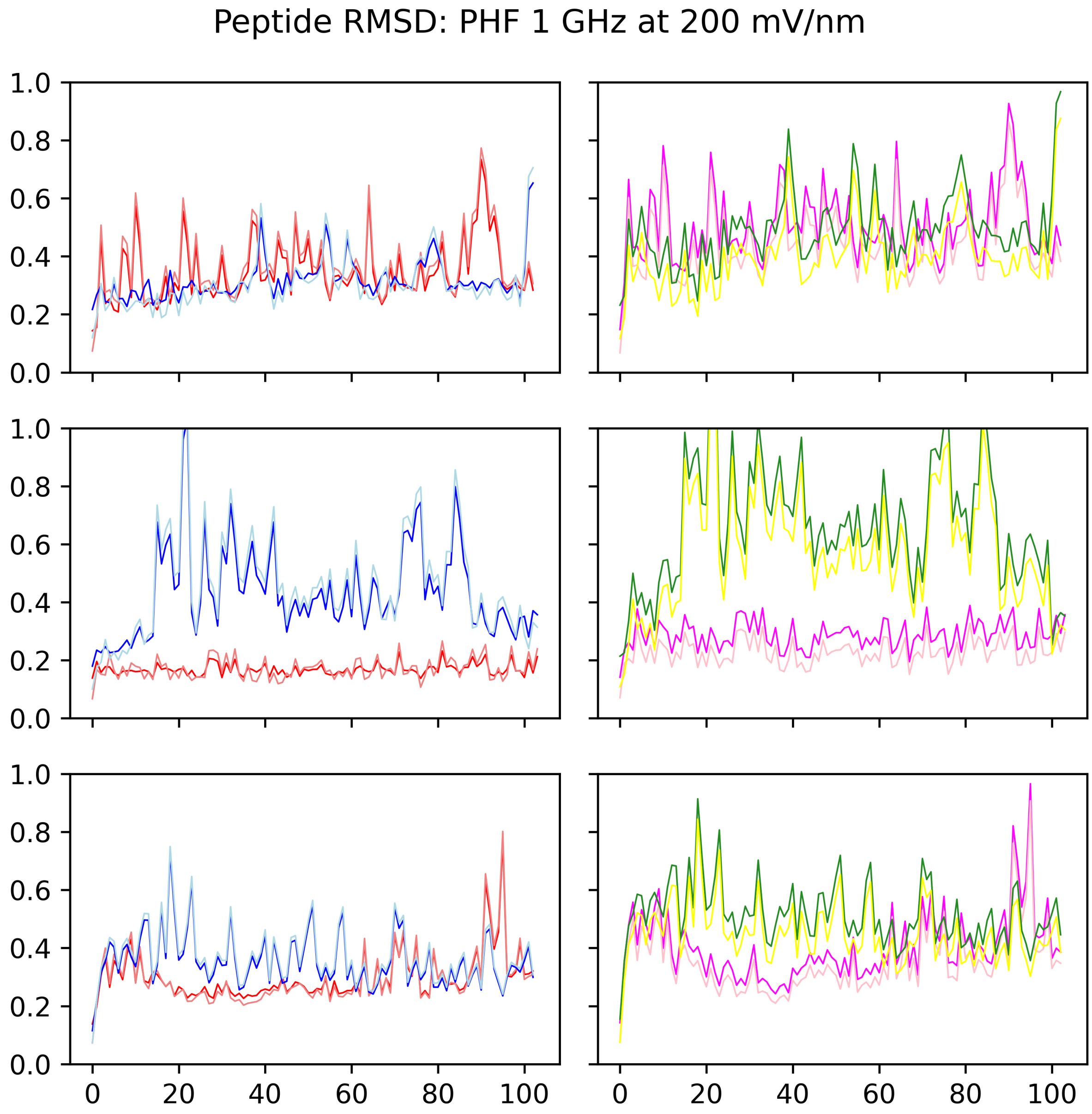

Figure S 49: Time-resolved root mean square deviation of tip peptides of PHF with a 200 mV/nm oeEF oscillating at 1 GHz in implicit solvent. Same visualization as Figure S30

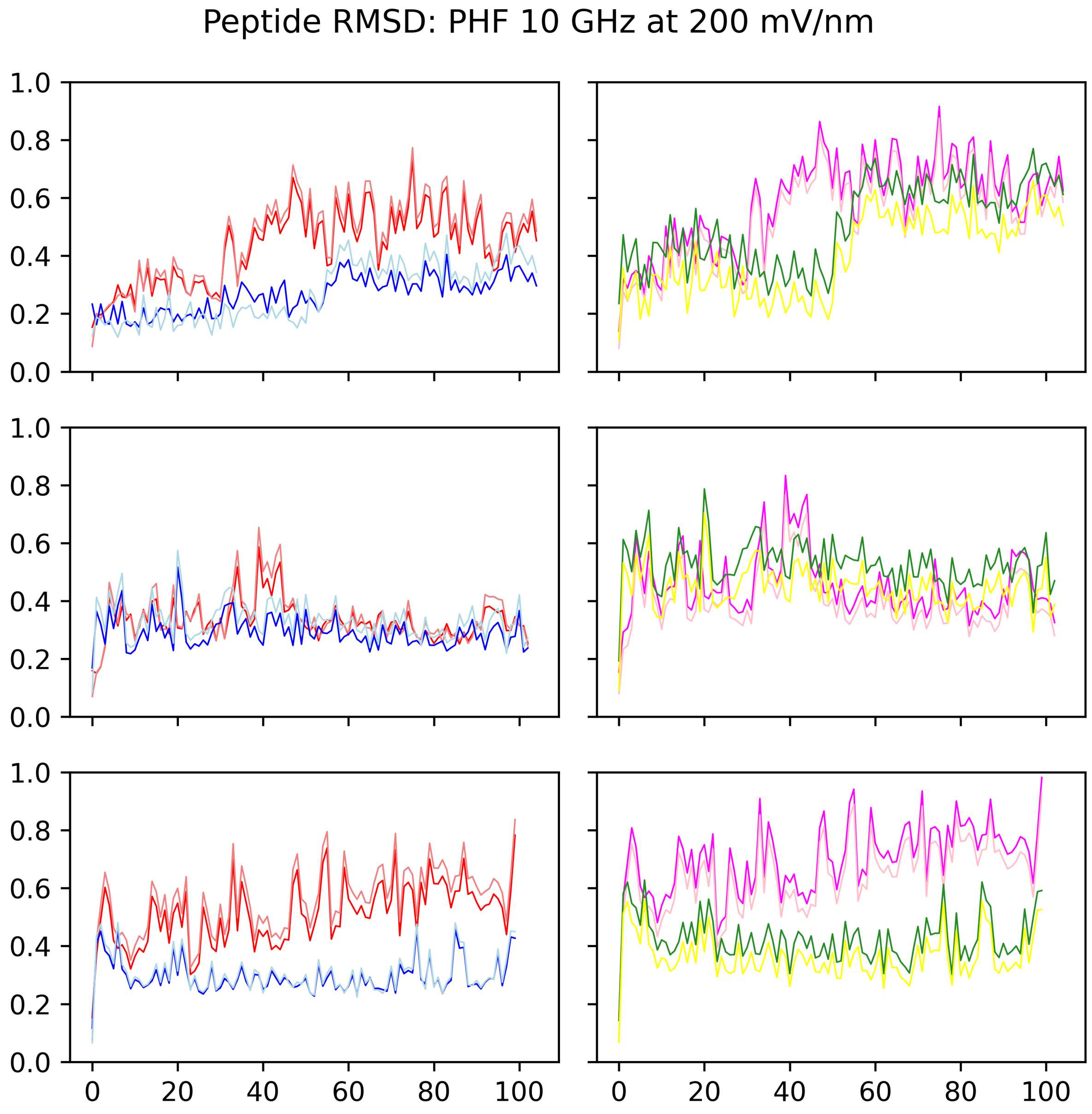

Figure S 50: Time-resolved root mean square deviation of tip peptides of PHF with a 200 mV/nm oeEF oscillating at 10 GHz in implicit solvent. Same visualization as Figure S30

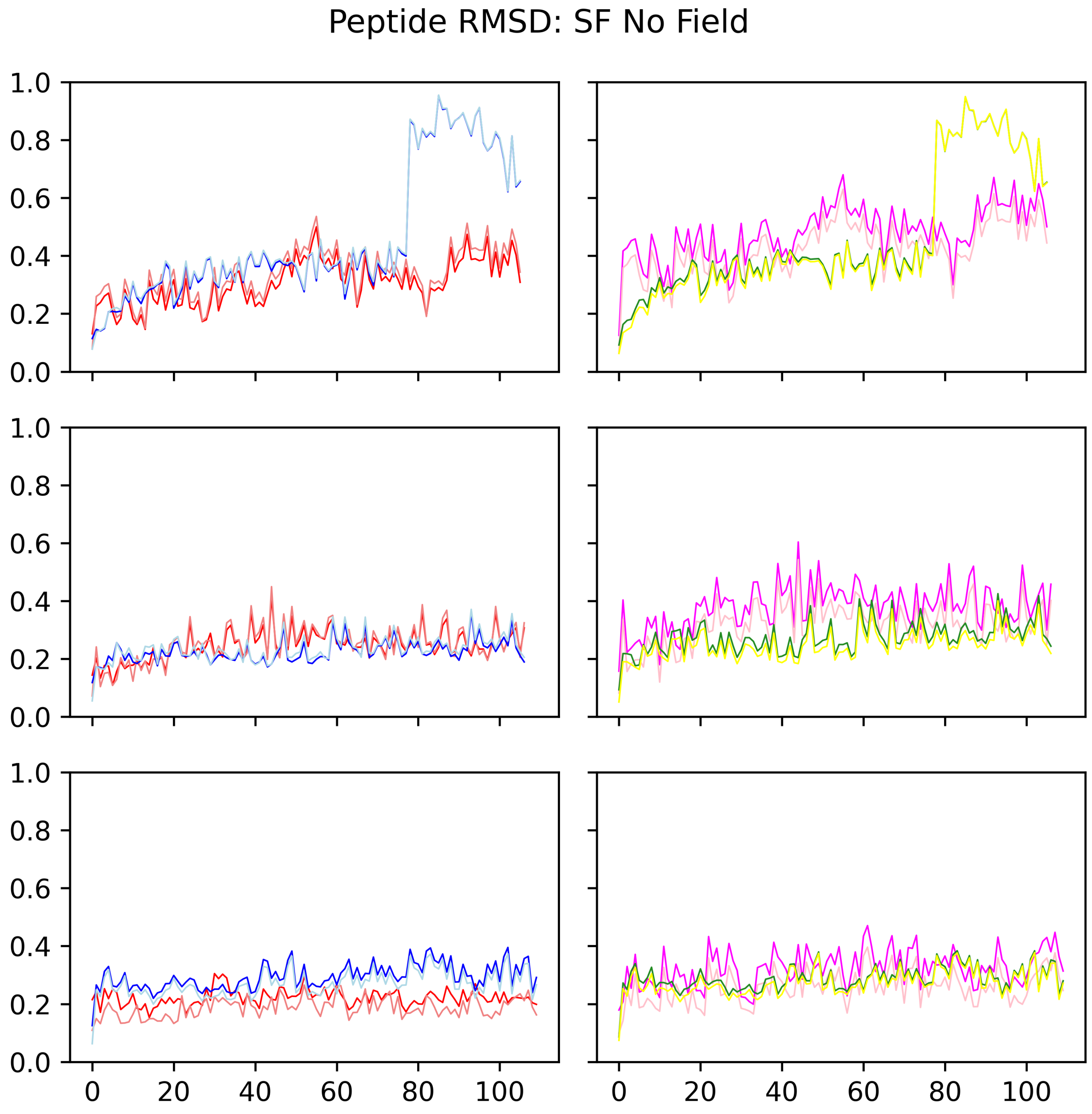

Figure S 51: Time-resolved root mean square deviation of tip peptides of SF without an oeEF in implicit solvent. Same visualization as Figure S30

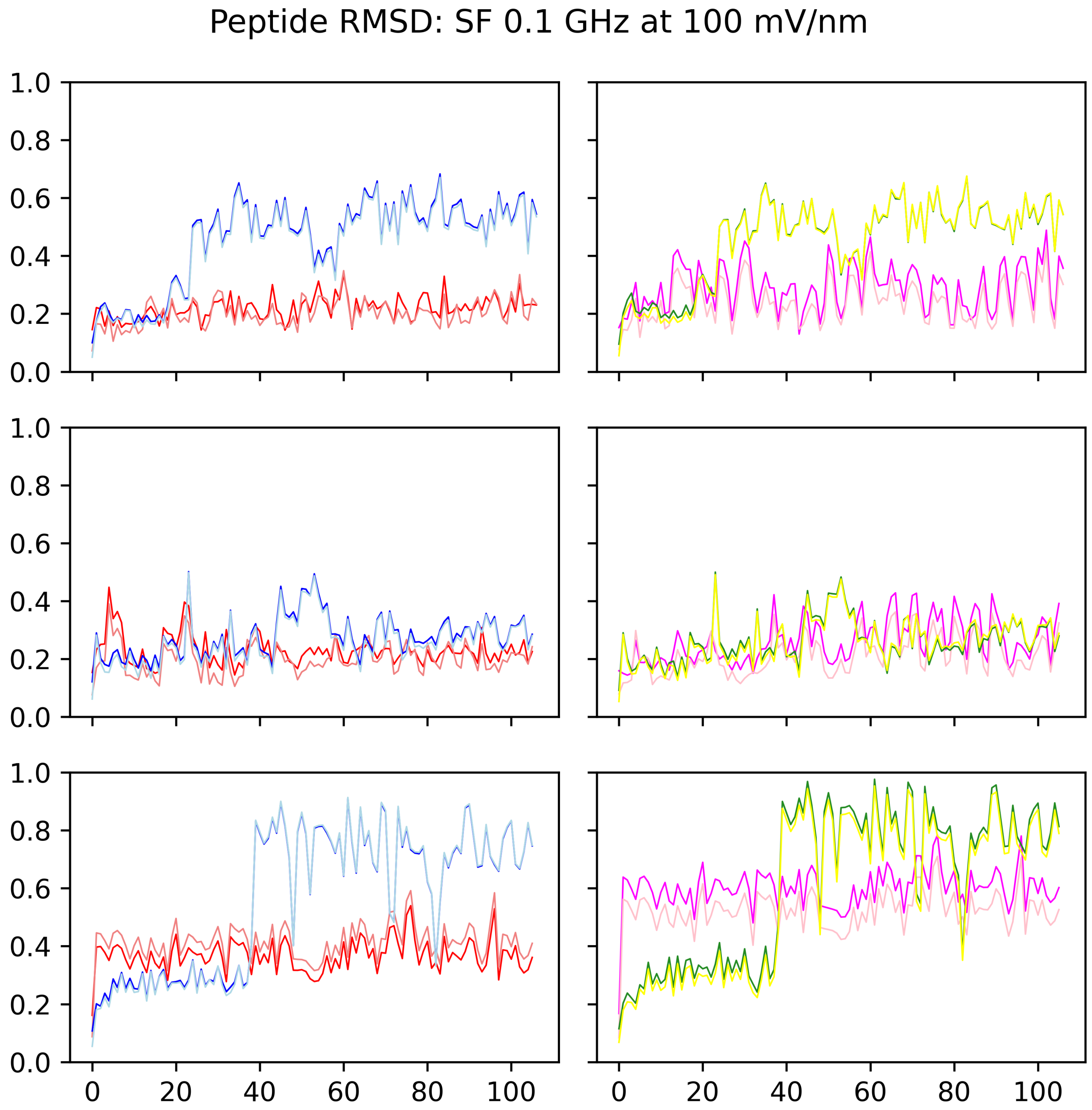

Figure S 52: Time-resolved root mean square deviation of tip peptides of SF with a 100 mV/nm oeEF oscillating at 0.1 GHz in implicit solvent. Same visualization as Figure S30

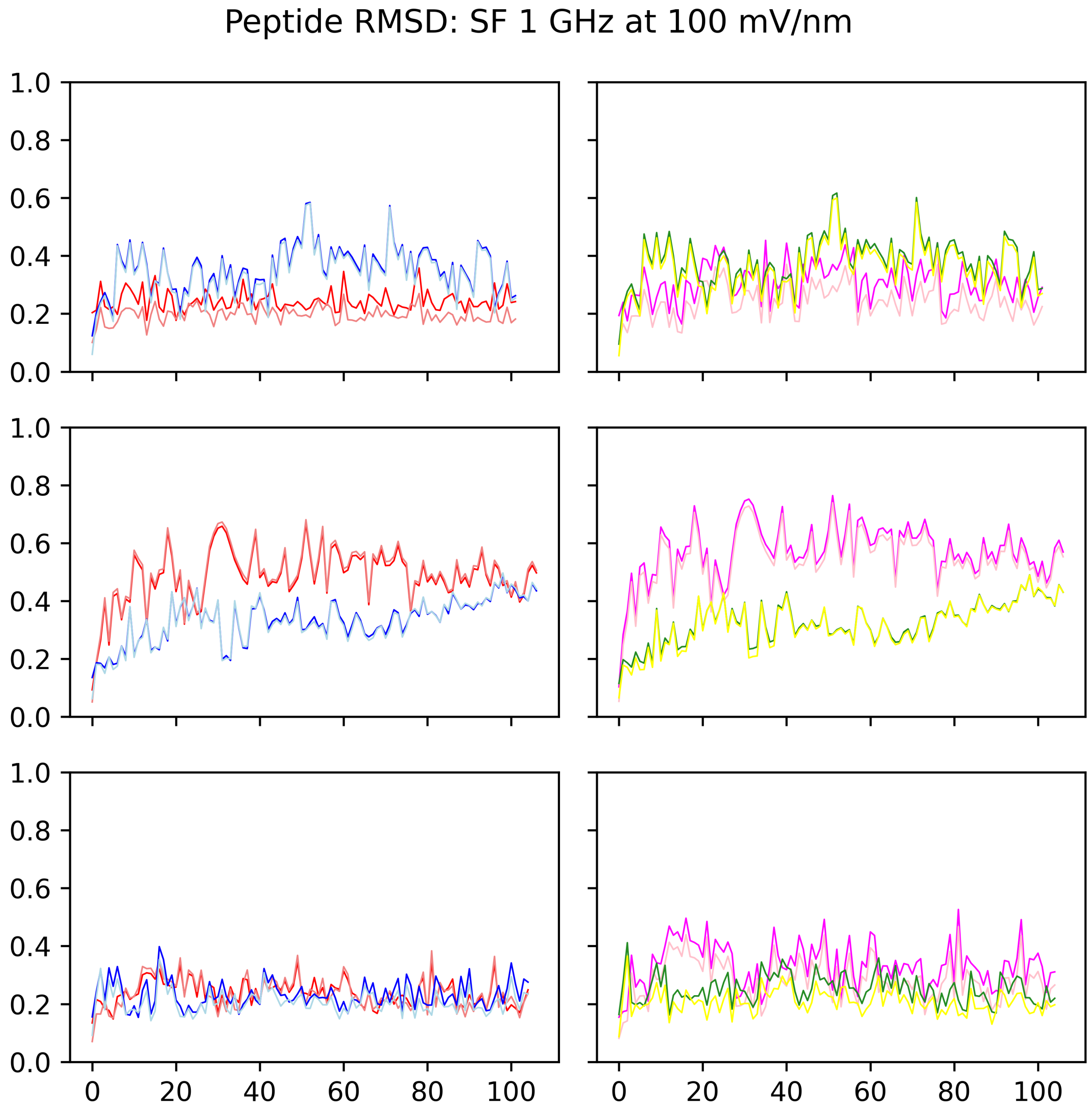

Figure S 53: Time-resolved root mean square deviation of tip peptides of SF with a 100 mV/nm oeEF oscillating at 1 GHz in implicit solvent. Same visualization as Figure S30

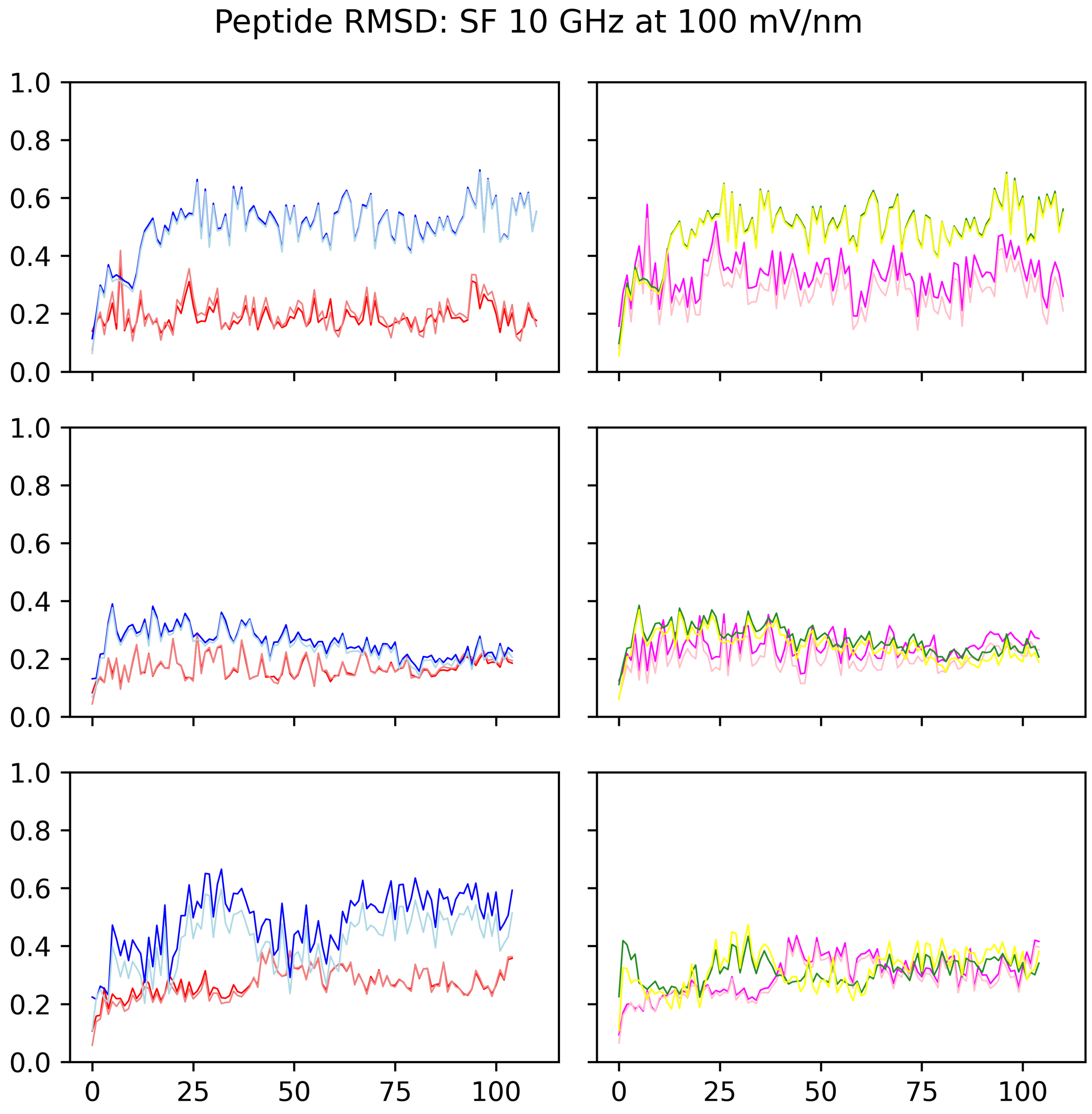

Figure S 54: Time-resolved root mean square deviation of tip peptides of SF with a 100 mV/nm oeEF oscillating at 10 GHz in implicit solvent. Same visualization as Figure S30

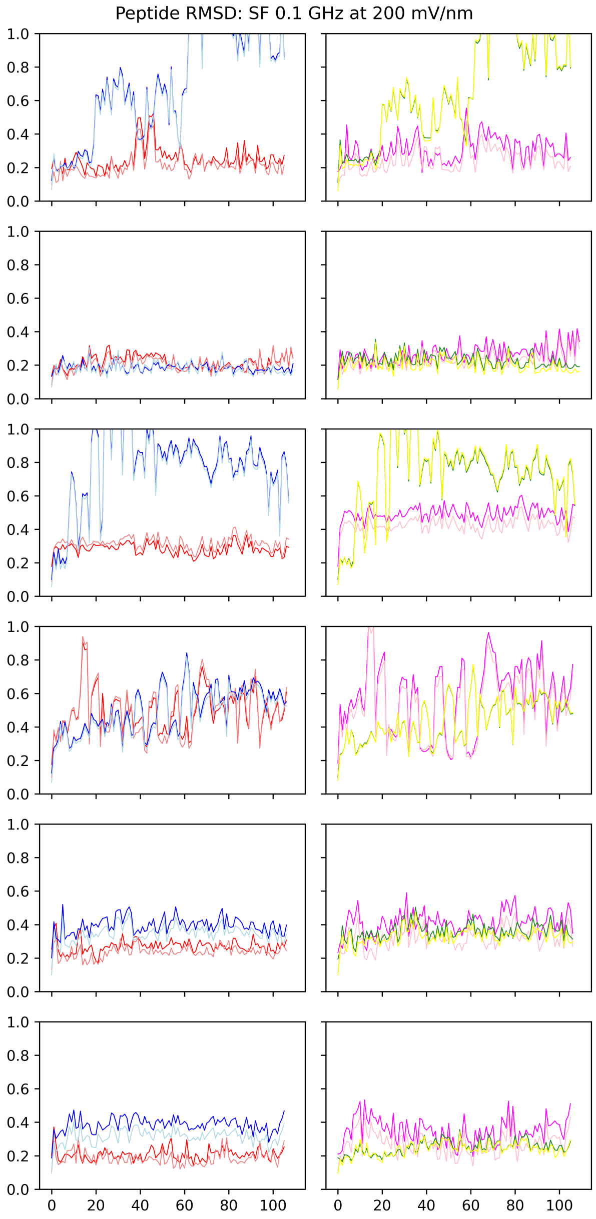

Figure S 55: Time-resolved root mean square deviation of tip peptides of SF with a 200 mV/nm oeEF oscillating at 0.1 GHz in implicit solvent. Same visualization as Figure S30

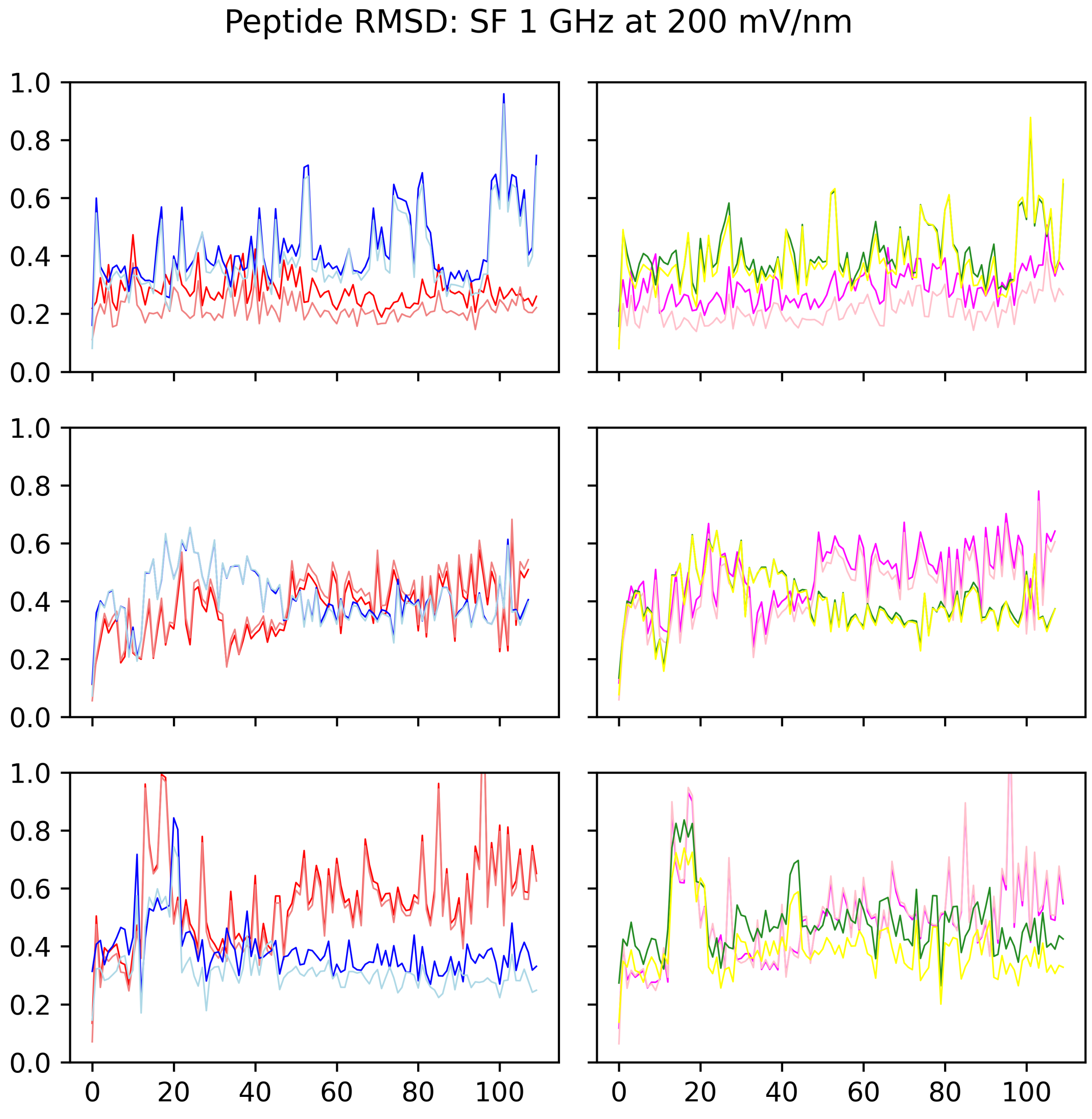

Figure S 56: Time-resolved root mean square deviation of tip peptides of SF with a 200 mV/nm oeEF oscillating at 1 GHz in implicit solvent. Same visualization as Figure S30

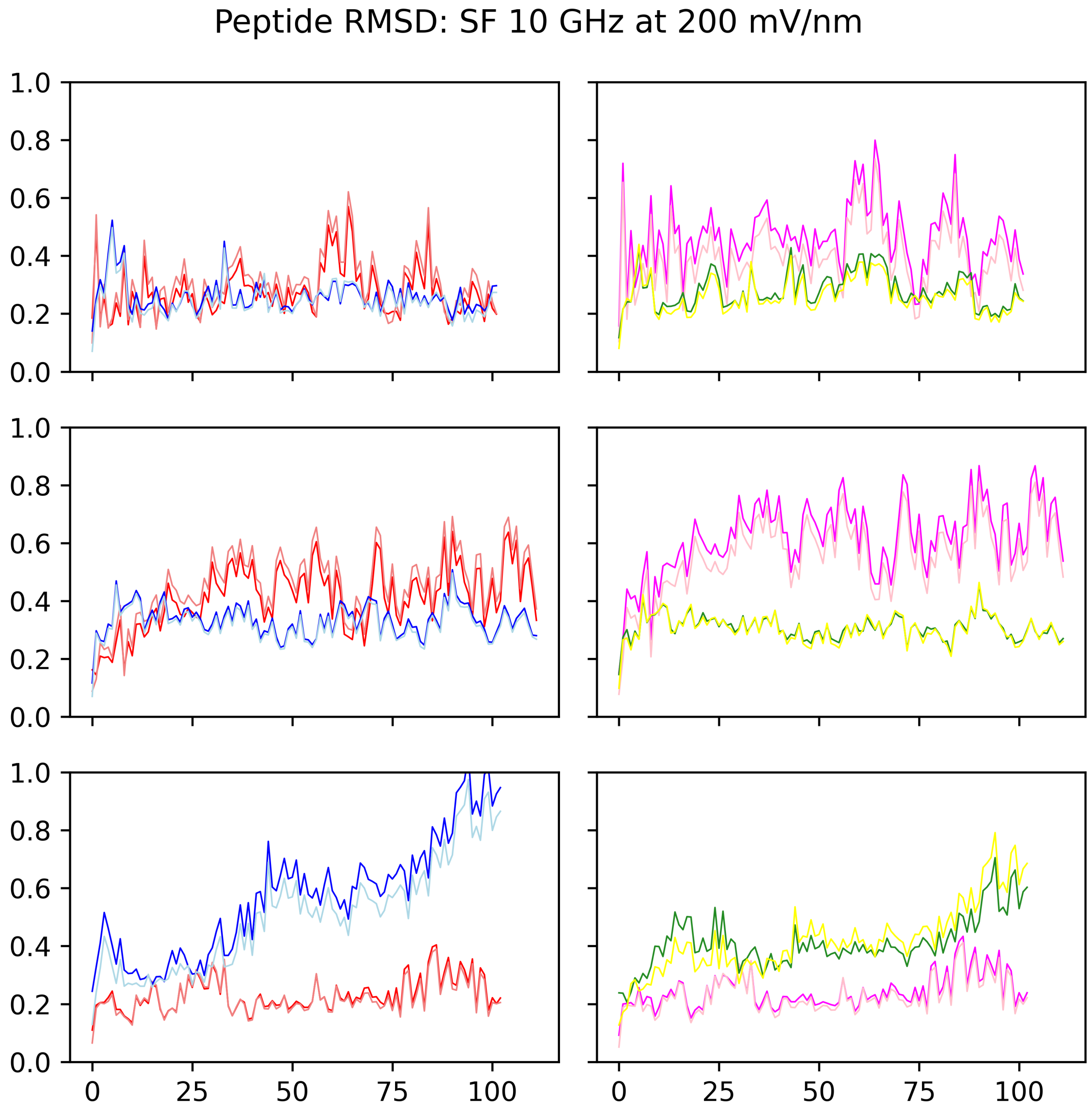

Figure S 57: Time-resolved root mean square deviation of tip peptides of SF with a 200 mV/nm oeEF oscillating at 10 GHz in implicit solvent. Same visualization as Figure S30

Exponential decay of the protofibril β-strand content

We show here the exponential fit of the β-strand content at each condition of oeEF. Four different models were tested: one parameter (Model 1, exponential decay: $e^{-x/B}$), two parameters (Model 2, $Ae^{-x/B}$), three parameters (Model 3, two complementary reaction channels, $Ae^{-x/B} + (1-A)e^{-x/D}$), and finally four parameters (Model 4, two independent reaction

channels, $Ae^{-x/B} + Ce^{-x/D}$).

Explicit solvent simulations

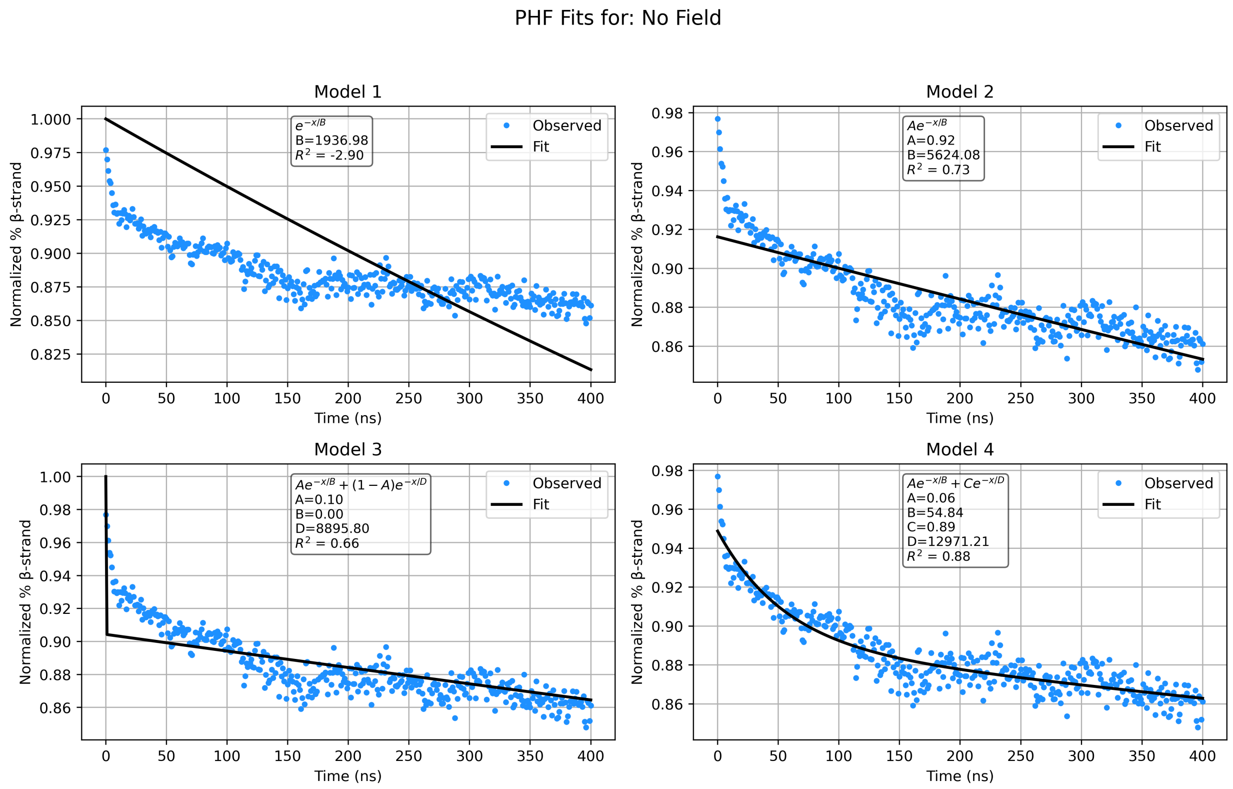

Figure S 58: Exponential fitting methods based on four different models of the β-strand content for the peptides of PHF without an oeEF. (Top Left) Model 1, one parameter ($e^{-x/B}$). (Top Right) Model 2, two parameters ($Ae^{-x/B}$). (Bottom Left) Model 3, three parameters ($Ae^{-x/B} + (1-A)e^{-x/D}$). (Bottom Right) Model 4, four parameters ($Ae^{-x/B} + Ce^{-x/D}$).

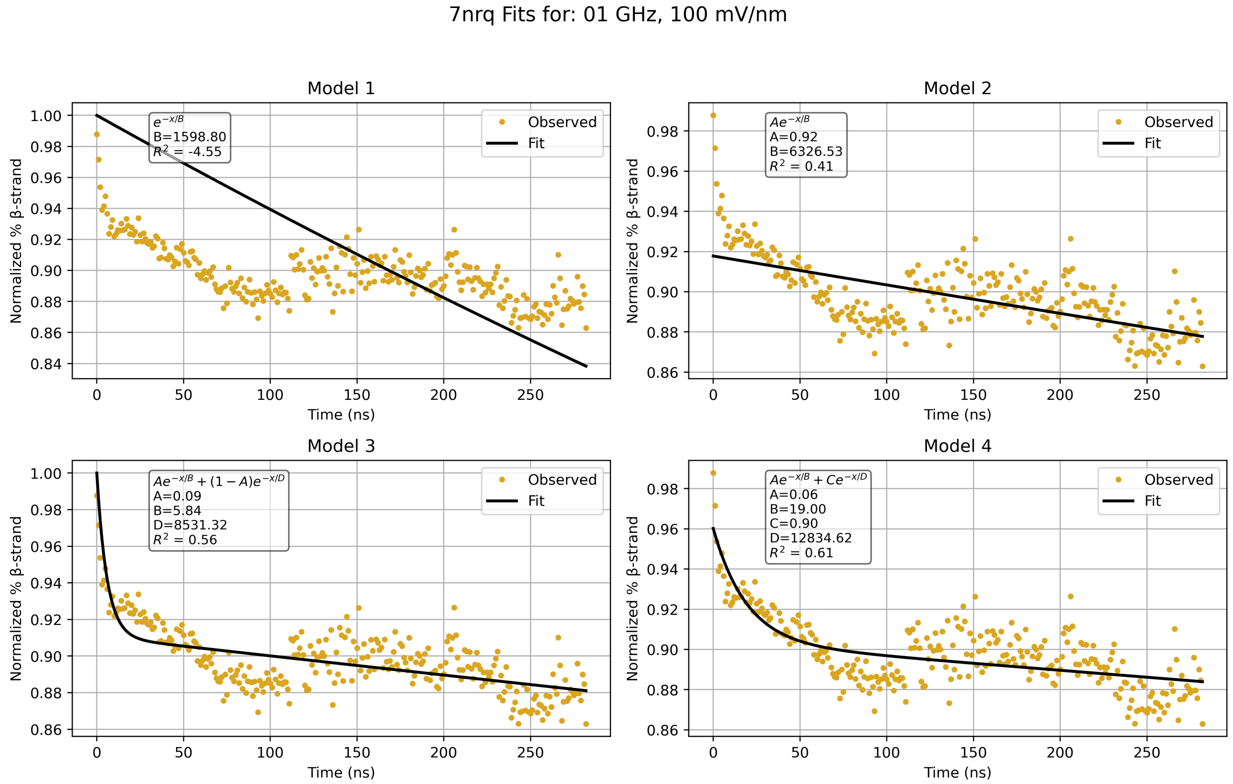

Figure S 59: Exponential fitting methods based on four different models of the β-strand content for the peptides of PHF with a 100 mV/nm oeEF oscillating at 0.1 GHz. Same visualization as Figure S58

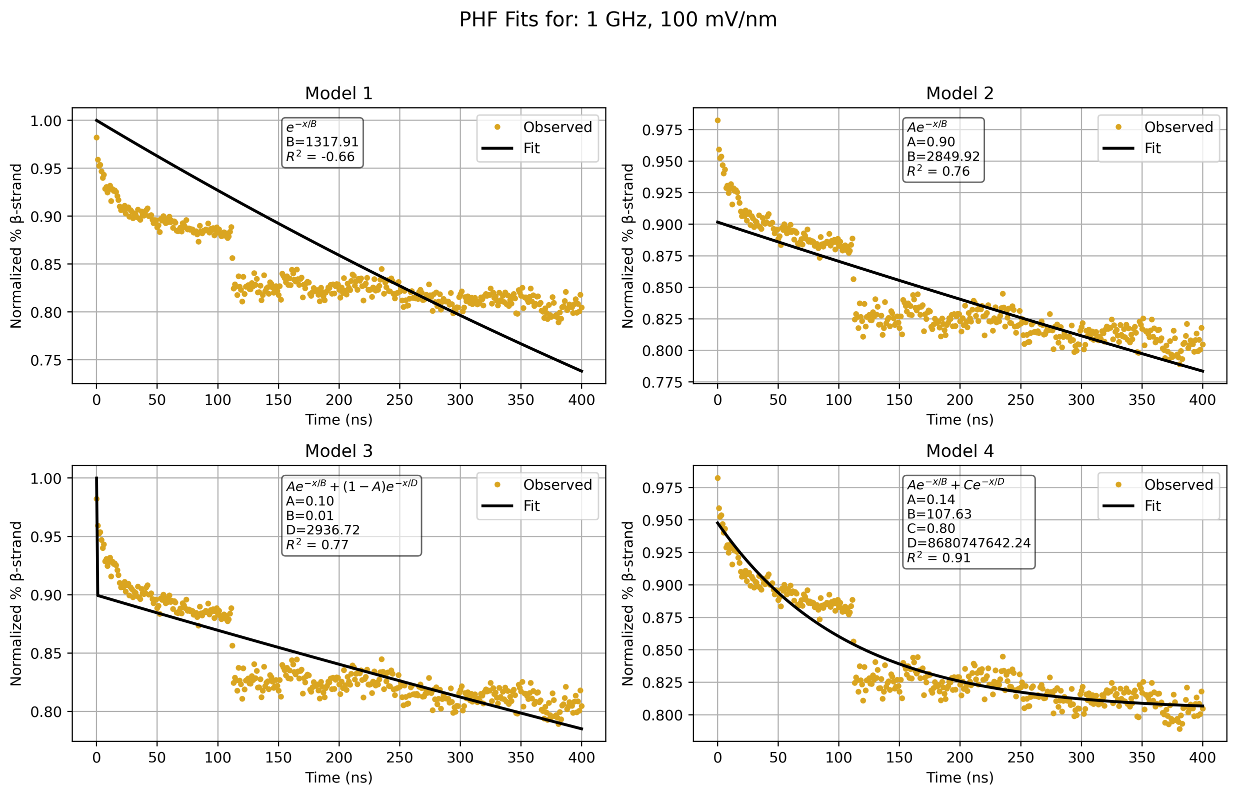

Figure S 60: Exponential fitting methods based on four different models of the β-strand content for the peptides of PHF with a 100 mV/nm oeEF oscillating at 1 GHz. Same visualization as Figure S58

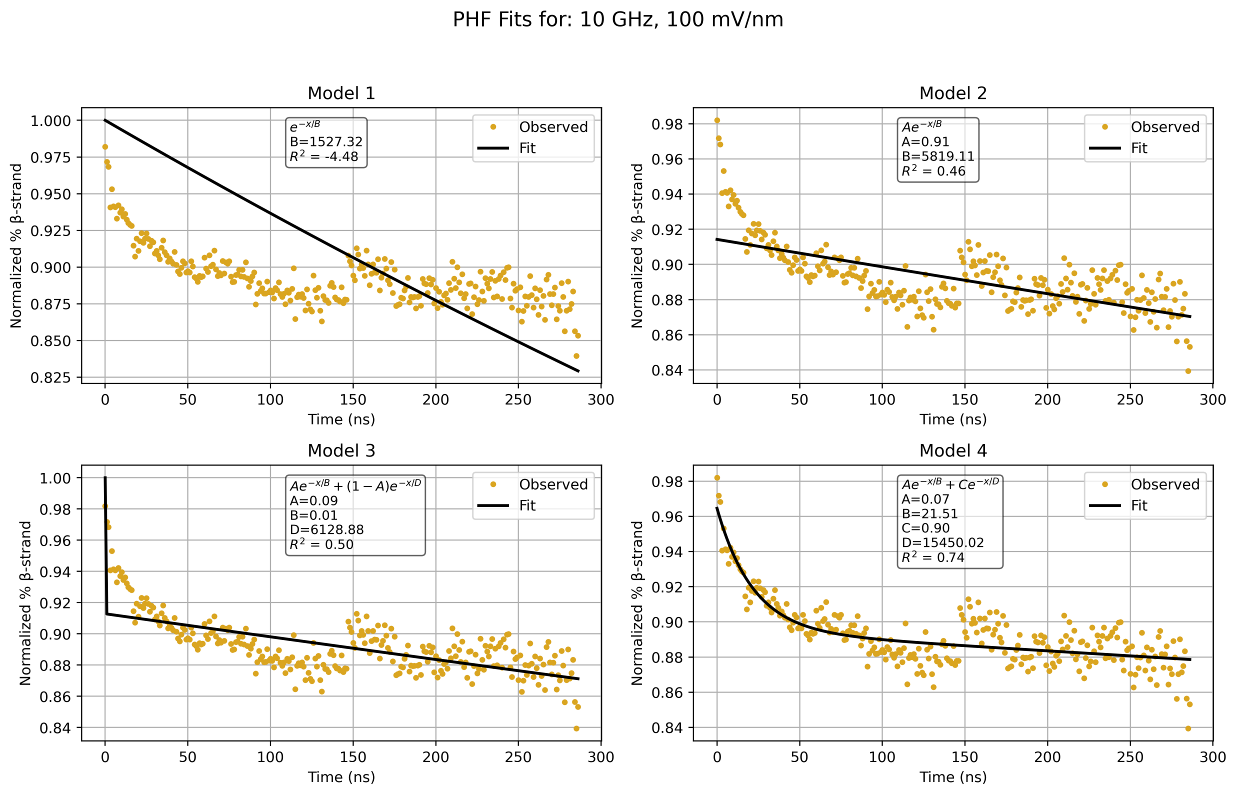

Figure S 61: Exponential fitting methods based on four different models of the β-strand content for the peptides of PHF with a 100 mV/nm oeEF oscillating at 10 GHz. Same visualization as Figure S58

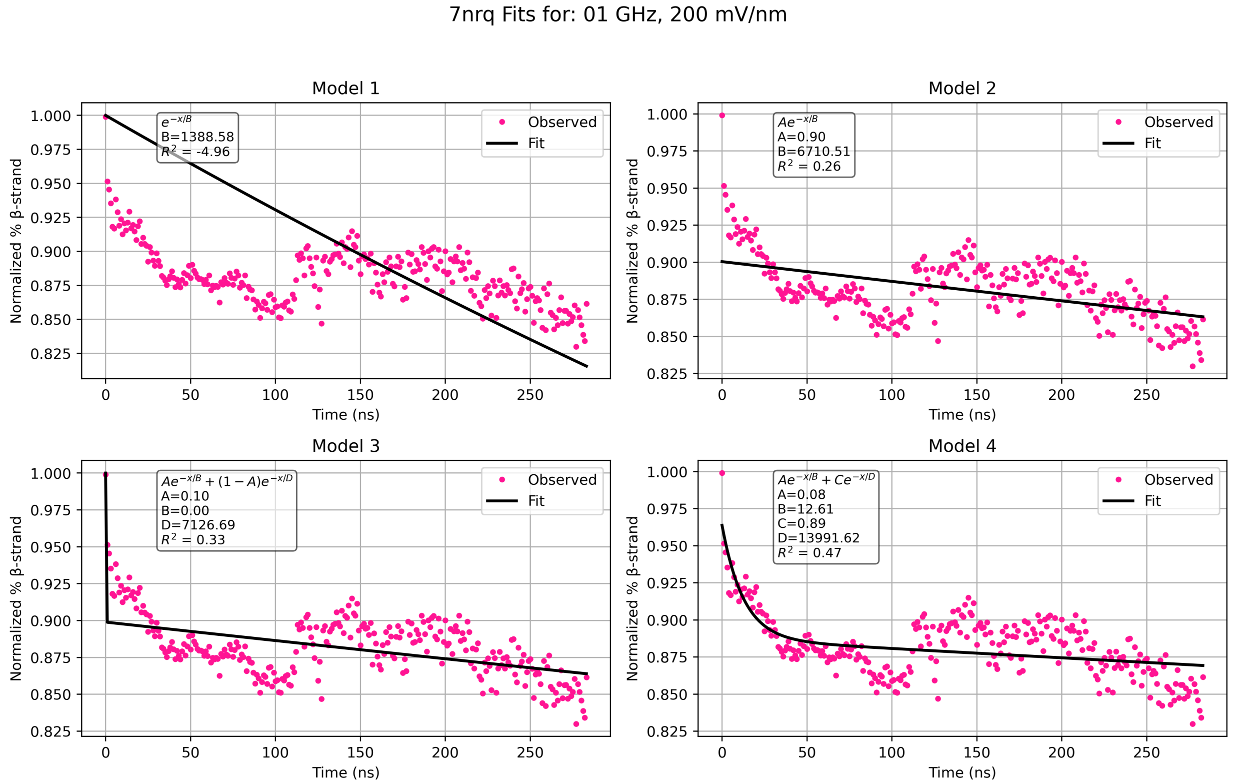

Figure S 62: Exponential fitting methods based on four different models of the β-strand content for the peptides of PHF with a 200 mV/nm oeEF oscillating at 0.1 GHz. Same visualization as Figure S58

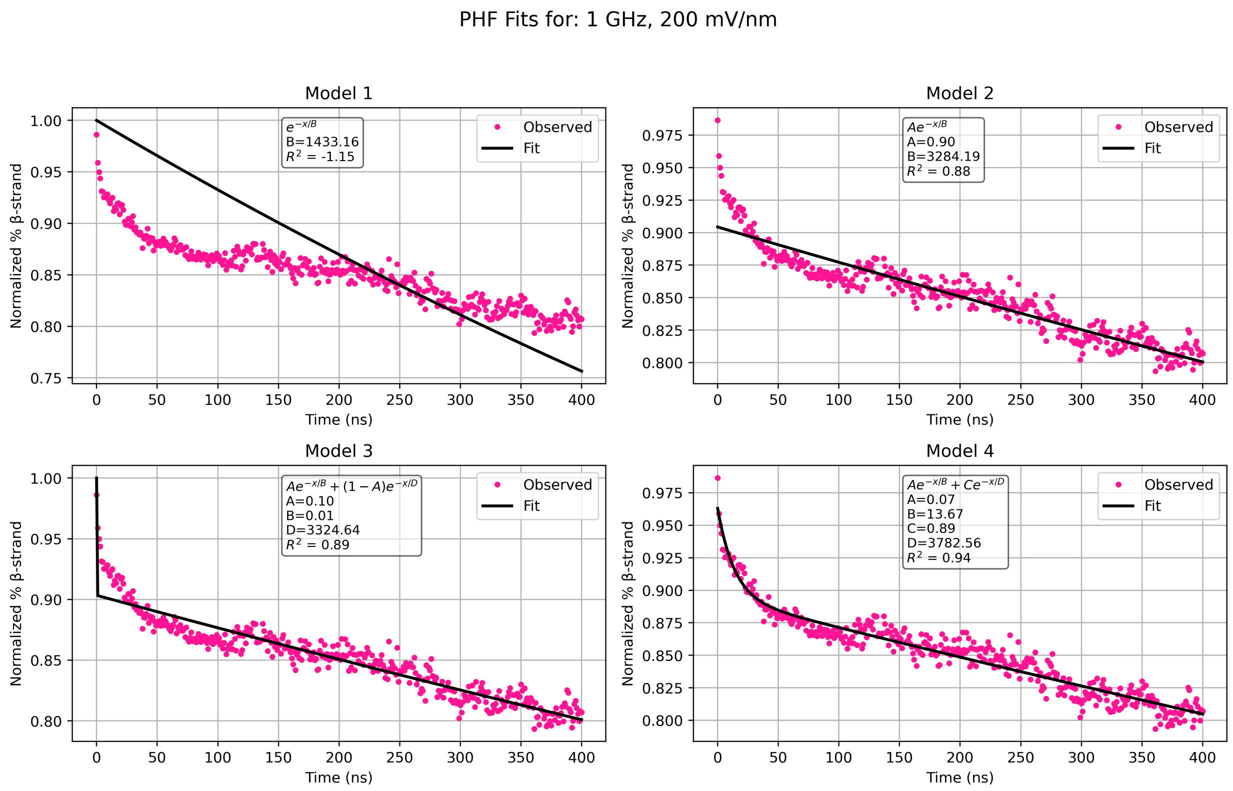

Figure S 63: Exponential fitting methods based on four different models of the β-strand content for the peptides of PHF with a 200 mV/nm oeEF oscillating at 1 GHz. Same visualization as Figure S58

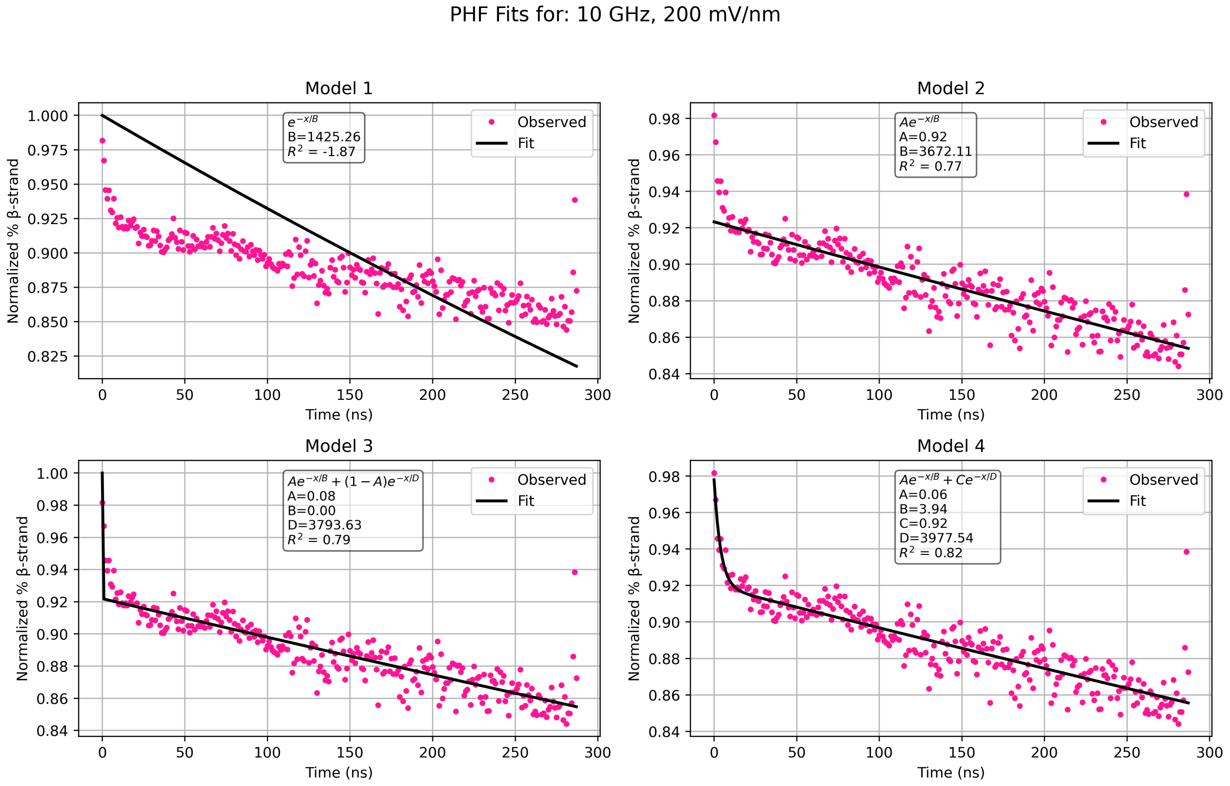

Figure S 64: Exponential fitting methods based on four different models of the β-strand content for the peptides of PHF with a 200 mV/nm oeEF oscillating at 10 GHz. Same visualization as Figure S58

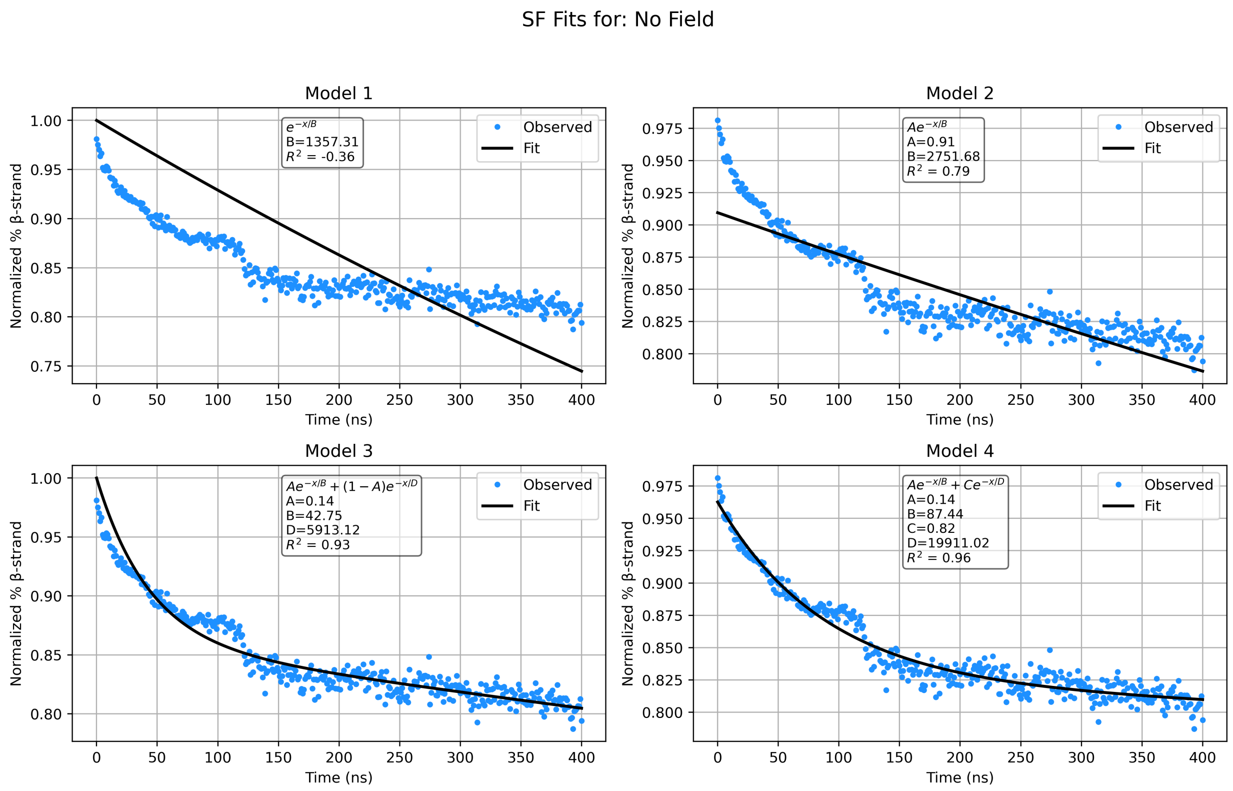

Figure S 65: Exponential fitting methods based on four different models of the β-strand content for the peptides of SF without an oeEF. Same visualization as Figure S58

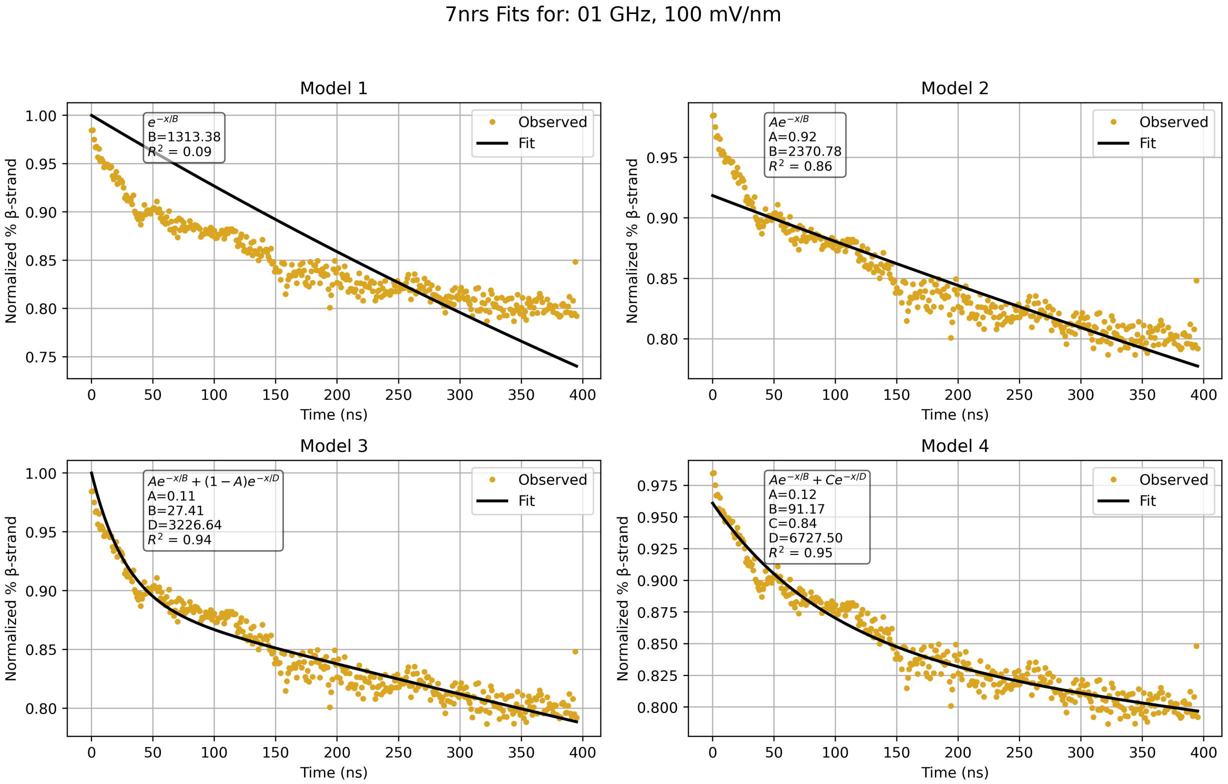

Figure S 66: Exponential fitting methods based on four different models of the β-strand content for the peptides of SF with a 100 mV/nm oeEF oscillating at 0.1 GHz. Same visualization as Figure S58

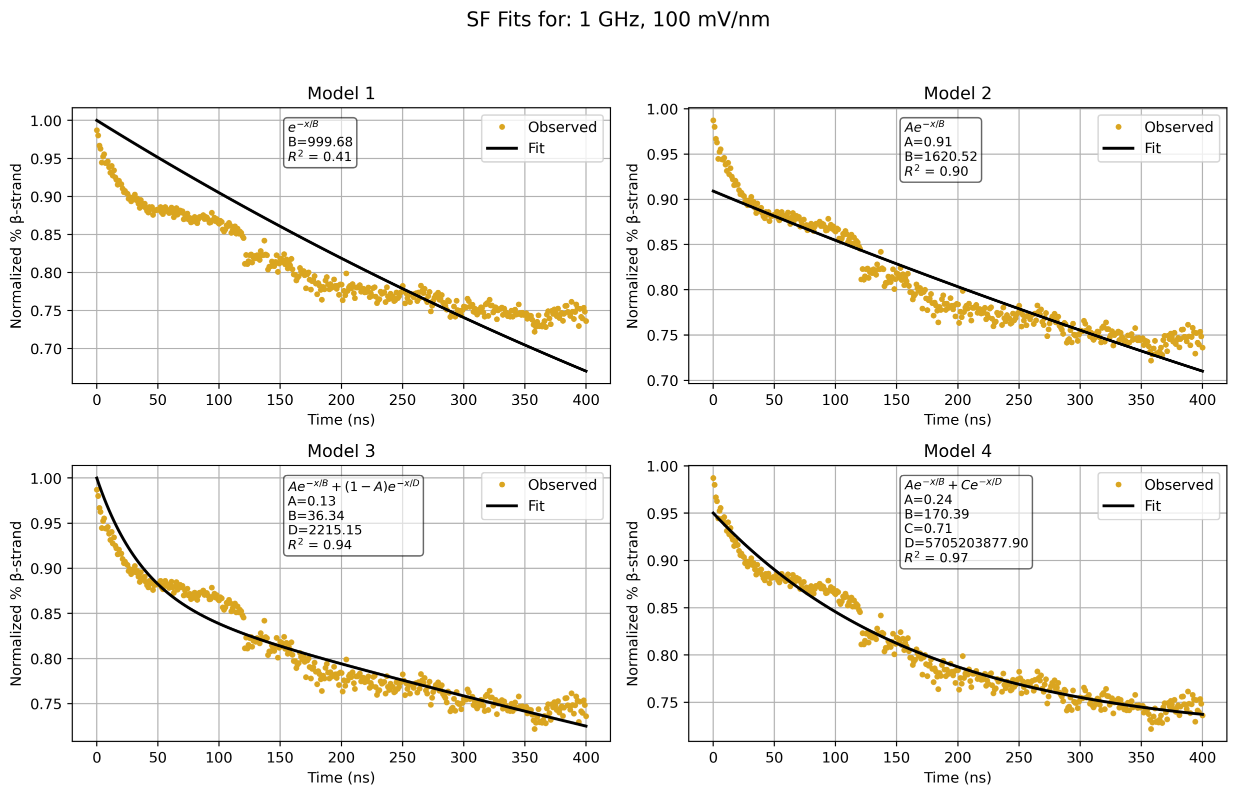

Figure S 67: Exponential fitting methods based on four different models of the β-strand content for the peptides of SF with a 100 mV/nm oeEF oscillating at 1 GHz. Same visualization as Figure S58

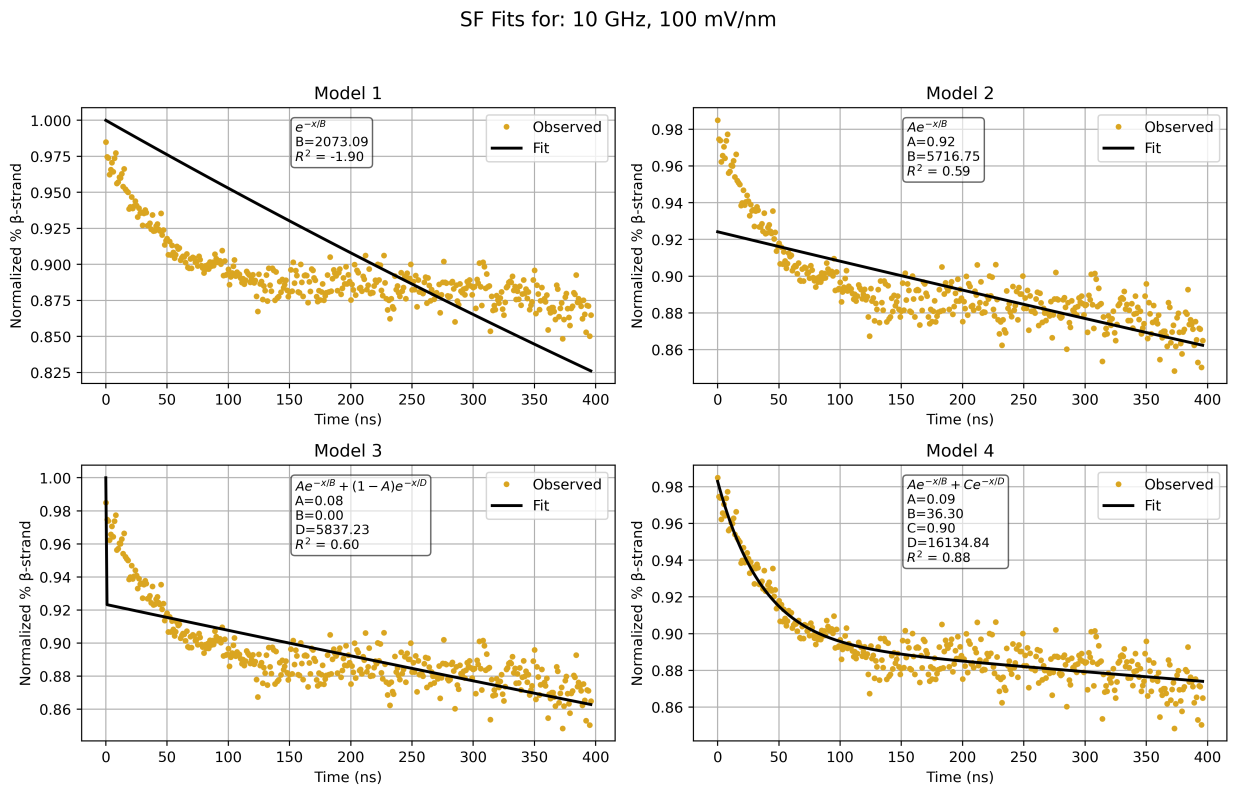

Figure S 68: Exponential fitting methods based on four different models of the β-strand content for the peptides of SF with a 100 mV/nm oeEF oscillating at 10 GHz. Same visualization as Figure S58

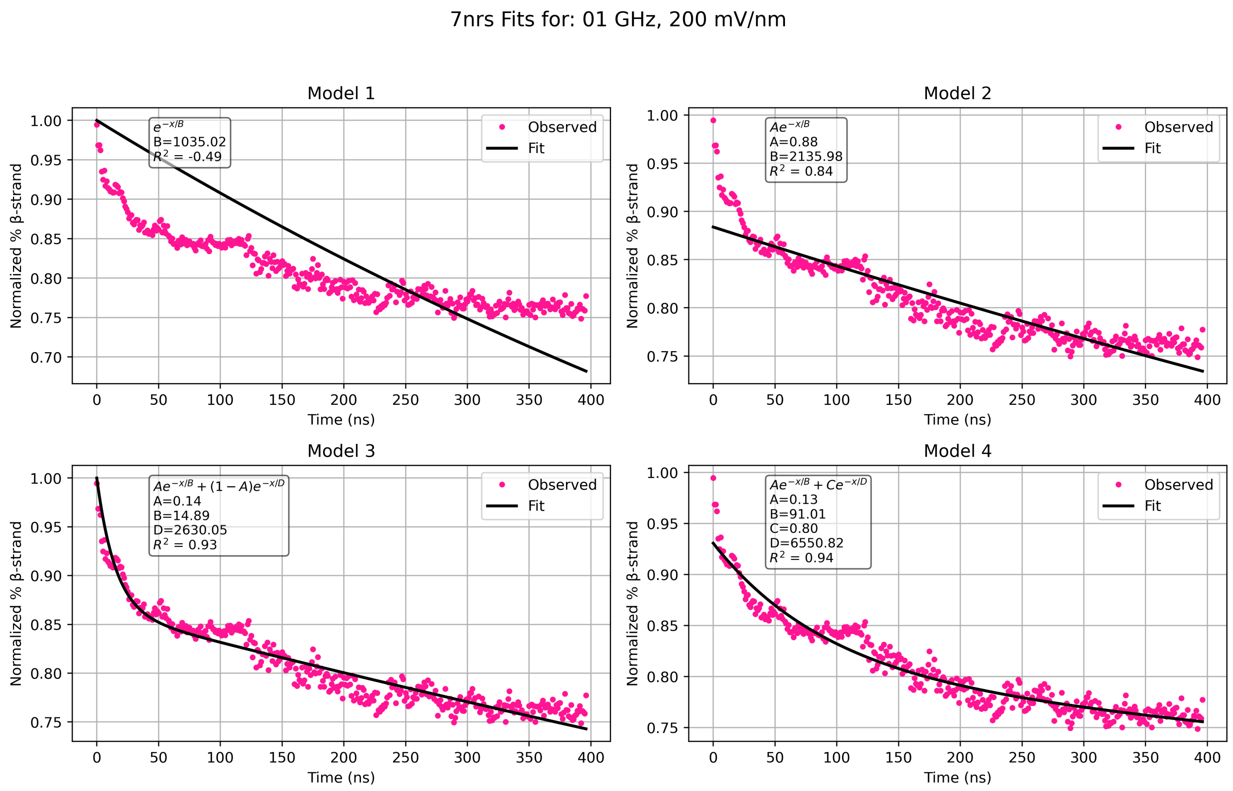

Figure S 69: Exponential fitting methods based on four different models of the β-strand content for the peptides of SF with a 200 mV/nm oeEF oscillating at 0.1 GHz. Same visualization as Figure S58

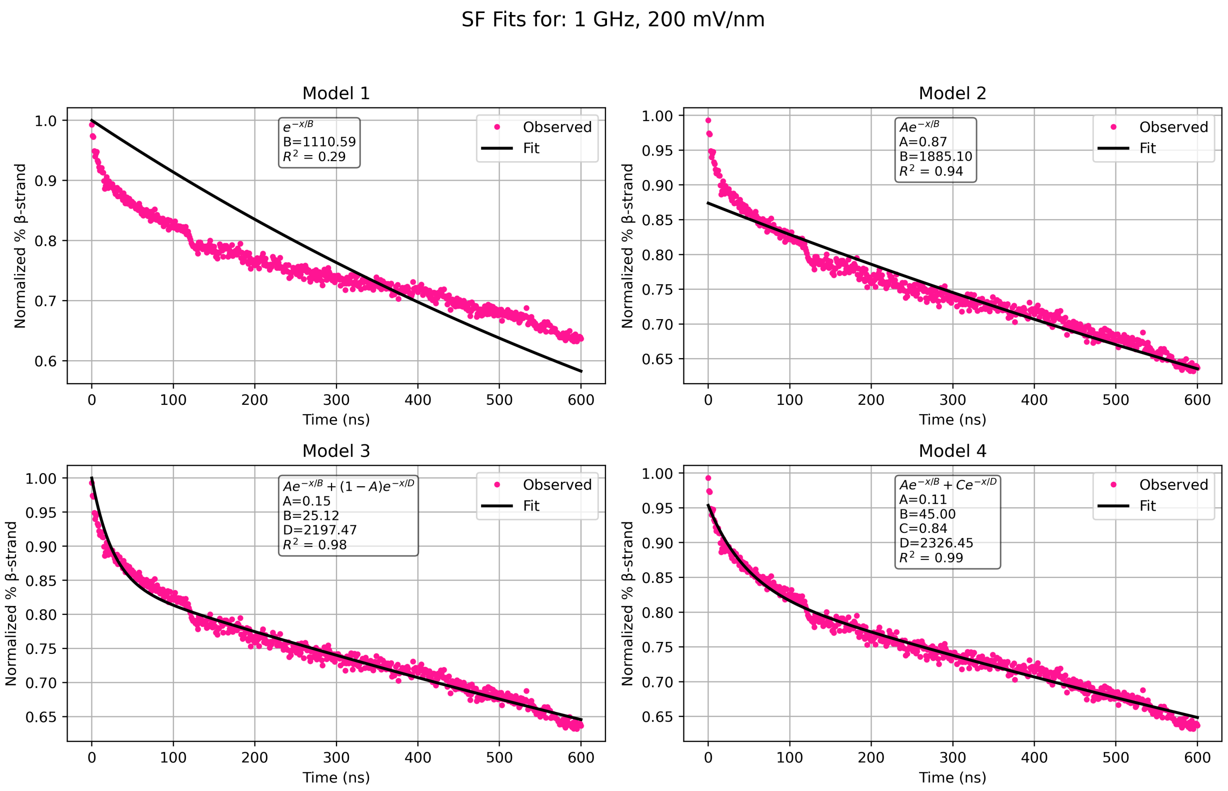

Figure S 70: Exponential fitting methods based on four different models of the β-strand content for the peptides of SF with a 200 mV/nm oeEF oscillating at 1 GHz. Same visualization as Figure S58

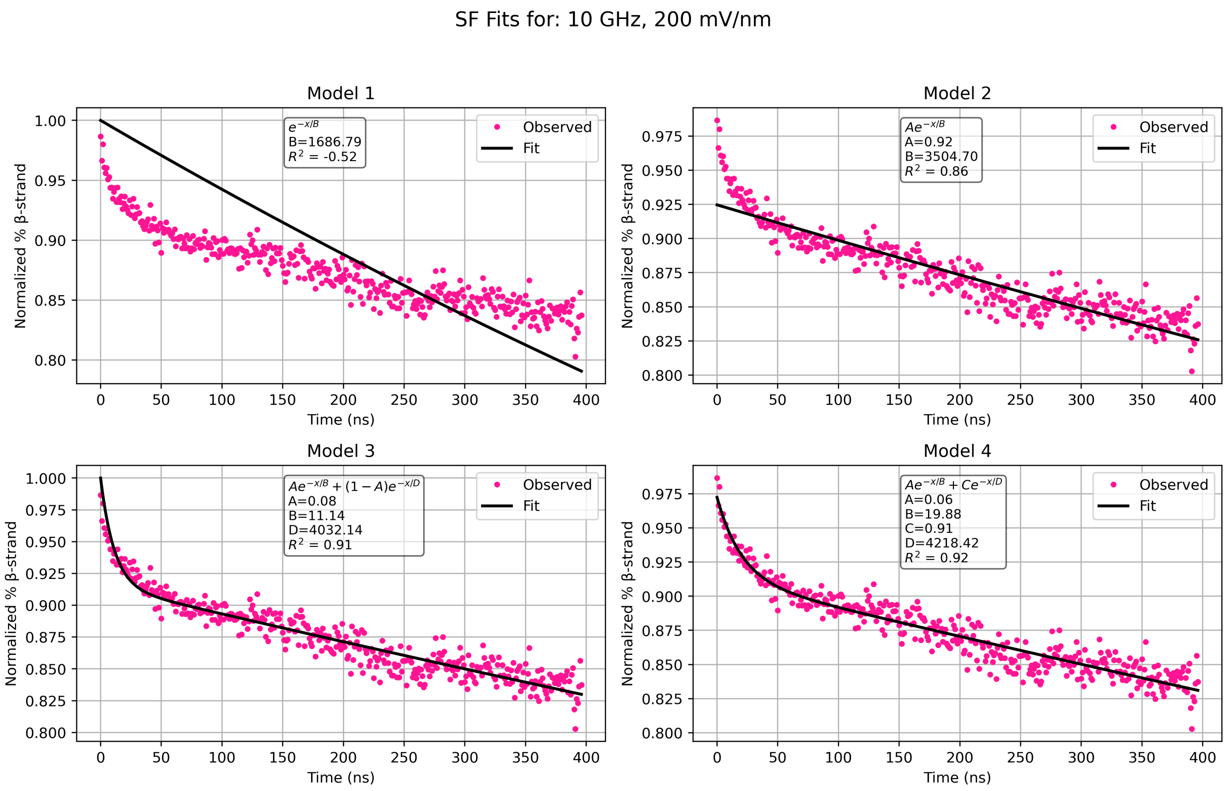

Figure S 71: peptides of SF with a 200 mV/nm oeEF oscillating at 10 GHz. Same visualization as Figure S58

Implicit solvent simulations

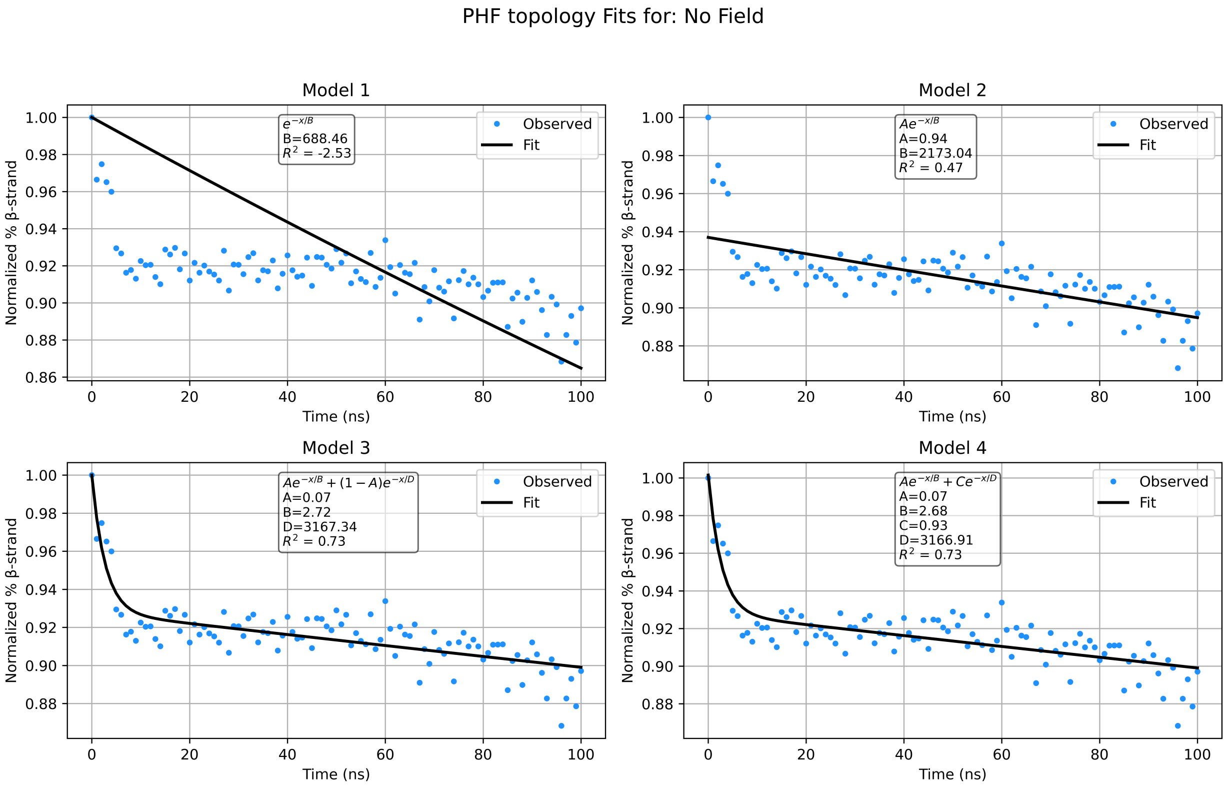

Figure S 72: Exponential fitting methods based on four different models of the β-strand content for the peptides of PHF without an oeEF. Same visualization as Figure S58

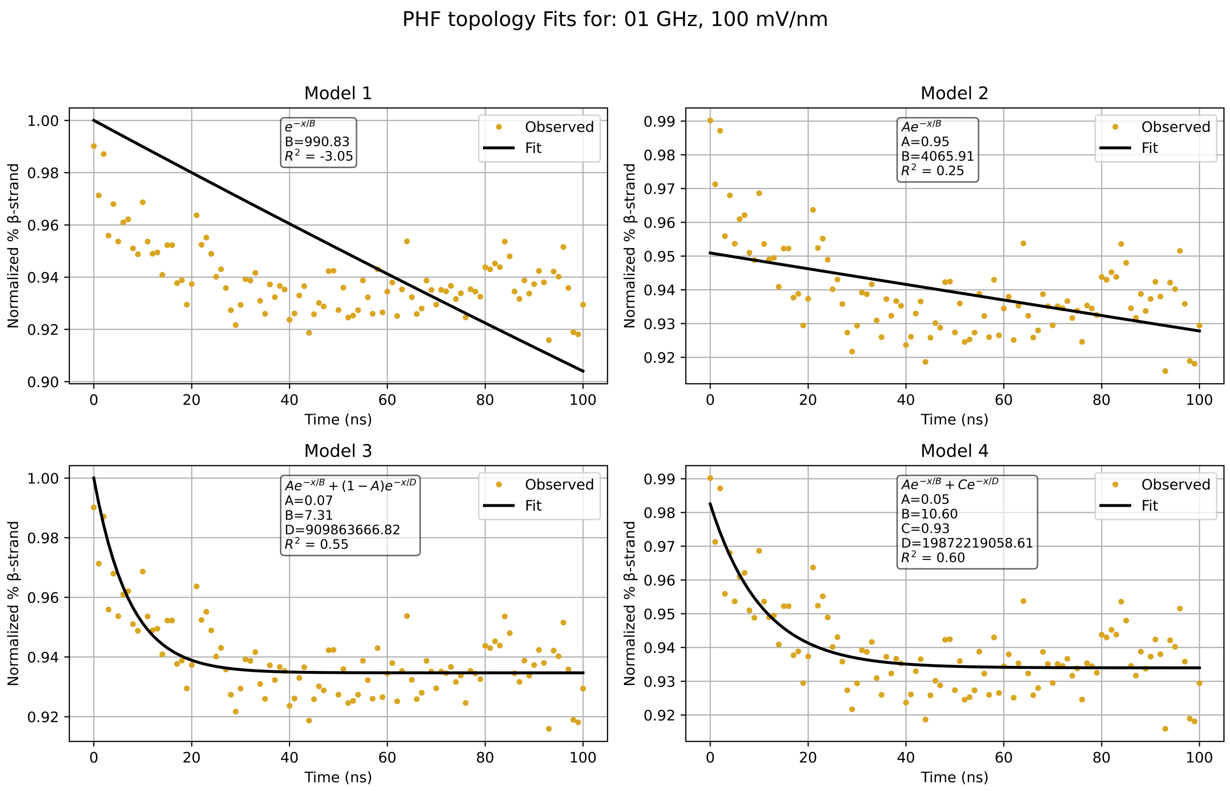

Figure S 73: Exponential fitting methods based on four different models of the β-strand content for the peptides of PHF with a 100 mV/nm oeEF oscillating at 0.1 GHz. Same visualization as Figure S58

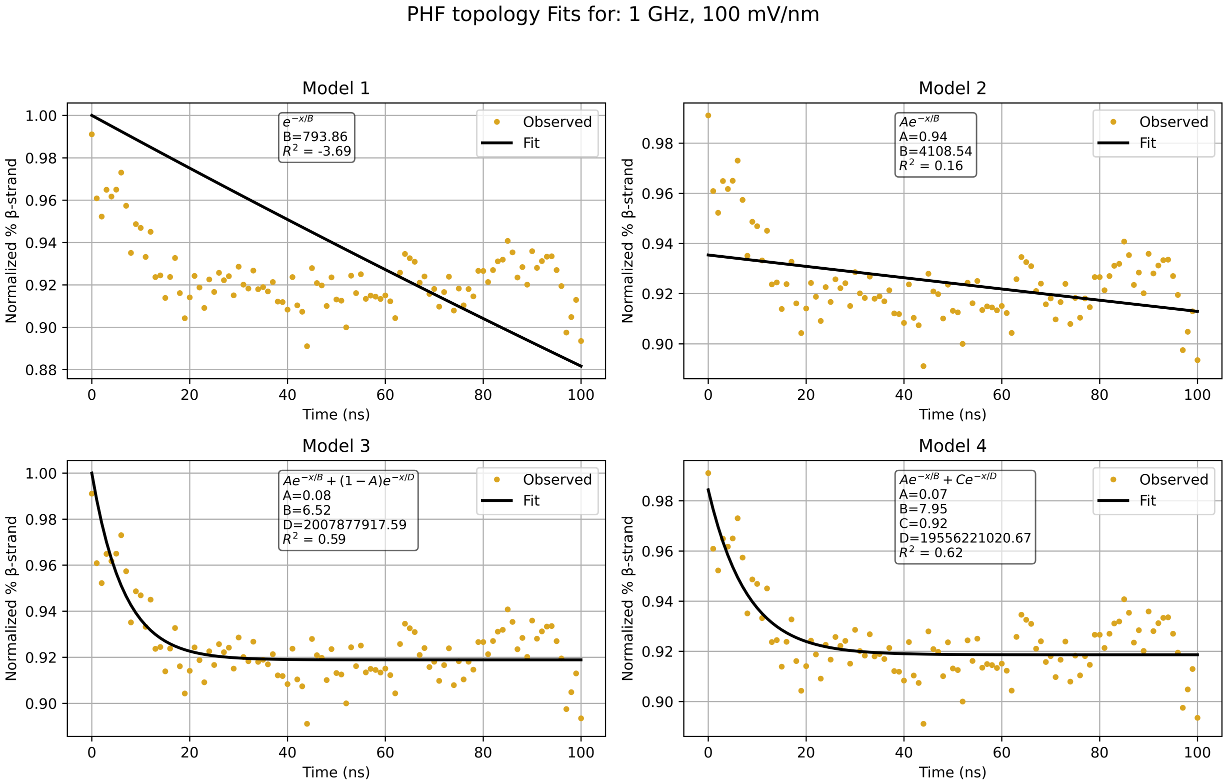

Figure S 74: Exponential fitting methods based on four different models of the β-strand content for the peptides of PHF with a 100 mV/nm oeEF oscillating at 1 GHz. Same visualization as Figure S58

It is noteworthy, that for both the 0.1 and 1 GHz systems at 100 mV/nm (Figures S73 and S74, respectively), the models above three parameters overfit the data and yield an extremely high decay constant. This is due to the simulation being too short to display a significant decay in the β-strand count. We use the two-parameter Model 2 in these two cases to calculate the half-life of the system.

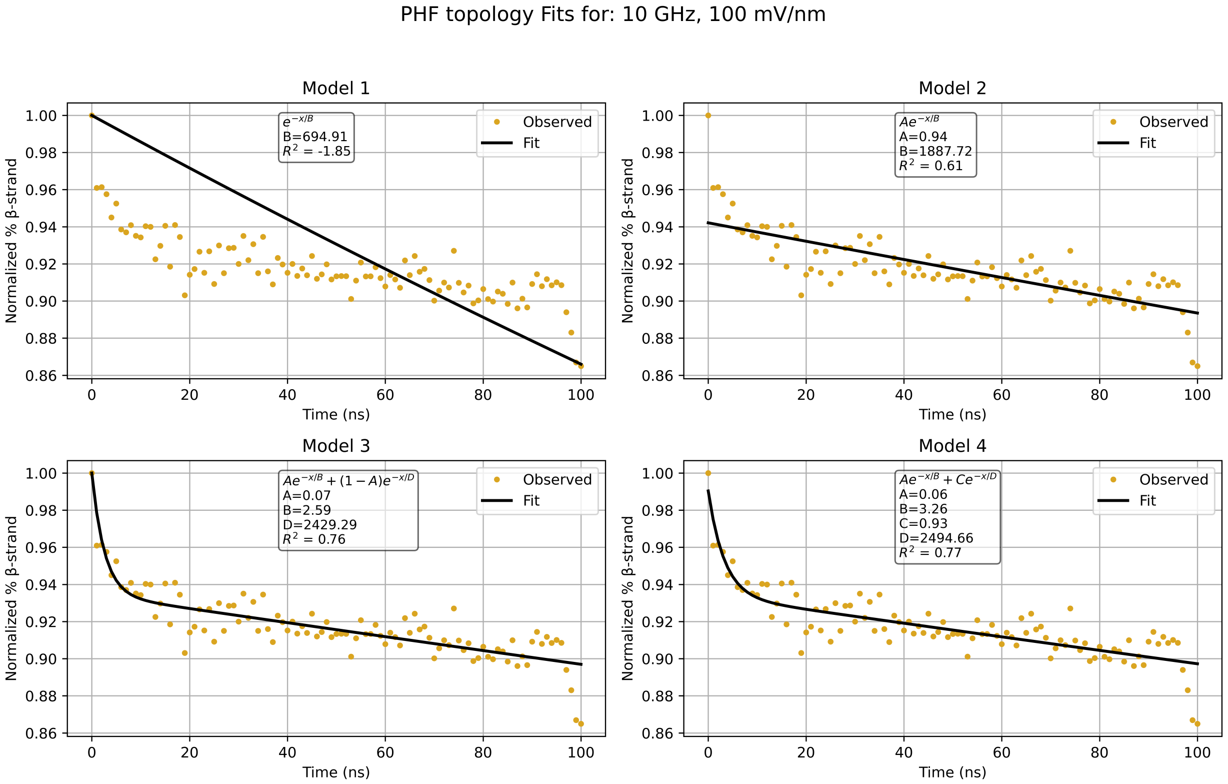

Figure S 75: Exponential fitting methods based on four different models of the β-strand content for the peptides of PHF with a 100 mV/nm oeEF oscillating at 10 GHz. Same visualization as Figure S58

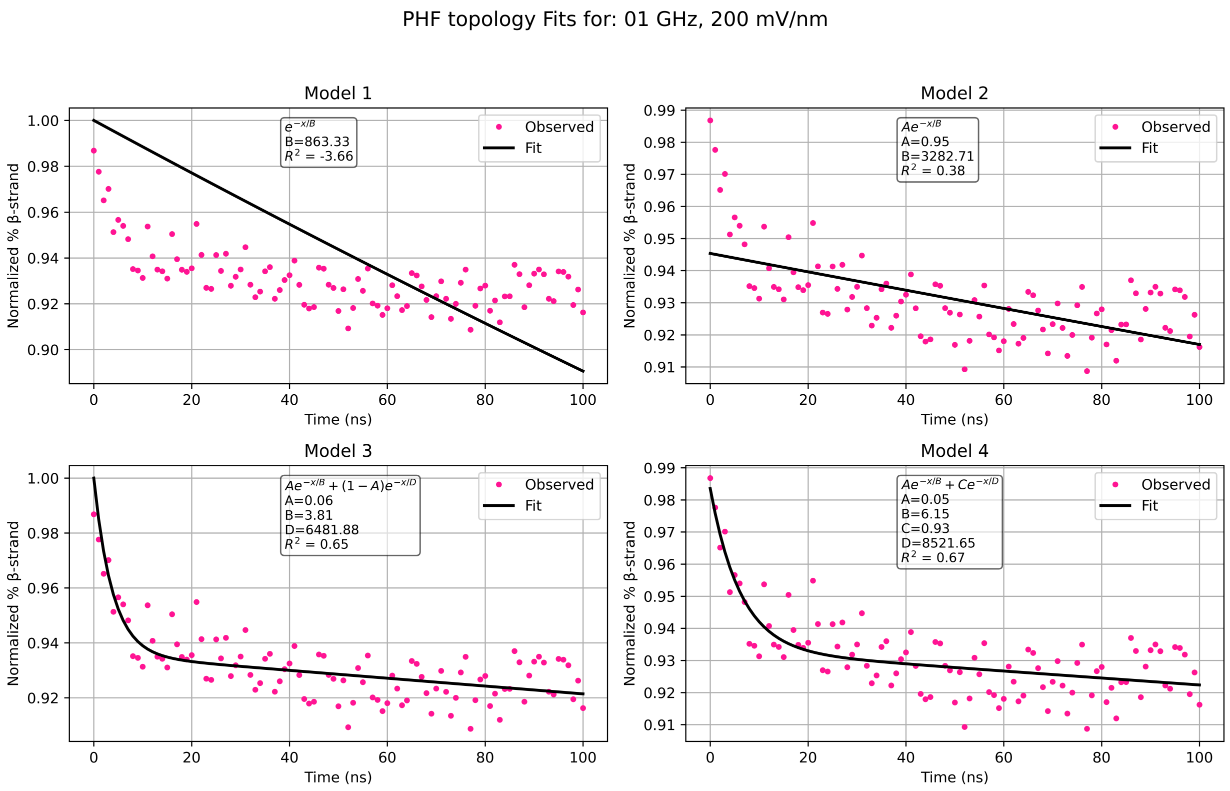

Figure S 76: Exponential fitting methods based on four different models of the β-strand content for the peptides of PHF with a 200 mV/nm oeEF oscillating at 0.1 GHz. Same visualization as Figure S58

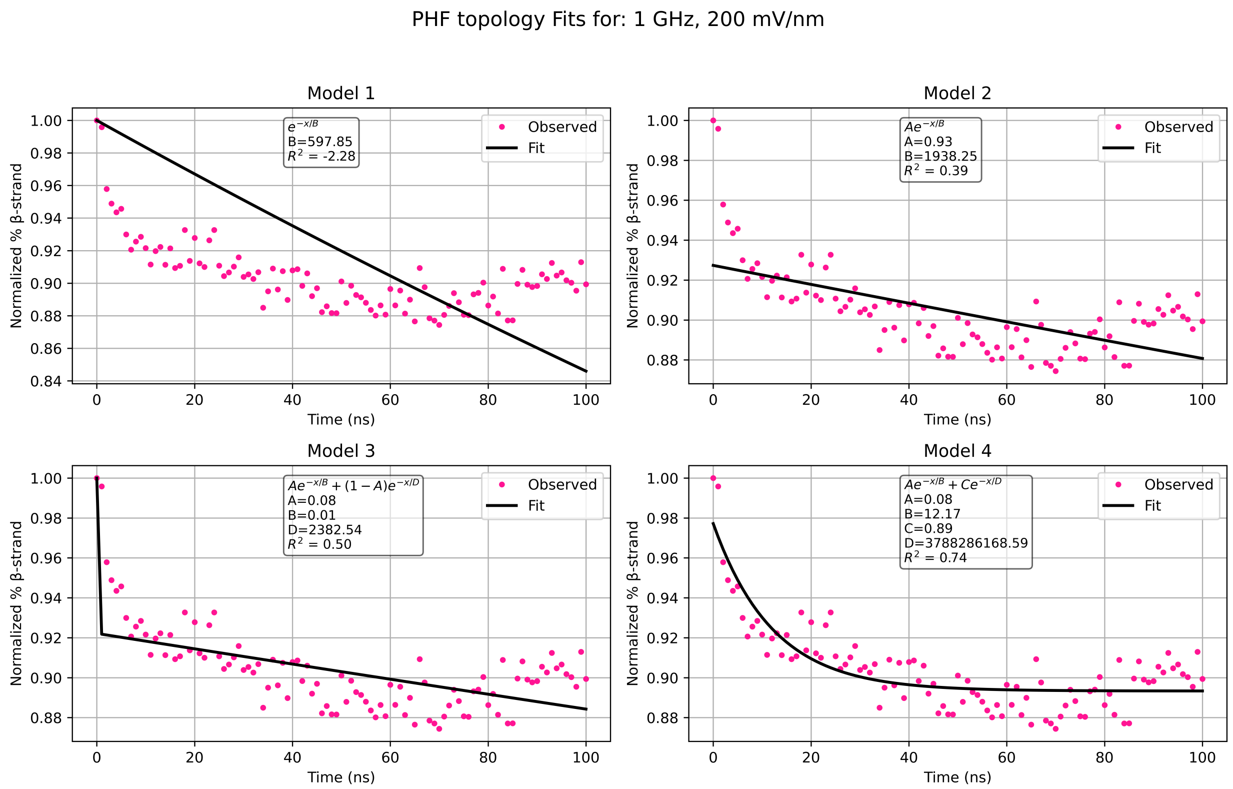

Figure S 77: Exponential fitting methods based on four different models of the β-strand content for the peptides of PHF with a 200 mV/nm oeEF oscillating at 1 GHz. Same visualization as Figure S58

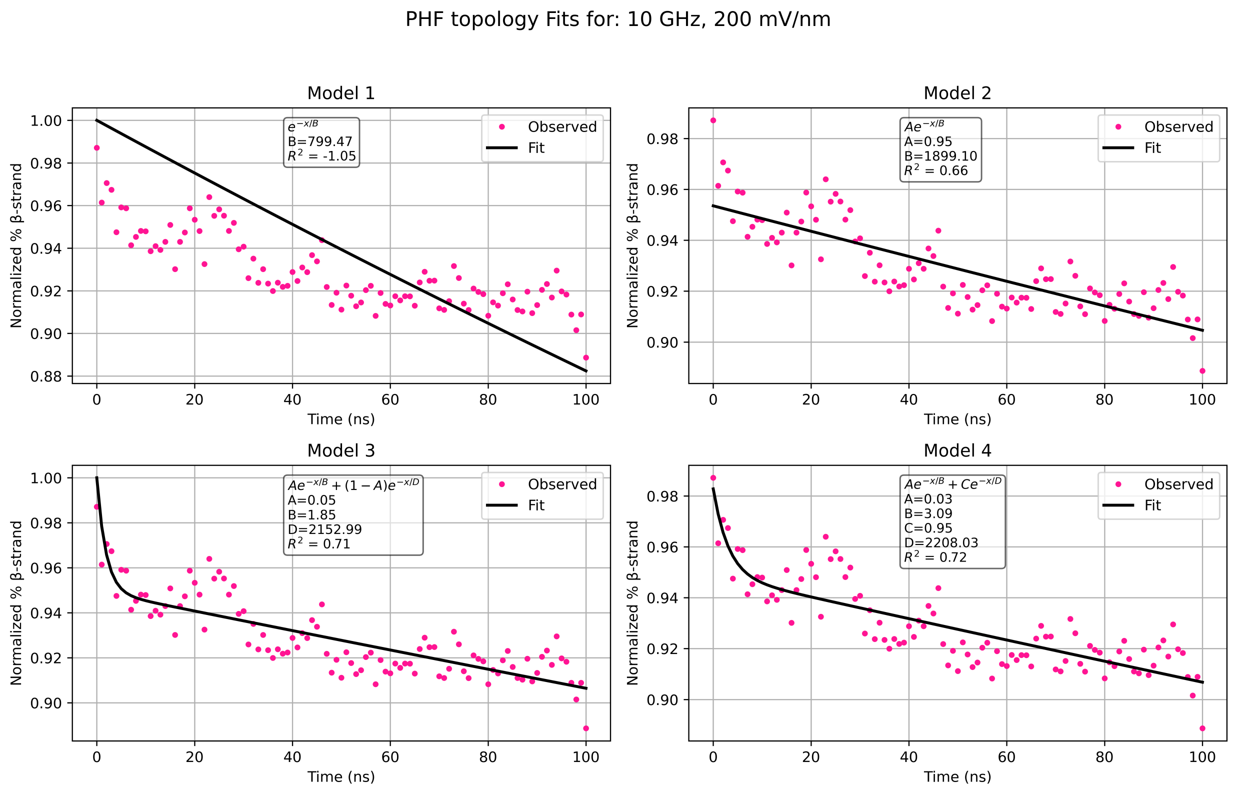

Figure S 78: Exponential fitting methods based on four different models of the β-strand content for the peptides of PHF with a 200 mV/nm oeEF oscillating at 10 GHz. Same visualization as Figure S58

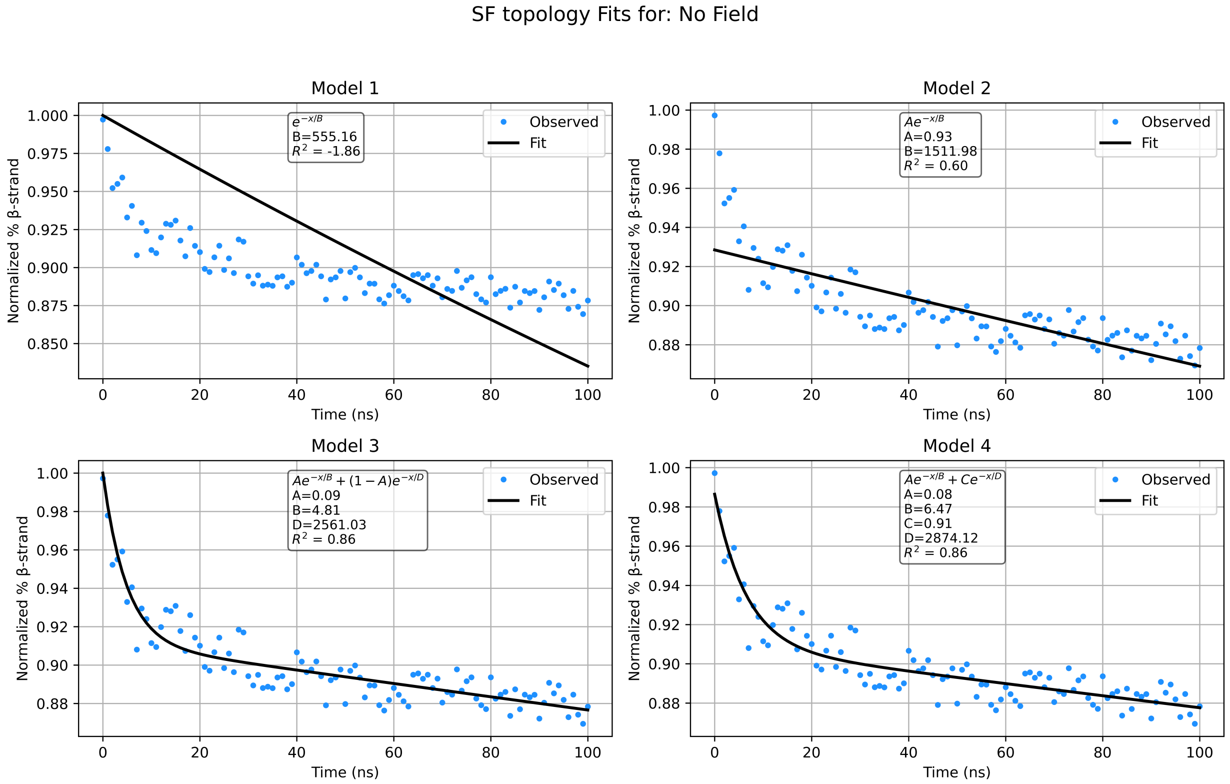

Figure S 79: Exponential fitting methods based on four different models of the β-strand content for the peptides of SF without an oeEF. Same visualization as Figure S58

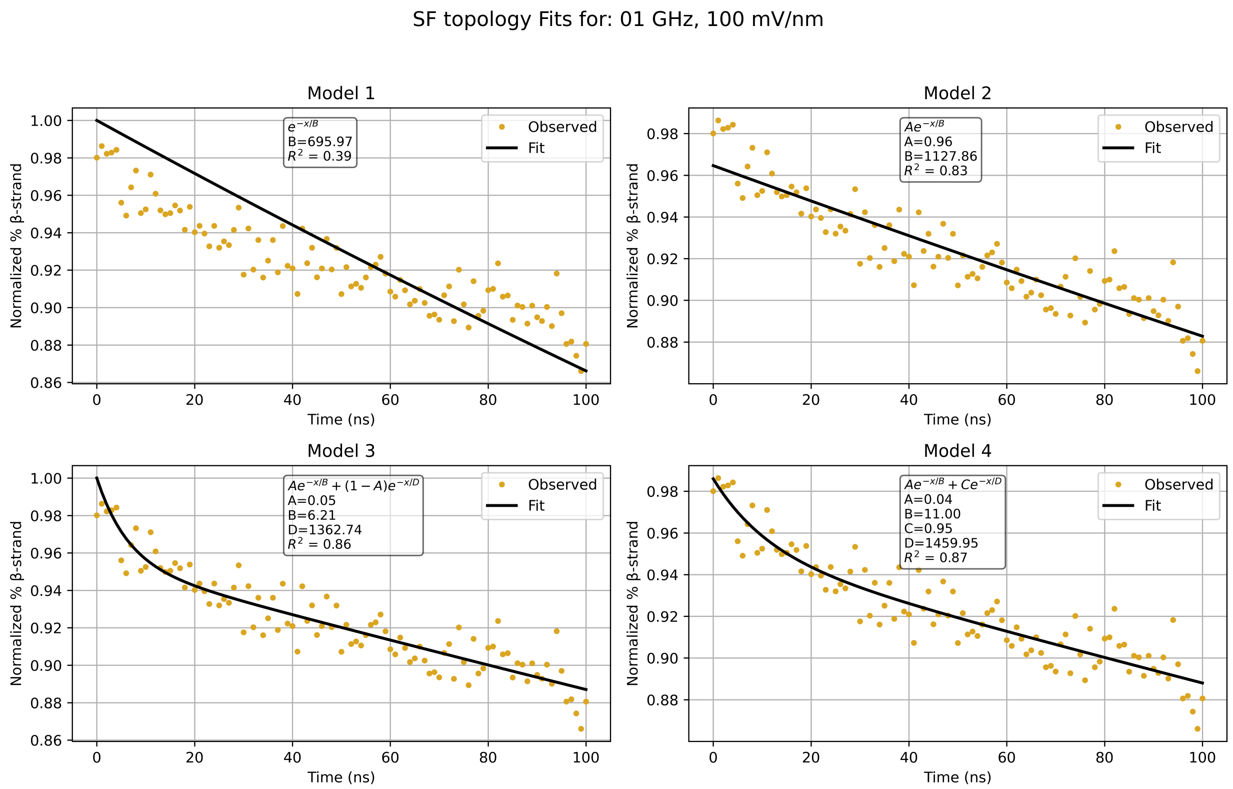

Figure S 80: Exponential fitting methods based on four different models of the β-strand content for the peptides of SF with a 100 mV/nm oeEF oscillating at 0.1 GHz. Same visualization as Figure S58

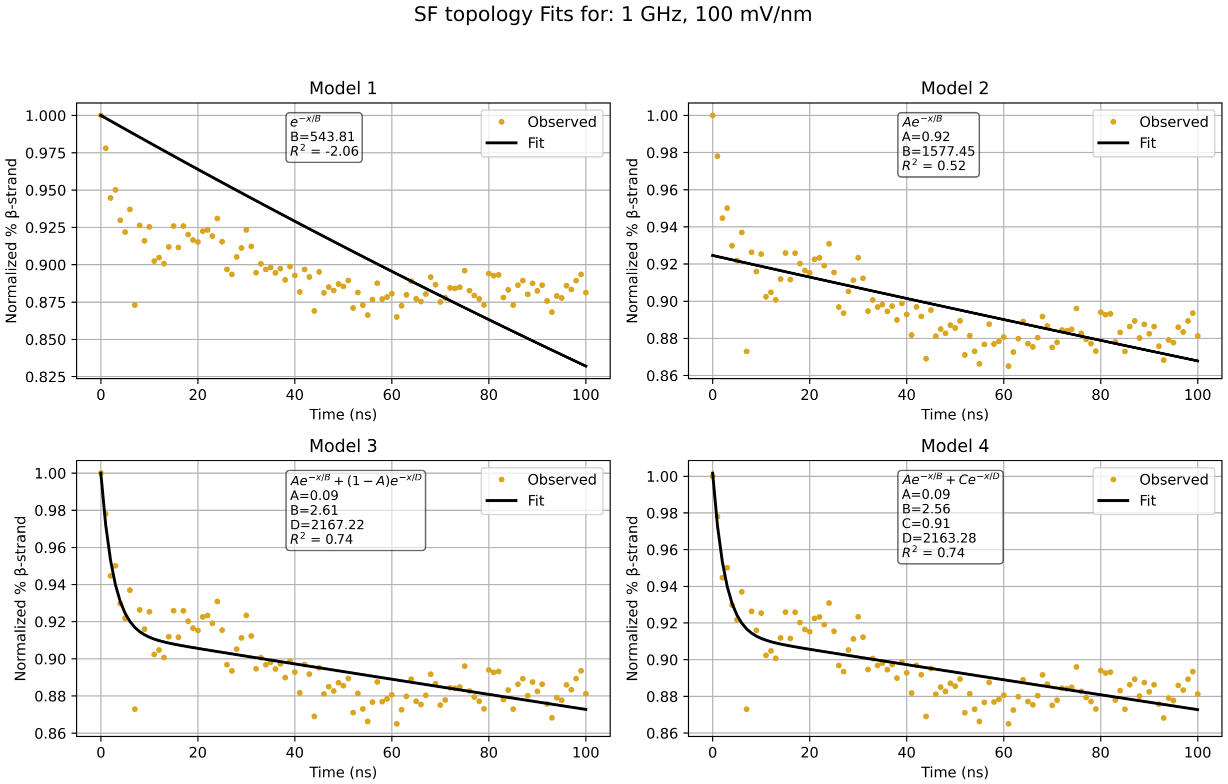

Figure S 81: Exponential fitting methods based on four different models of the β-strand content for the peptides of SF with a 100 mV/nm oeEF oscillating at 1 GHz. Same visualization as Figure S58

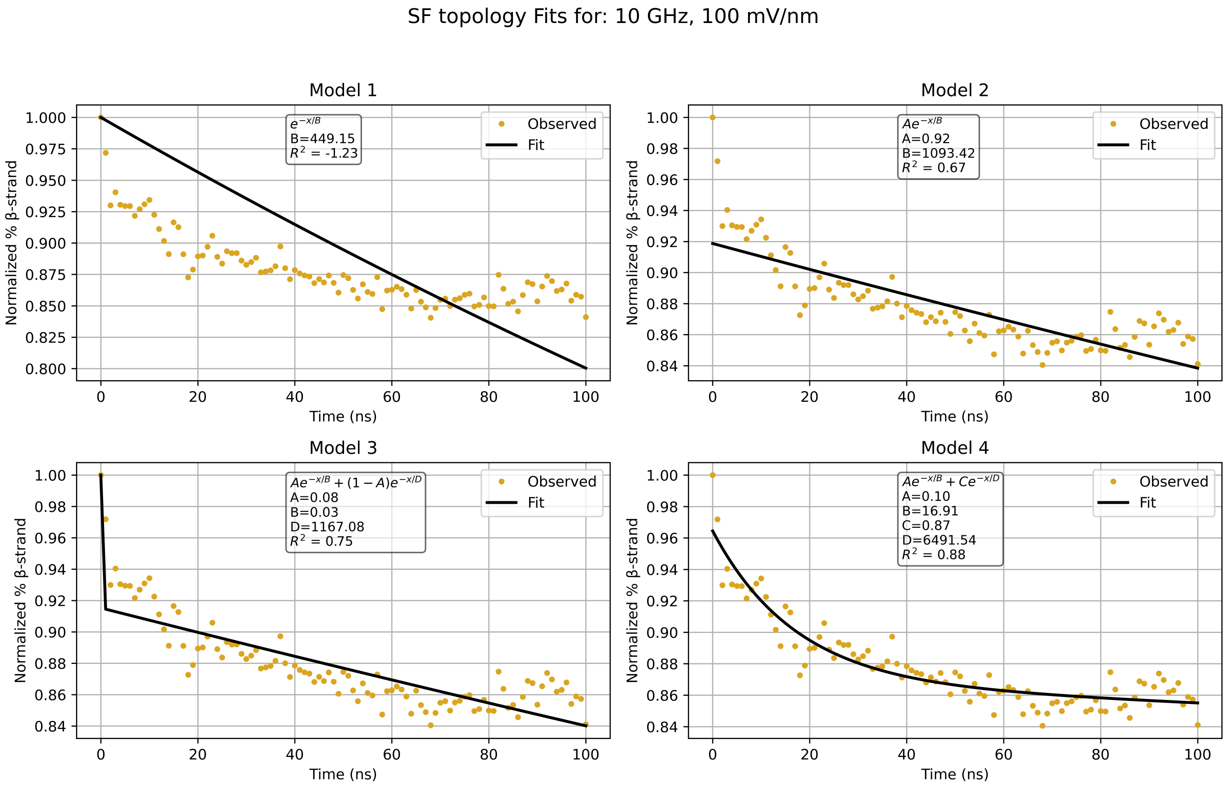

Figure S 82: Exponential fitting methods based on four different models of the β-strand content for the peptides of SF with a 100 mV/nm oeEF oscillating at 10 GHz. Same visualization as Figure S58

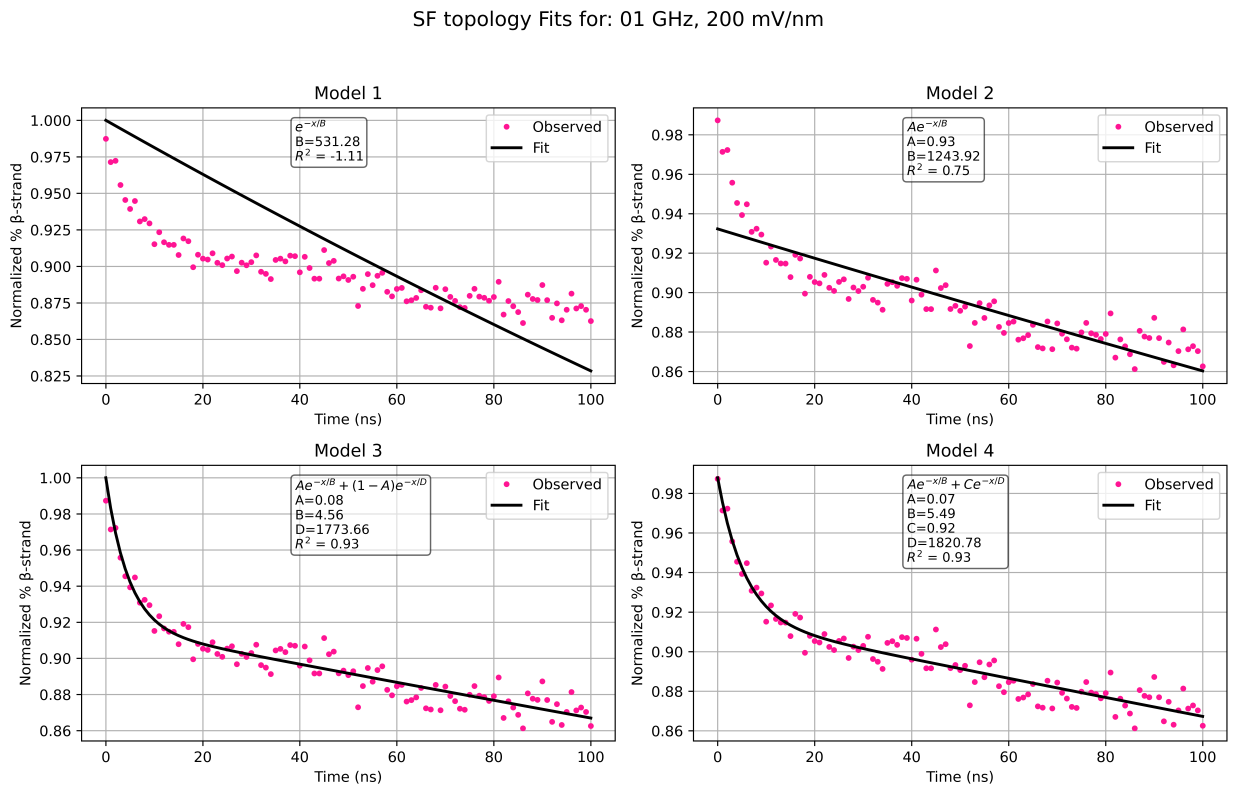

Figure S 83: Exponential fitting methods based on four different models of the β-strand content for the peptides of SF with a 200 mV/nm oeEF oscillating at 0.1 GHz. Same visualization as Figure S58

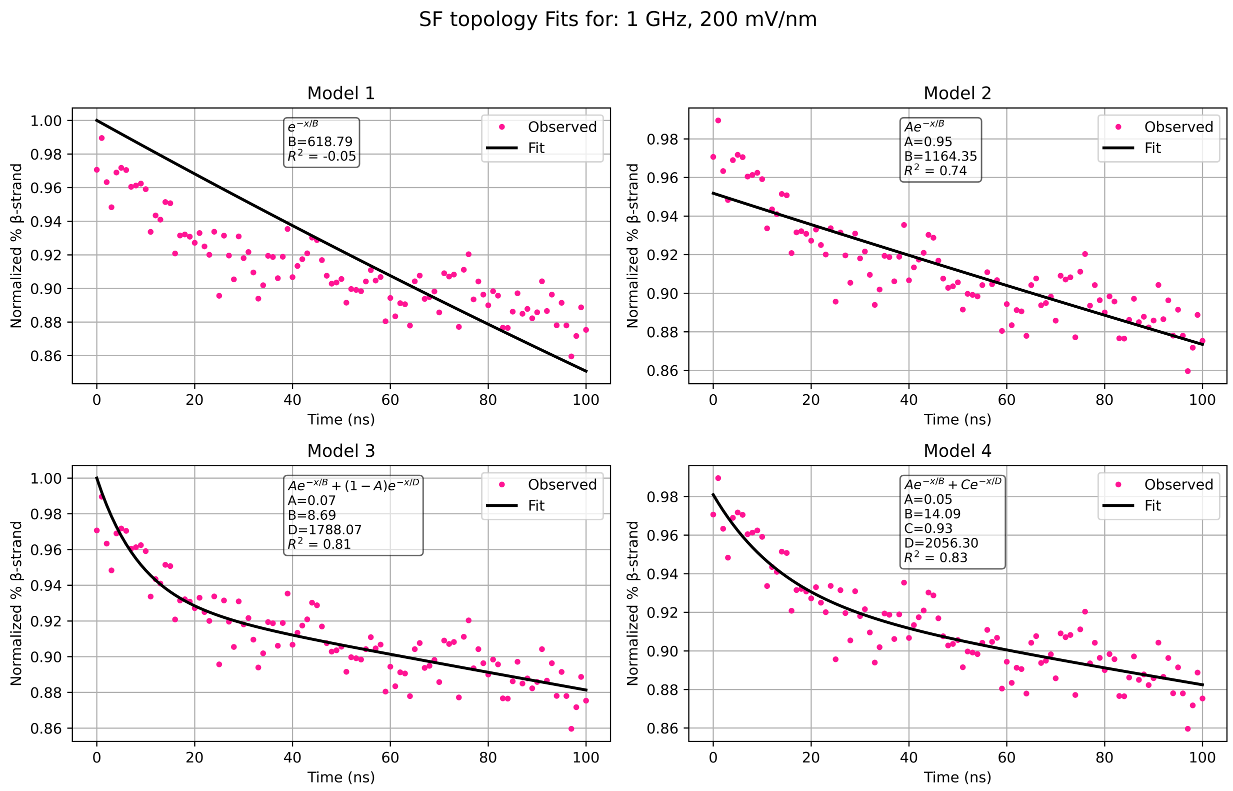

Figure S 84: Exponential fitting methods based on four different models of the β-strand content for the peptides of SF with a 200 mV/nm oeEF oscillating at 1 GHz. Same visualization as Figure S58

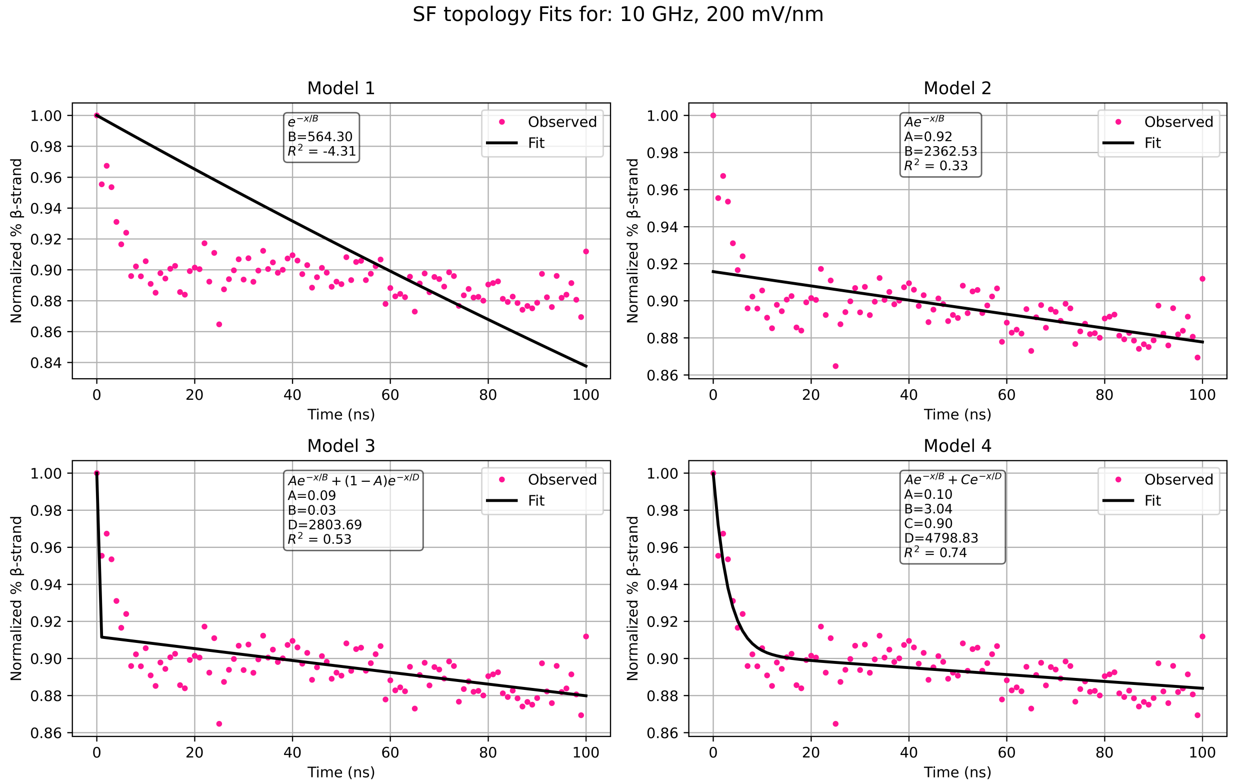

Figure S 85: Exponential fitting methods based on four different models of the β-strand content for the peptides of SF with a 200 mV/nm oeEF oscillating at 10 GHz. Same visualization as Figure S58

Simulations with 7-EFE-9 fragment

Explicit solvent simulations

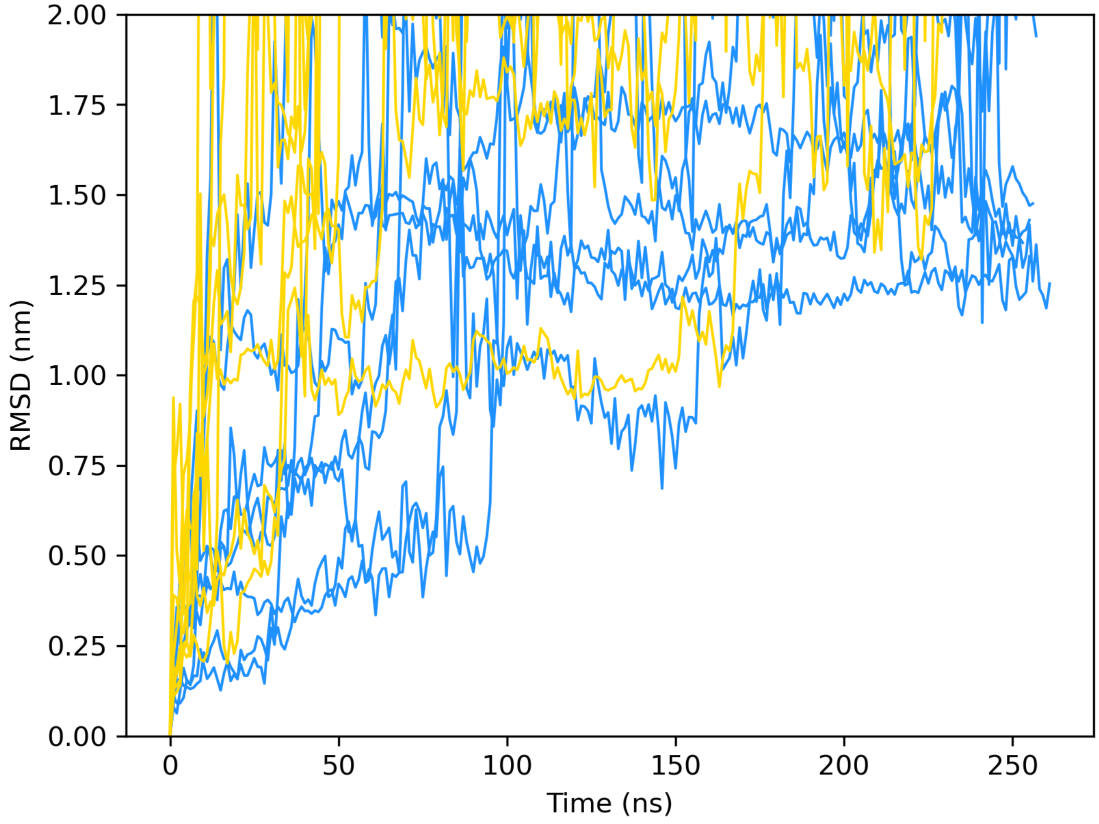

Figure S 86: Root Mean Square Deviation of 7-EFE-9 fragment in SF topology.

Analysis of proper dihedral angle to assess Flying Ice Cube Artifact

When an electric field is applied to a system, it leads all the charged particles to move accordingly to its direction, frequency and strength. The function called from the simulations software used in the present study is a periodic oscillation. Its presence cause the charged particles and everything attached to them to move and accelerate or decelerate according to its period while translating in space. In an explicit solvent system, the presence of water molecules creates friction that limits the translation and does not strongly affect the equipar-

tition theorem of energy to the proteic part of the system. This is different in the case of implicit solvent simulations, as a mean field is present to resemble the water environment. There is a risk that a large part of the kinetic energy of the molecules is converted into translational energy, causing a rigid body motion where the vibrational contribution is basically negligible. From this would arise an effect that goes under the name of "Flying Ice Cube Artifact".[1] We tested that such a phenomenon was not present in our simulation studies by observing any presence of periodicity in the proper dihedral angles of our simulated systems.

Implicit solvent simulations

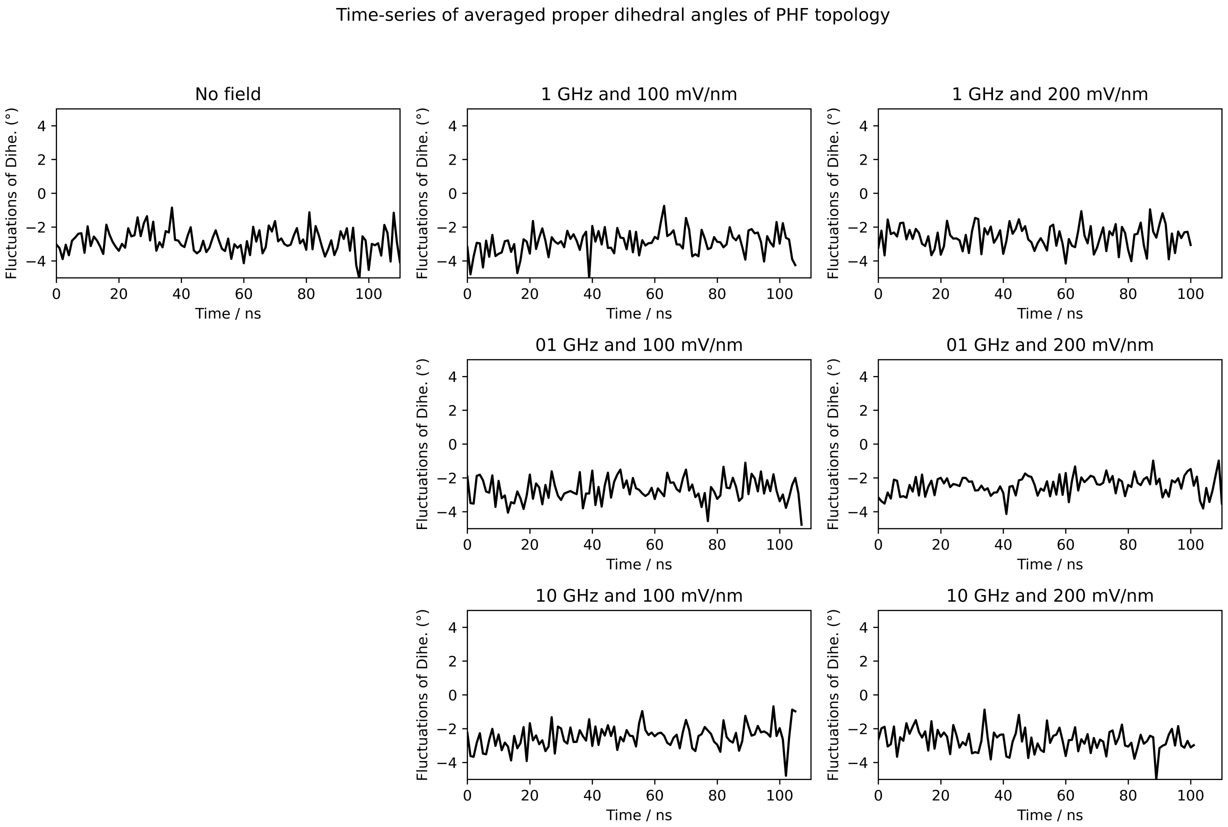

Figure S 87: Time-resolved average of all the proper dihedral angles (averaged per replica) of the PHF topology in a window of 1 ns at all the combinations of oeEF frequency and strength. The dihedral angles are plotted in the range -180 and +180 degrees. No periodic trends in average dihedral angle over time could indicates electric field-induced "Flying Ice Cube Artifact".

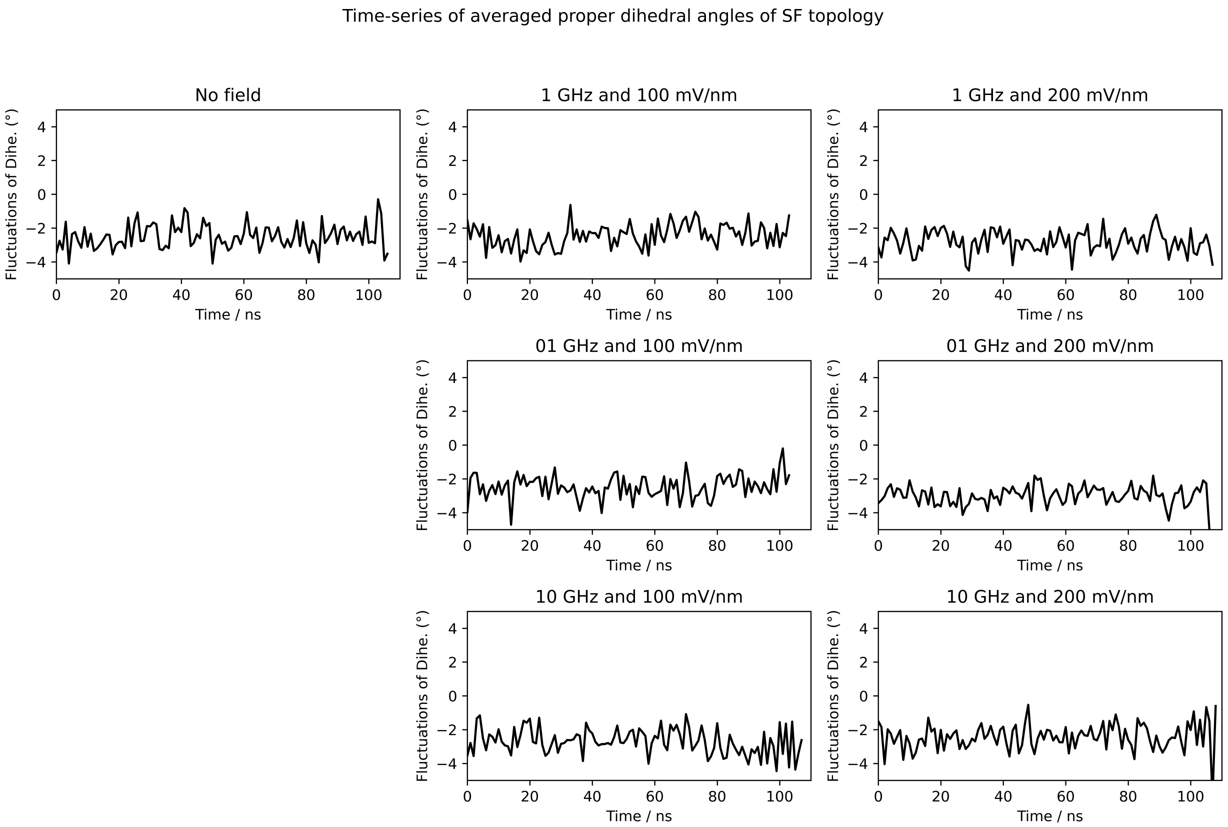

Figure S 88: Time-resolved average of all the proper dihedral angles (averaged per replica) of the PHF topology in a window of 1 ns at all the combinations of oeEF frequency and strength. Same visualization as Figure S87

References

(1) Harvey, Stephen C. and Tan, Robert K.-Z. and Cheatham III, Thomas E. *The flying ice cube: Velocity rescaling in molecular dynamics leads to violation of energy equipartition*, Journal of Computational Chemistry 1998, 19, 726-740.
